# Development of Novel Small-Molecule Targeting SCN1A-Associated Severe Myoclonic Epilepsy of Infancy

**DOI:** 10.1021/acs.jmedchem.5c03293

**Published:** 2026-01-23

**Authors:** Dong Gun Kim, Kyu-Seok Hwang, Se Hwan Ahn, Seong Soon Kim, Yuji Son, Sung Bum Park, Won Hoon Jung, Dae-Seop Shin, Sung Hee Cho, Byeong Wook Choi, Pyeongkeun Kim, Yerim Heo, Minhee Kim, Jung Yoon Yang, Kyeong-Ryoon Lee, Hyang-Ae Lee, Jihun Kim, Hoon-Chul Kang, Ki Young Kim, Myung Ae Bae, Jin Hee Ahn

**Affiliations:** † Department of Chemistry, 65419 Gwangju Institute of Science and Technology, Gwangju 61005, Republic of Korea; ‡ Therapeutics & Biotechnology Division, 65680 Korea Research Institute of Chemical Technology, Daejeon 34114, Republic of Korea; § Department of Medical Chemistry and Pharmacology, 54679 University of Science & Technology, Daejeon 34113, Republic of Korea; ∥ Laboratory Animal Resource Center, Korea Research Institute of Bioscience and Biotechnology, Ochang 28116, Republic of Korea; ⊥ Center for Biomimetic Research, 443298 Korea Institute of Toxicology, Daejeon 34114, Republic of Korea; # Division of Pediatric Neurology, Department of Pediatrics, 37991 Severance Children’s Hospital, Yonsei University College of Medicine, Epilepsy Research Institute, Seoul 03722, Republic of Korea; ∇ JD Bioscience Inc, 208 Cheomdan-dwagiro, Buk-gu, Gwangju 61011, Republic of Korea

## Abstract

Severe myoclonic epilepsy of infancy (SMEI, Dravet syndrome), which is mainly caused by the *SCN1A* mutation, is a severe epileptic encephalopathy that manifests in infancy and leads to intractable seizures and developmental impairment. To discover new therapeutic chemotypes, we established a Nav1.1 (*scn1lab*) KO zebrafish model for chemical screening and identified novel 1,3,4-oxadiazol-2­(3*H*)-one derivatives. Among them, compound **20e** showed the most potent antiseizure efficacy in zebrafish behavioral assays and significantly reduced locomotion-related seizure parameters compared with repositioned drugs. In *SCN1A* ^+/–^ mice, **20e** reduced seizure severity, delayed onset, and suppressed hyperactivity. Notably, **20e** normalized pathological spike and burst activity in SMEI patient-derived iPSC neurons. Mechanistically, **20e** appears to elevate 5-HT levels via TPH2 upregulation. It demonstrated reasonable BBB penetration, favorable oral PK, and good safety without notable hERG inhibition, cytotoxicity, mutagenicity, or acute toxicity. Taken together, compound **20e** shows promise as a therapeutic agent for SMEI.

## Graphical abstract

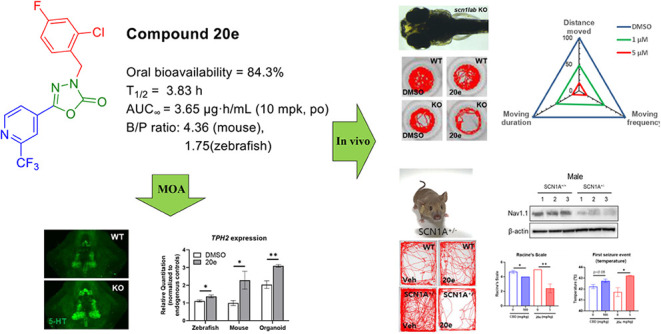

## Introduction

Severe myoclonic epilepsy of infancy (SMEI), also known as Dravet syndrome, is a rare disease that begins in infancy and proceeds with accumulating morbidity that significantly impacts individuals throughout their lifetime. Infant-onset seizures present as prolonged periods, generalized or unilateral, clonic or tonic-clonic, and fever symptoms, accompanied by intellectual and behavioral disorders.^1^

In the past decade, three antiseizure drugs (ASDs) were available as therapeutic agents in SMEI: fenfluramine (FFA), stiripentol (STP), and cannabidiol (CBD). These drugs were not originally developed for SMEI. But they have been repositioned in attempts to treat SMEI. In 2018, STP was approved in the US for the treatment of seizures associated with SMEI and proved to increase GABAergic transmission. However, STP has side effects including insomnia, ataxia, hypotonia, and dystonia. CBD is approved as an adjunctive treatment for seizures in SMEI and Lennox–Gastaut syndrome (LGS) in 2018 in the USA and in 2019 in the EU. CBD has side effects such as diarrhea, vomiting, fatigue, fever, sleepiness, and abnormal liver function. Recently approved as an adjuvant therapy for SMEI, FFA was first revealed as an antiseizure agent via modulating the serotonergic system.^2^ Despite being a withdrawn drug as an appetite suppressant, FFA was approved as a drug repositioning for SMEI treatment.^3^ However, FFA can cause side effects, such as sedation, insomnia, and weight loss. Additionally, Brenot and colleagues suggest that fenfluramine derivatives are strongly suspected etiological agents of primary pulmonary hypertension in female cases.^4^ These limitations underscore the need for novel therapeutic approaches with improved efficacy and safety profiles.

More than 80% of SMEI patients exhibit mutations in the sodium voltage-gated channel alpha subunit (*SCN1A*), which encodes the type I voltage-gated channel (Nav1.1) alpha subunit.^5^ The voltage-gated sodium channel plays an important role in the initiation and propagation of the action potential of neurons in the brain.^6^ Baraban et al. identified a zebrafish with a mutation in *scn1lab* (sodium channel, voltage-gated, type 1 like, alpha b), *didy* ^*s552*^, and demonstrated the presence of spontaneous seizure phenotypes in *scn1lab* mutants using electrophysiological recording and behavioral analysis. They used this model for SMEI and discovered clemizole, which is the repositioned molecule developed as a histamine H1 receptor antagonist that inhibits convulsive behaviors and electrographic seizures.^7^ It prompted us to identify a novel chemotype using a new phenotype-based screening. We established a new *scn1lab* knockout (KO), *kri111* allele, by inserting four nucleotides in exon 2 using the CRISPR/Cas9 method. This established mutant was used to identify novel antiseizure agents for SMEI. We performed a phenotype-based screening of 6,625 small molecules from the Korea Chemical Bank library. Oxadiazolone (**1**) was identified as a novel hit scaffold and was optimized to identify candidates for SMEI treatment. Herein, we report the synthesis and biological evaluation of novel oxadiazolone derivatives for SMEI.

## Results

In this study, we generated a new *scn1lab* KO zebrafish using the CRISPR/Cas9 method to evaluate the antiseizure efficacy of synthesized compounds. A 4-bp insertion was created in exon 2 of the *scn1lab* genome, generating a premature stop and truncated Scn1lab protein (referred to as the *kri111* allele) (Figure S1a). *scn1lab* KO (homozygous *kri111* allele) larvae showed similar morphological phenotypes to those observed in *didy* ^*s552*^ including hyperpigmentation and an uninflated swim bladder (Figure S1b). Based on the three stages of seizure phases in zebrafish larvae: hyperactivity (stage 1), rapid “whirlpool-like” behavior (stage 2), and loss-of-posture (stage 3), we newly defined seizure-like movements to quantify high-speed movement at a speed above 80 mm/s.^7,8^ Since hyperactivity showed irregularities in *scn1lab* KO larvae, it is difficult to quantify seizure-like movement patterns through normal movements. However, only calculating high-speed movements above 80 mm/s can quantify hyperactivity. Indeed, the differences in normal movement between WT and *scn1lab* KO varied, whereas *scn1lab* KO movements are more increased than those of WT at a speed above 80 mm/s (Figure S1c).

We tried to identify a novel molecule that significantly reduced seizure-like movements compared with control groups when administered to *scn1lab* KO larvae and showed no behavioral change when administered to WT larvae. As a result of compound screening using the chemical library from the Korea Chemical Bank (comprising 6,625 small molecules), compound **1** was identified as a hit. It significantly decreased seizure-like movements in the *scn1lab* KO larvae compared to those in the control group (55.3%, 50.0%, 54.6% in distance moved, movement frequency, and movement duration, Table 1). Whereas compound **1** showed no significant difference in WT larvae compared to the control group (103%, 111%, 105% in distance moved, movement frequency, and movement duration, Table 1). We optimized the hit compound **1** by systematic modification of structural parts A, B, and C as shown in Figure 1.

**Figure 1:**
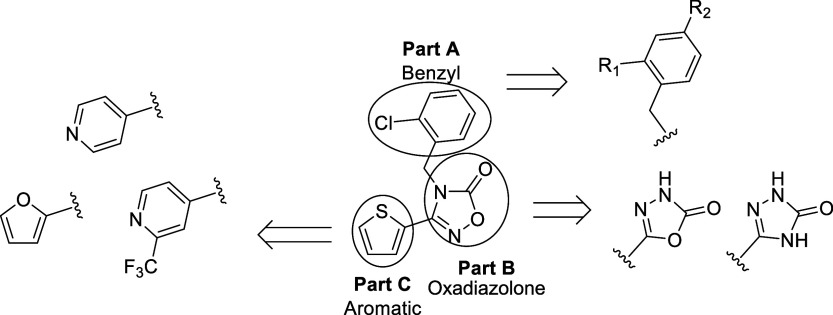
Structure and modification of compound (**1**).

The synthetic route for 1,2,4-oxadiazolone derivatives is outlined in Scheme 1. Commercially available 2-cyanothiophene (**2**) was first coupled with hydroxylamine to give **3**, followed by cyclization to produce 1,2,4-oxadiazolone **4**. The phenyl-substituted 1,2,4-oxadiazolone (**8**) was obtained by coupling **4** with **7**, which was prepared from 2-chloroiodobenzene (**5**). Compound **4** was alkylated with benzyl and phenethyl halides (**9a** and **10b**) to afford **1** and **10**, respectively. Diverse benzyl derivatives (**12a**–**f**) were synthesized via the reaction of 1,2,4-oxadiazolone **4** with benzyl bromide (**11a**) and substituted benzyl bromides (**11b**–**f**).

**Scheme 1:**
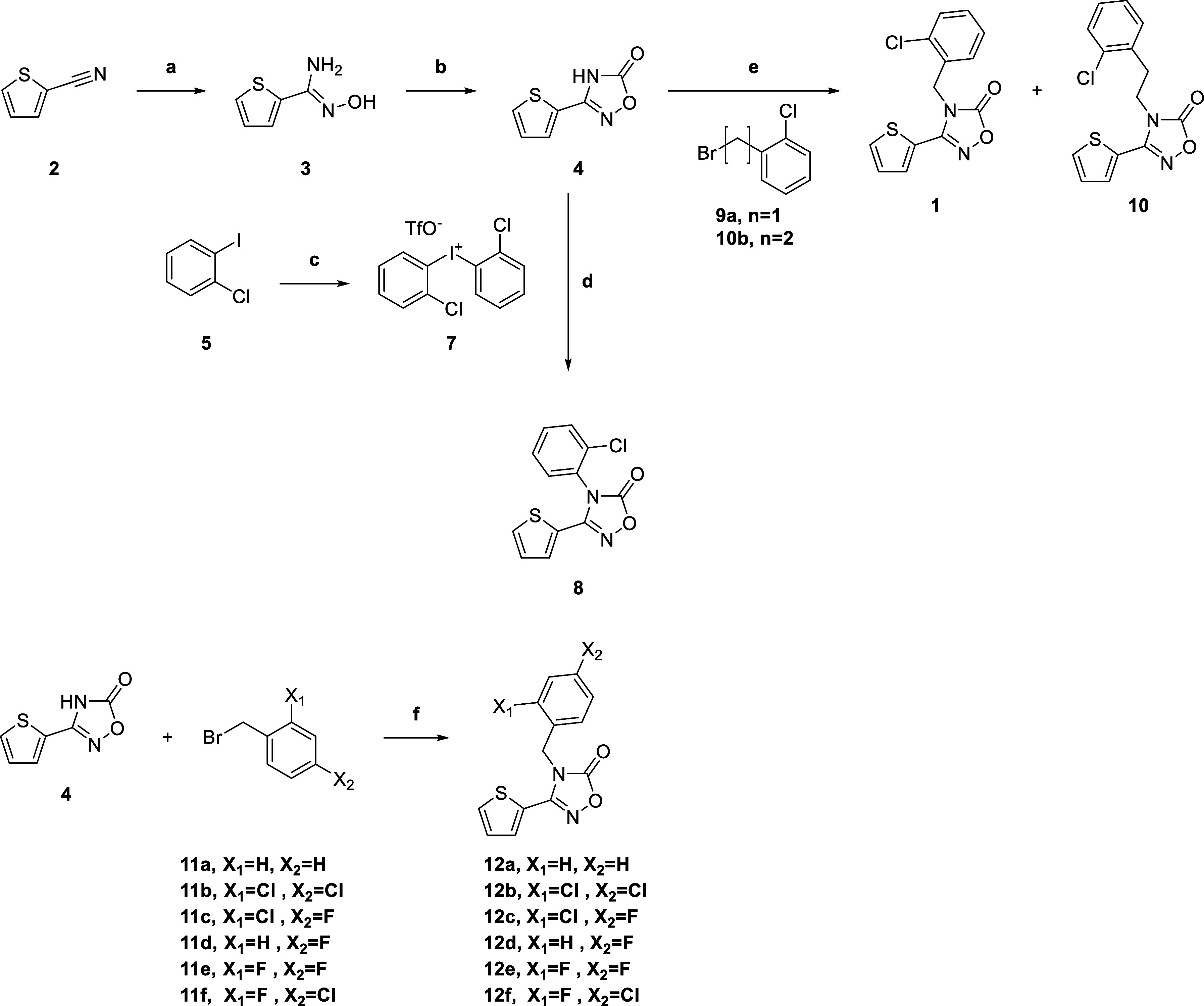
Synthesis of Compounds **1**, **7**, **10** ^a^

a Reagents and conditions: (a) NH_2_OH·HCl, TEA, EtOH, reflux, 94%, (b) Triphosgene, DIPEA, THF, reflux, 72%, (c) mCPBA, BF_3_·Et_2_O, (2-Chlorophenyl)­boronic acid **6**, TfOH, 62%, (d) CuI, TEA, DCE, 60 °C, 71%. (e) NaH, DMF, 60 °C, 73–74%. (f) NaH, DMF, 60 °C, 74–77%.

The synthetic pathway for 1,2,4-Triazolone and 1,3,4-oxadiazolone derivatives is depicted in Scheme 2. Methyl thiophene-2-carboxylate (**13a**) was treated with hydrazine to give hydrazide (**14a**), which underwent cyclization with BrCN to give oxadiazole (**15**). This oxadiazole intermediate was converted into triazole (**16**) under KOH/ethanol condition, followed by benzylation and hydrolysis to give the desired triazolone (**18**). For the synthesis of 1,3,4-oxadiazolone derivatives, aryl esters (**13a**–**e**) were converted into acylhydrazides (**14a**–**e**), which were cyclized using triphosgene and subsequently benzylated with substituted benzyl bromide to yield the final 1,3,4-oxadiazolone derivatives (**20a**–**e**).

**Scheme 2:**
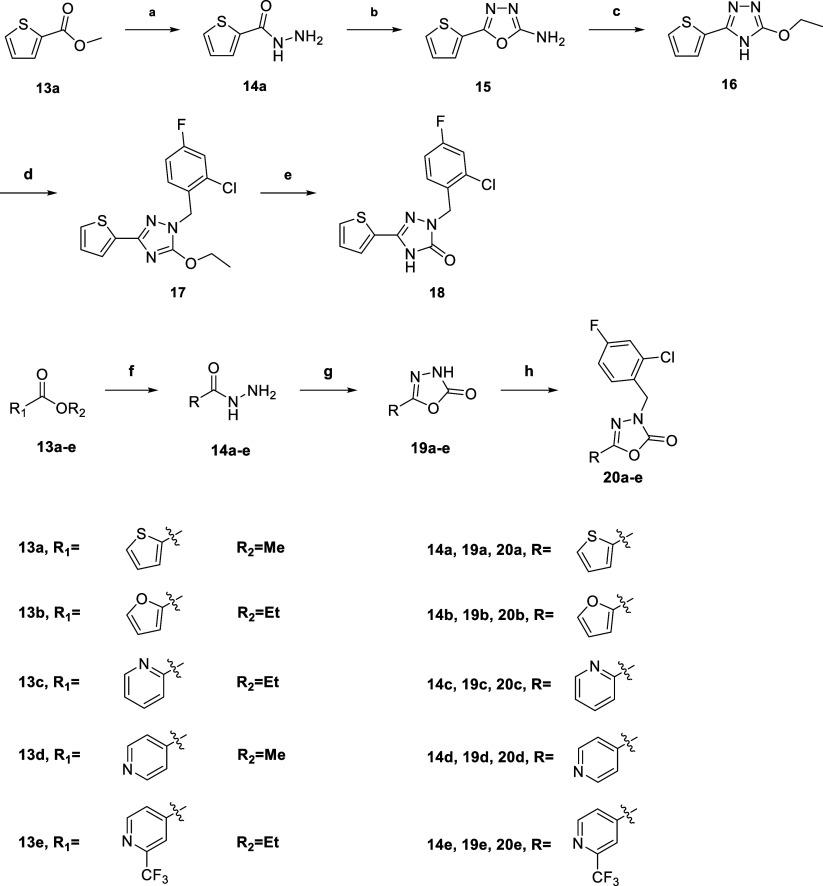
Synthesis of Compounds **18**, **20a**-–**e** ^a^

a Reagents and conditions: (a) NH_2_NH_2_·H_2_O, EtOH, reflux, 72%, (b) BrCN, NaHCO_3_, DMF, H_2_O, rt, 16 h, 51%, (c) KOH, EtOH, reflux, 16 h, 77%, (d) NaH, DMF, 60 °C, 69%, (e) HCl, EtOH, H_2_O, reflux, 62%. (f) NH_2_NH_2_·H_2_O, EtOH, reflux, 72–98%, (g) Triphosgene, DIPEA, THF, reflux, 66–75%, (h) NaH, DMF, 60 °C, 57–66%.

To optimize the hit, first, the chain length between 2-chlorobenzene and 1,3,4-oxadiazolone was changed, and the results are summarized in Table 1. Unlike the hit compound **1**, 2-chlorophenyl **8**, and 2-chlorophenethyl **10** derivatives showed no significant antiseizure efficacy. Therefore, we optimized the benzyl moiety of **1**. The unsubstituted benzyl derivative (**12a**) exhibited a weaker efficacy (85.8%) compared to compound **1** (53.3%). The 2,4-Dichloro derivative **12b** showed improved efficacy, with 65.3%. Further, the 2-chloro-4-fluorobenzyl derivative (**12c**) showed further enhancement (40.4%) in *scn1lab* KO larvae compared to compound **1**. To investigate the effect of chlorine at position 2 of the benzyl group, two compounds were designed and synthesized by substituting chlorine with hydrogen (**12d**) and fluorine (**12e**), which showed no efficacy in *scn1lab* KO larvae. The derivative with 4-chloro-2-fluorobenzyl (**12f**) was also examined for its activity but exhibited a weaker activity (63.0%) than that of **12c**. Based on these results, the 2-chloro-4-fluorobenzyl moiety (**12c**) was identified as the most suitable structure for Part A, and subsequent optimization focused on modifications of the five-membered 1,3,4-oxadiazolone ring.

**Table 1:**
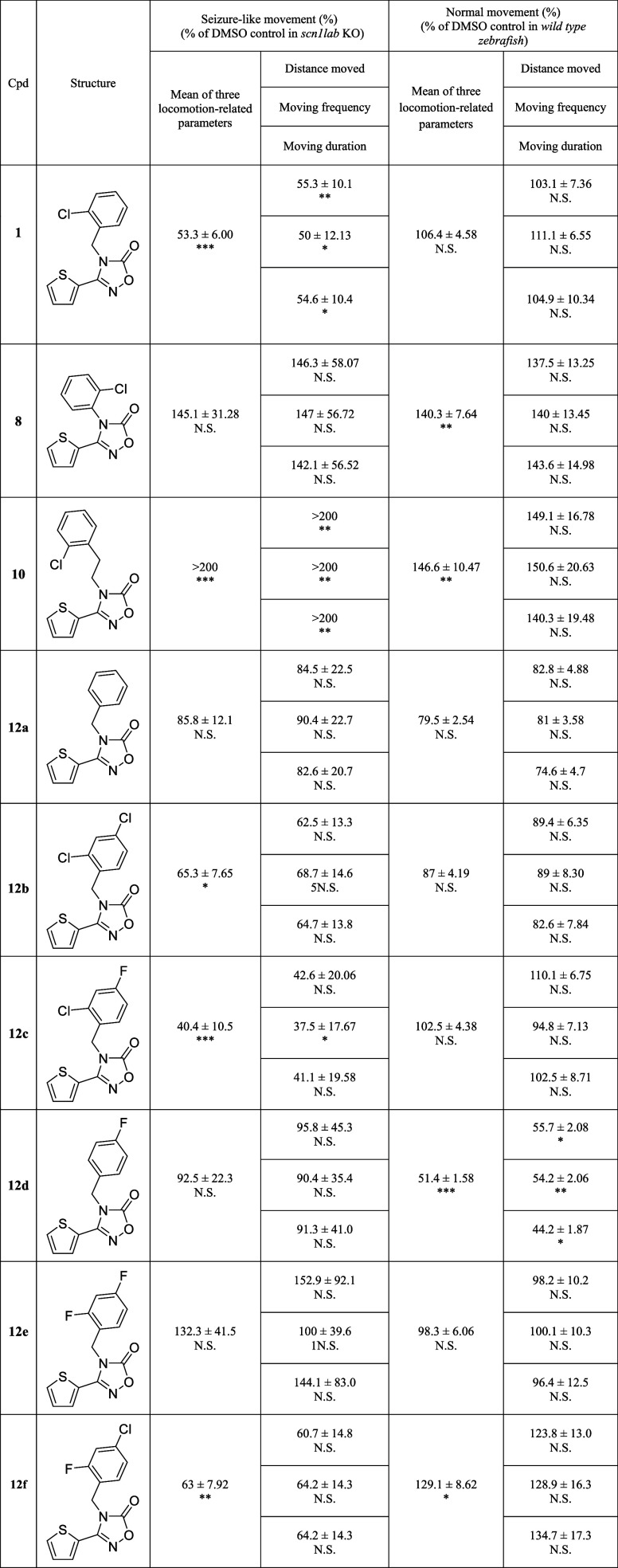
SAR Exploration of Compounds **1**, **7**, **10**, 12a–f^a^

a *p* value: N.S., Not significant; *p ≤ 0.05; **p ≤ 0.01; ***p ≤ 0.001.

The 1,2,4-oxadiazol-5­(2*H*)-one structure (Part B) was modified, and the results are summarized in Table 2. The derivative with the 1,3,4-oxadiazolone moiety (**20a**) exhibited an enhanced antiseizure effect (19.7%) than **12c**, whereas triazolone **18** exhibited no antiseizure effects (98.7%). These results indicated that among the five-membered heterocyclic core moieties including oxadiazolones (Part B), the 1,3,4-oxadiazolone scaffold was the most suitable structure for antiseizure activity, and subsequent optimization focused on modifying the aromatic ring.

**Table 2:**
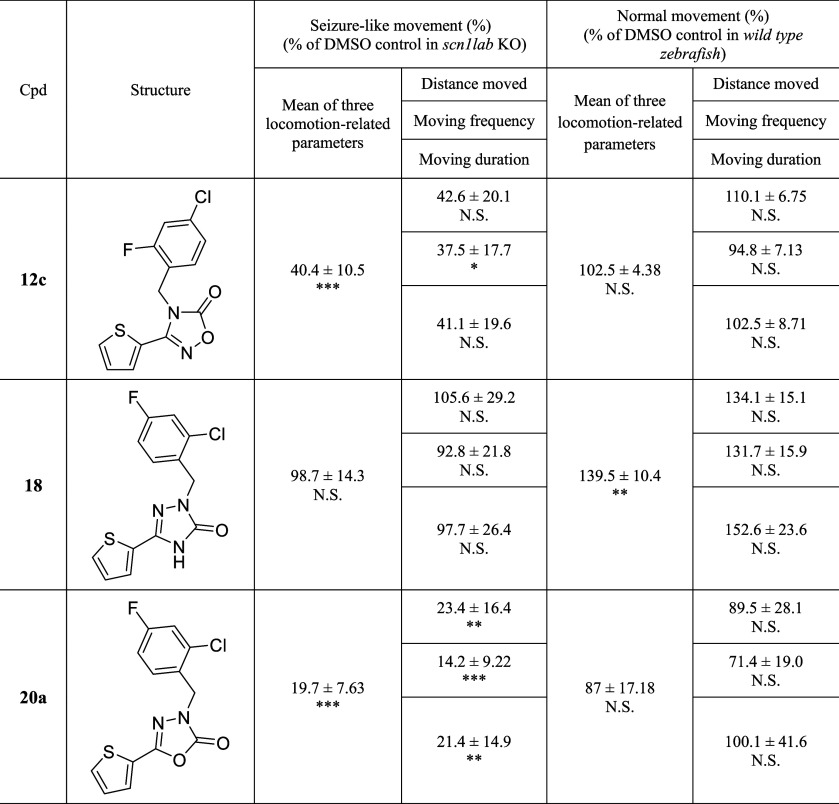
SAR Exploration of Compounds **18** and 20a^a^

a *p* value: N.S., Not significant; *p ≤ 0.05; **p ≤ 0.01; ***p ≤ 0.001.

The aromatic moiety (Part C) was optimized by the introduction of diverse aromatic groups and the results are summarized in Table 3. We found compound **20b**, which replaced the thiophene scaffold with the same 5-membered furan, exhibited dramatically improved efficacy (2.4%); however, it showed a behavioral change in WT larvae (46.5%). Good antiseizure efficacies were observed in 6-membered pyridine derivatives such as the Pyridin-2-yl compound (**20c**) and the pyridin-4-yl compound (**20d**). Further, compound **20e,** a 3-trifluoromethylpyridine-4-yl derivative, showed good antiseizure efficacy (19.5%) without significant differences in WT larvae. Therefore, we selected **20a** and **20e** for further evaluation. However, compound **20a** showed poor stability in the liver microsomal assay, so compound **20e** was chosen for further development.

**Table 3:**
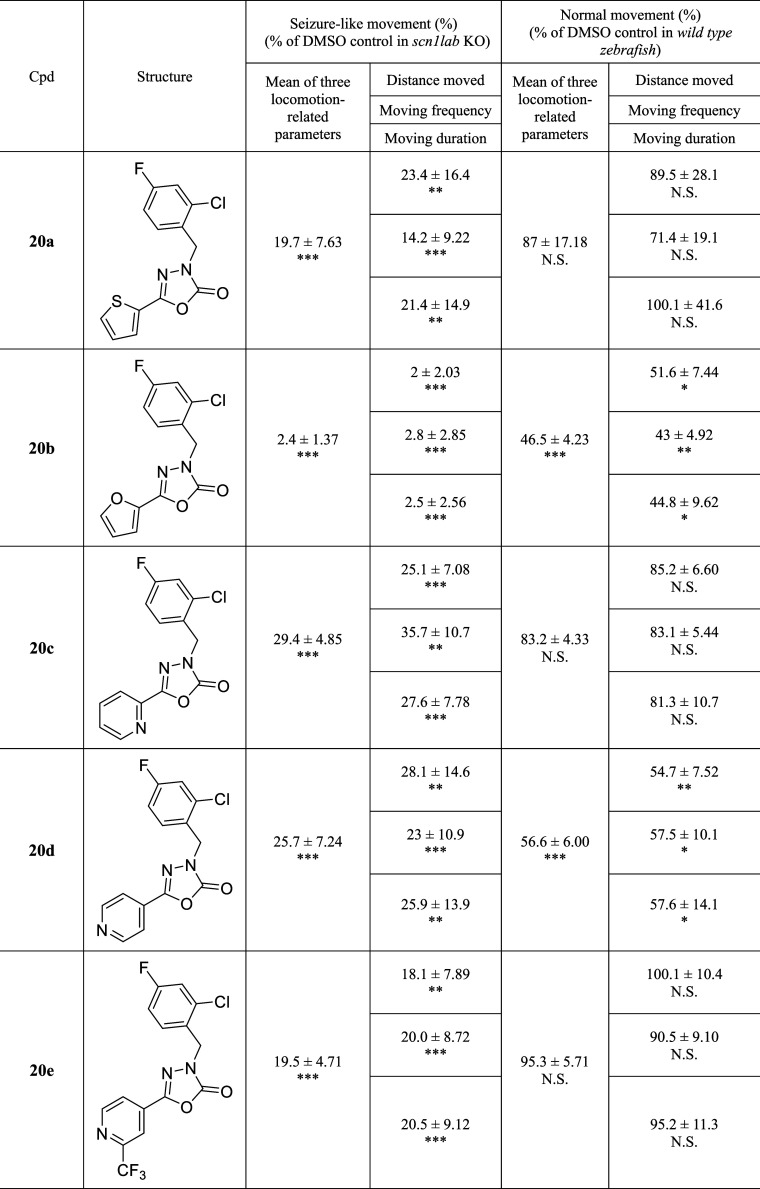
SAR Exploration of Compounds 20a–e^a^

a *p* value: N.S., Not significant; *p ≤ 0.05; **p ≤ 0.01; ***p ≤ 0.001.

Compound **20e** was evaluated for its its blood-brain barrier (BBB), DMPK, and toxicity, and the results are presented in Table 4. The BBB serves as a gatekeeper of the central nervous system (CNS),^9^ BBB penetration is essential for the efficacy of antiseizure medication in various biological models, including mouse, zebrafish, and human BBB chip systems. Compound **20e** exhibited BBB-permeable properties; the brain-to-plasma (B/P) ratios were 4.36 ± 0.89, 1.75 ± 0.57, or 0.43 ± 0.12 in the mouse model, the adult zebrafish model, and the human BBB system, respectively (Table 4a). Compound **20e** has the good pharmacokinetic properties for an oral drug, with a reasonable AUC_0–24h_ (3.60 ± 1.34 μg·h/mL for the I.V. route and 3.83 ± 0.21 μg·h/mL for the P.O. route), rapid absorption (*T*_max_ = 1.17 ± 0.76 h), and a moderate half-life with 3.83 ± 0.21 h following P.O. administration at a dose of 10 mg/kg in the mouse model. The bioavailability (*F*) of compound **20e** was estimated to be 84.3% after P.O. administration (Table 4b). In liver microsomal stability tests, compound **20e** demonstrated reasonable stability during phase I metabolic reactions (Table 4c). The percentages of compound remaining after 30 min of incubation were 69.2% in rat, 75.6% in dog, 79.0% in monkey, and 82.4% in human liver microsomes. Compound **20e** exhibited excellent stability in plasma from various animals, including mice, rats, and humans. After a 4 h incubation period, the stability was greater than 99.9% in mouse plasma, 89.8% in rat plasma, and 89.9% in human plasma. Additionally, after a 4-h equilibrium state, the plasma protein binding rates of compound **20e** were 98.3% in mouse, 98.0% in rat, and 99.2% in human plasma, respectively. Various toxicity studies (cytotoxicity, acute toxicity test, hERG, and AMES) were performed in *in vitro* and *in vivo* systems. To determine the cytotoxicity of compound **20e** against mammalian cell lines, the cell viability was investigated by WST analysis. WST analysis was performed on mammalian cells (VERO, HFL-1, L929, NIH3T3, CHO-K1) after 24 h of treatment with varying concentrations of compound **20e** (0.01, 0.1, 1, 10, and 100 μM). Compound **20e** did not affect the normal cell viability. The potential of compound **20e** to inhibit hERG was evaluated using the hERG FP (fluorescence polarization) assay. Compound **20e** showed a low possibility of cardiac toxicity induced by inhibition of hERG channel activity with <1% inhibition at 10 μM.^10^ A mini-Ames test was conducted with **20e** using two strains of *Salmonella typhimurium* (TA98 and TA100) to assess its mutagenic potential. The results indicated that compound **20e** exhibited no mutagenicity with or without the S9 fraction. Moreover, in an acute oral toxicity study, compound **20e** exhibited no toxicological effects on body weight or organ morphology after the administration of 250 mg/kg to both male and female mice.

**Table 4:** Biological Data of Compound **20e**

			Matrix		
Test compound	Model	Drug administration	Plasma conc.(ng/mL)	Brain conc.(ng/g)	B/P ratio
a)^a^					
**20e**	Mouse	P.O. (10 mg/kg)	157 ± 115	706 ± 619	4.36 ± 0.89
	Zebrafish	P.O. (10 mg/kg)	951 ± 258	1699 ± 779	1.75 ± 0.57
	Human BBB-chip	10 μM	366 ± 10	157 ± 10	0.43 ± 0.07

Parameters	I.V., 2 mg/kg	P.O., 10 mg/kg
b)^b^		
*T*_max_ (h)	NA	1.17 ± 0.76
*C*_max_ (μg/mL)	NA	0.47 ± 0.21
*T*_1/2_ (h)	3.74 ± 1.37	3.83 ± 0.21
AUC_t_ (μg·h/mL)	0.85 ± 0.16	3.60 ± 1.34
AUC_∞_ (μg·h/mL)	1.06 ± 0.18	3.65 ± 1.35
CL (L/h/kg)	1.91 ± 0.30	NA
*V*_ss_ (L/kg)	8.36 ± 4.12	NA
***F*** **(%)**	**NA**	84.3

Assay	Results
	c)^c^
Liver microsomal stability (% remaining after 30 min incubation)	69.2 (rat), 75.6 (dog), 79.0 (monkey), 82.4 (human)
Stability in plasma (% remaining after 4 h incubation)	>99.9 (mouse), 89.8 (rat), 89.9 (human)
Plasma protein binding rate (%)	98.3 (mouse), 98.0 (rat), 99.2 (human)
Cytotoxicity (IC_50_)	>100 μM in various mammalian cell lines (VERO, HFL-1, L929, NIH 3T3, CHO-K1)
Mini-Ames	No mutagenicity
hERG ligand binding	<1% inhibition, 17.9% inhibition at 10, 100 μM concentrations
CYP inhibition IC_50_	1A2 (17.6 μM), 2C9 (23.1 μM), 2C19 (1.3 μM), 2D6 (16.7 μM), 3A4 (>100 μM)

a Concentrations (ng/mL or ng/g) and brain-to-plasma (B/P) ratios of compound **20e** in mouse and zebrafish models. Animals: 12-month-old adult zebrafish (*n* = 5), dose: P.O. 10 mg/kg, dosing vehicle: DMSO:DW:PEG400 = 5:40:55, time points: 0.5 h, drug analysis: UPLC combined with LC-MS/MS (Waters Xevo TQ-S LC-MS/MS), LC condition: BEH C18 column (50 × 2.1 mm, 1.7 μm, Waters), mobile phase: 0.1% FA in water and 0.1% FA in acetonitrile (gradient elution) animals: 7-week-old ICR mice (*n* = 3), dose: P.O. 10 mg/kg, dosing vehicle: DMSO:DW:PEG400 = 5:40:55, time points: 0.5 h, drug analysis: UPLC combined with LC-MS/MS (Waters Xevo TQ-S LC-MS/MS), LC condition: BEH C18 column (50 × 2.1 mm, 1.7 μm, Waters), mobile phase: 0.1% FA in water and 0.1% FA in acetonitrile (Gradient elution).

b Pharmacokinetic parameters of **20e** in male mice. *C*_max_, maximal drug concentration; *T*_max_, time to reach *C*_max_; *T*_1/2_, half-life; AUC, area under the curve; CL, clearance; *V*_ss_, volume of distribution at steady state; *F*, absolute bioavailability. NA, not applicable; ND, not detected; NC, not calculated.

c ADME/T properties of compound **20e** *in vitro*.

Compound **20e** treatment in *scn1lab* KO larvae effectively decreased seizure-like movements in a dose-dependent manner (Figures S1d,e and S2). We compared the antiseizure efficacy of **20e** with FFA and CBD on *scn1lab* KO larvae. FFA treatment significantly reduced seizure-like movements in *scn1lab* KO larvae by 27.96% at 250 μM and 9.44% at 500 μM but also decreased normal movements in WT larvae. CBD treatment decreased seizure-like movements by 55.58% at 5 μM and 11.51% at 10 μM without affecting normal movements in WT larvae. Compound **20e** treatment effectively reduced seizure-like movements by 41.58% at 2.5 μM and 16.3% at 5 μM without affecting normal movements in WT larvae. Compound **20e** demonstrated the most effective reduction in seizure-like movements in *scn1lab* KO larvae. Additionally, unlike FFA, compound **20e** did not affect normal movements in WT larvae, making it a particularly promising candidate for further study (Figure 2). **20e** treatment also exhibited excellent antiseizure efficacy in the pentylenetetrazole (PTZ)-induced seizure zebrafish model (Figure S3). Electroencephalogram data showed that seizure-like events increased in KO larvae compared with the WT group. However, **20e** treatment reduced both the number and duration of ictal-like events in the KO zebrafish larvae (Figure S1f). Prolonged or recurrent seizures in epilepsy patients, including those with SMEI, have been reported to cause cognitive impairment.^11^ Because zebrafish have innate color discrimination capability, the cognitive ability of zebrafish larvae can also be evaluated through color preference testing.^12^ WT or heterozygous sibling larvae tended to prefer blue over yellow, whereas *scn1lab* KO larvae showed the opposite tendency. However, compound **20e** treatment in *scn1lab* KO larvae tended to restore the preference for blue (Figure S1g).

**Figure 2:**
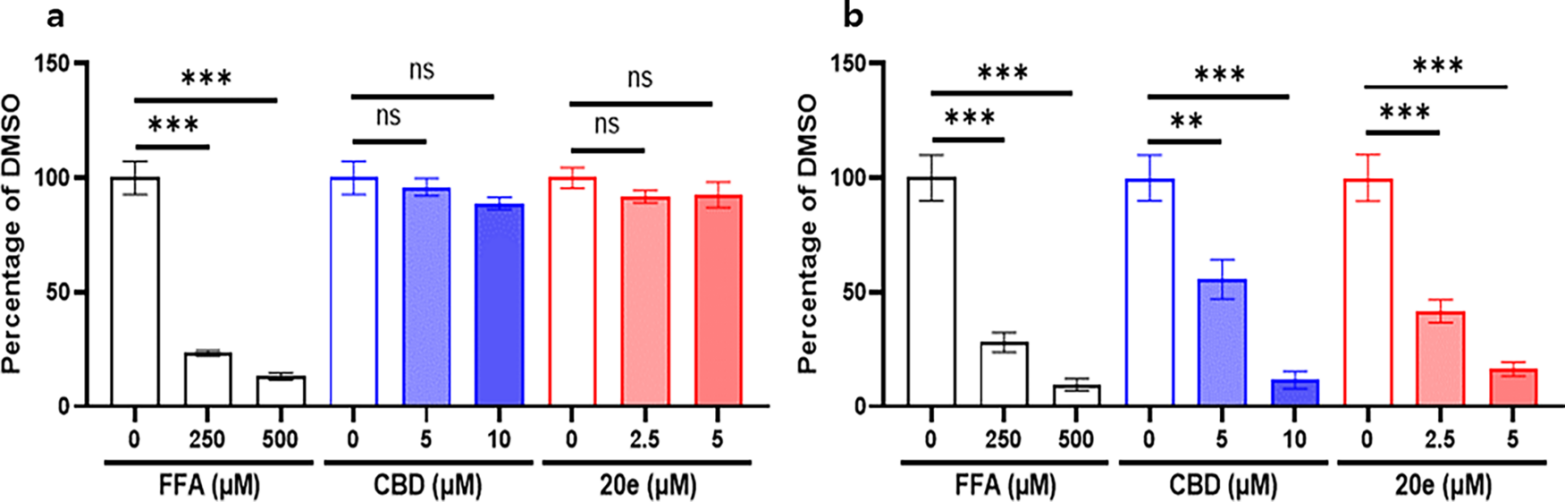
Antiseizure effects of compound **20e** compared to FFA and CBD in *scn1lab* KO zebrafish larvae. A, B Quantification of normal movements in WT larvae and seizure-like movements in *scn1lab* KO larvae following treatment with FFA, CBD, or compound **20e**. FFA reduced seizure-like movements at 250 μM (27.96%) and 500 μM (9.44%) but also decreased normal locomotion in WT larvae. CBD reduced seizure-like movements at 5 μM (55.58%) and 10 μM (11.51%) without affecting the WT movement. Compound **20e** reduced seizure-like movements at 2.5 μM (41.58%) and 5 μM (16.3%) without altering normal locomotion in WT larvae. Compound **20e** showed the most favorable efficacy–safety profile among the tested compounds.

To evaluate the in vivo efficacy, heat-induced seizure modeling was performed in *SCN1A* ^+/–^ mice. Generalized tonic-clonic seizures (GTCS) were not observed in animals treated with CBD or compound **20e** (data not shown). Treatment with both CBD and compound **20e** significantly mitigated seizure severity, assessed by the Racine scale score (Figure 3a). CBD administration reduced the mean score from 4.67 ± 0.21 (vehicle) to 4.00 ± 0.00 (a reduction of 0.67 points). Compound **20e** demonstrated superior efficacy, reducing the score from 5.00 ± 0.00 (Vehicle) to 2.40 ± 0.60, achieving a substantial 2.60-point reduction and a 52% decrease in severity (Figure 3a). Furthermore, both the first seizure threshold temperature and the latency to the first seizure were significantly increased in the **20e**-treated *SCN1A* ^+/–^ group (Figure 3b,c), indicating enhanced resistance to hyperthermia-induced and spontaneous seizures. In the open field test, *SCN1A* ^+/–^ mice exhibited significantly greater basal locomotor activity, including increased distance traveled and average speed, compared to their WT controls, consistent with previous findings.^13^ The effect of CBD and **20e** on hyperactivity track plots visually confirmed a reduction in the hyperactivity of mutant mice following treatment with either CBD (100 mg/kg) or **20e** (5 mg/kg) (Figure 3d). Quantitatively, both CBD and **20e** treatments significantly reduced the total distance traveled (Figure 3e) and average speed (Figure 3f) in *SCN1A* ^+/–^ mice. Importantly, neither CBD nor **20e** treatment affected the locomotor behavior of the WT control mice. These data collectively suggest that treatment with CBD or **20e** successfully ameliorated the behavioral phenotypes associated with SCN1A haploinsufficiency by reducing the observed hyperactivity.

**Figure 3:**
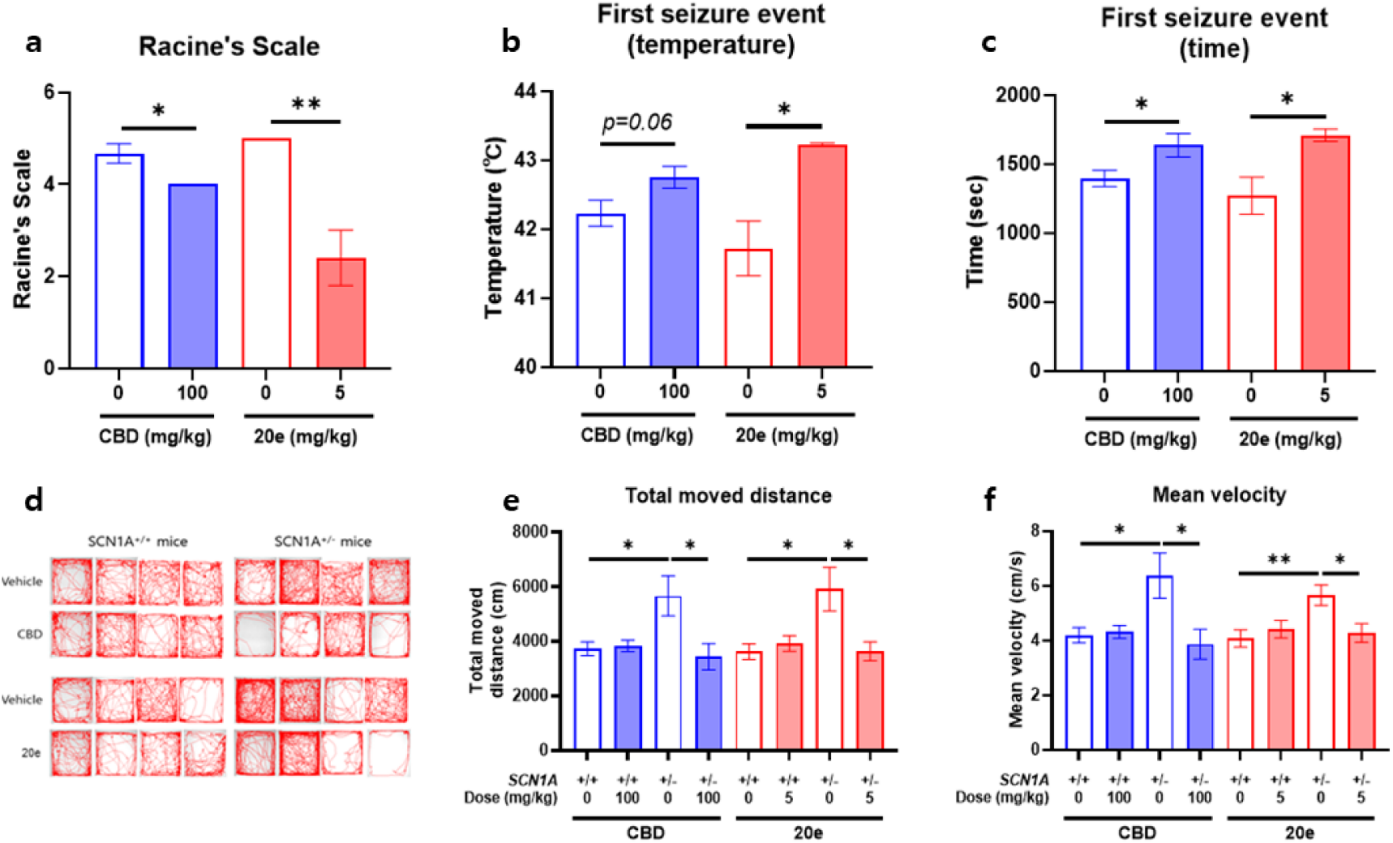
Compound **20e** reduces seizure susceptibility and hyperactivity in *SCN1A* ^+/–^ mice. **a** Racine scale scores in heat-induced seizure assays. Treatment with CBD (100 mg/kg) or compound **20e** (5 mg/kg) significantly reduced seizure severity, with **20e** exhibiting a 52% reduction relative to vehicle controls. **b, c** Compound **20e** significantly increased latency to first seizure and seizure threshold temperature, indicating enhanced resistance to hyperthermia-induced seizures. **d–f** Open field test showing hyperlocomotion in *SCN1A* ^+/–^ mice compared to WT. Both CBD and **20e** treatments reduced the total distance traveled and average speed in *SCN1A* ^+/–^ mice without affecting WT behavior. Representative track plots illustrate reduced hyperactivity in treated mutant mice.

To investigate the functional effects of compound **20e**, a cellular model of epilepsy was created using patient-derived iPSCs. A six-week differentiation period was required to achieve a functional, epilepsy-like phenotype. During this period, the iPSC-derived neurons showed a noticeable increase in the firing frequency over time. Spike and burst activity gradually rose, and network burst activity first appeared at week 5, becoming more pronounced by week 6. These developmental changes confirmed a hyperexcitable neuronal network suitable for drug evaluation (Figure 4a–d).

**Figure 4:**
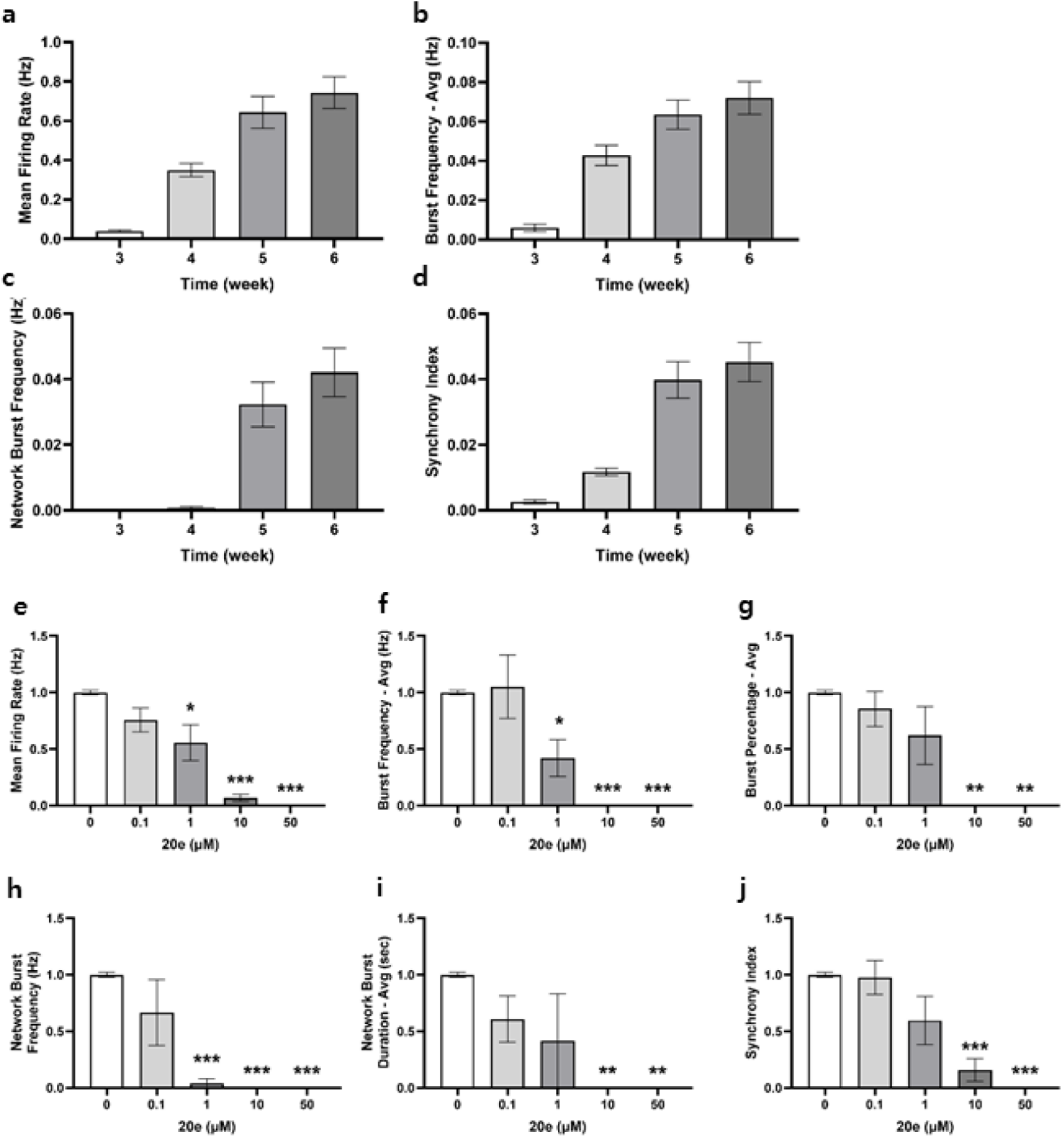
Electrophysiological characterization of compound **20e** using microelectrode array. **a−d** Temporal changes in neuronal activity metrics during differentiation from weeks 3 to 6: **a** mean firing rate, **b** burst frequency, **c** network burst frequency, and **d** synchrony index. Data were expressed as mean ± SEM (*n* = 48). **e–j** Efficacy of compound **20e** on electrophysiological metrics: **e** mean firing rate, **f** burst frequency, **g** burst percentage, **h** network burst frequency, **i** network burst duration, and **j** synchrony index.

The effects of compound **20e** on the electrophysiological characteristics of the epileptic neuronal network were assessed in a dose-dependent manner. Concentrations up to 100 nM showed no significant change in the mean firing rate. However, a significant decrease was observed starting at 1 μM (Figure 4e). A similar pattern was seen for burst activity, with the burst index and burst percentage both showing a significant reduction at concentrations of 1 μM and above (Figure 4f,g). The drug’s impact on synchronized network activity was also evaluated. While no significant changes in network burst frequency or duration were seen at concentrations below 1 μM, both metrics were significantly and dose-dependently reduced at 10 and 50 μM (Figure 4h,i). Furthermore, the synchronization index—a key measure of collective network function—showed a significant decrease at concentrations exceeding 1 μM (Figure 4j). Compound **20e** effectively suppresses both individual neuronal firing and coordinated network-level activity in this patient-derived epilepsy model, with its inhibitory effects becoming significant at concentrations of 1 μM and above.

We investigated the mode of action of **20e**. Neurotransmitter profiling has emerged^14^ as a critical tool in elucidating CNS functions and uncovering related behavioral profiles across various animal models, including zebrafish. Mass spectrometry-based targeted analysis was used to determine the endogenous concentrations of neurotransmitters in zebrafish larvae. This study explored a broad spectrum of brain neurochemistry, including histaminergic, cholinergic, dopaminergic, serotonergic, and GABAergic systems, each closely associated with behavioral profiles in these models. Using this technology, significant changes induced by compound **20e** were observed in the endogenous levels of neurotransmitters such as dopamine (DA), norepinephrine (NE), serotonin (5-HT), gamma-aminobutyric acid (GABA), glutamate (GLU), and glutamine (GLN) in zebrafish larvae. Notably, *scn1lab* KO zebrafish showed a decreased trend in 5-HT levels compared with WT counterparts. However, compound **20e** exhibited significant neurological effects by increasing the levels of 5-HT and GABA in both WT and *scn1lab* KO zebrafish larvae as compared to the control group (Figure 5a). KO zebrafish also showed reduced 5-HT immunoreactivity in the hypothalamus; however, 5-HT levels were effectively restored following treatment with **20e** (Figure 5b,c). These neurotransmitters exhibit sedative and antiseizure effects, potentially alleviating symptoms associated with SMEI.

**Figure 5:**
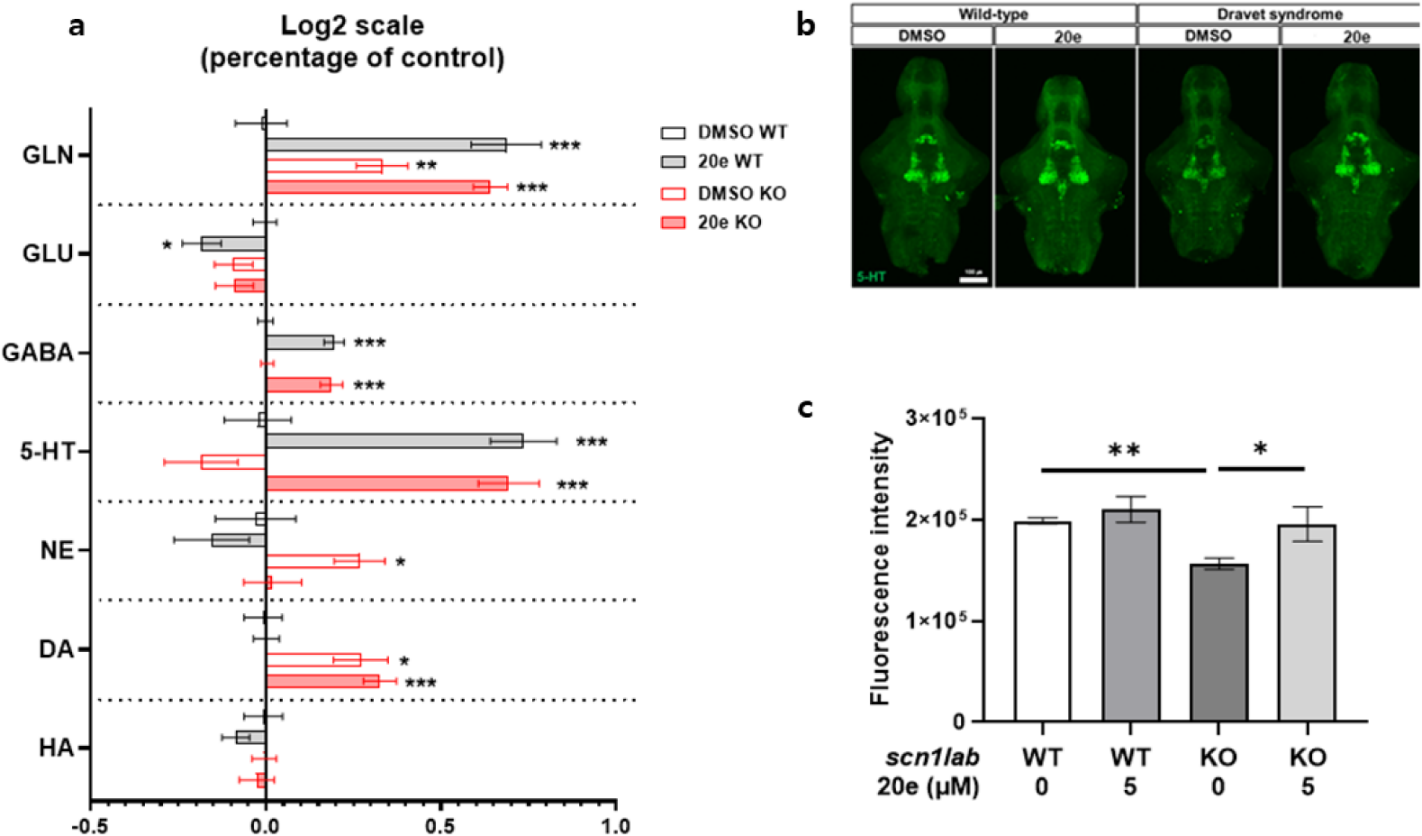
Compound **20e** modulates neurotransmitter levels in zebrafish larvae. **a** Quantification of endogenous neurotransmitters (DA, NE, 5-HT, GABA, GLU, and GLN) in WT and *scn1lab* KO zebrafish larvae following treatment with compound **20e**. Treatment significantly increased 5-HT and GABA levels in both genotypes compared to control. KO larvae showed a reduction in basal 5-HT levels relative to WT. **b, c** Representative images and quantification of 5-HT immunoreactivity in the hypothalamus of *scn1lab* KO larvae. Reduced 5-HT immunoreactivity in KO was rescued by compound **20e** treatment.

Tryptophan hydroxylase 2 (TPH2) is an enzyme variant of tryptophan hydroxylase that is found in vertebrates, and TPH2 plays an important role in 5-HT synthesis in the brain. In humans, TPH2 is predominantly expressed in the serotonergic neurons within the brain, with the highest levels of expression observed in the raphe nucleus located in the midbrain.^15^ In zebrafish, the genome contains three *tph* gene paralogs, but among these, only *tph2* is expressed specifically in the raphe nucleus.^16^ Compound **20e** treatment increased the endogenous 5-HT level in both WT and *scn1lab* KO larvae. Based on this result, quantitative RT-PCR was performed to analyze the expression of *tph2*. As a result, compound **20e** treatment in *scn1lab* KO larvae increases the expression of *tph2*, whereas it is not altered in WT larvae (Figure 6a). Furthermore, *TPH2* expression measured by real-time PCR showed no significant difference between the wild type and mutant type in both mouse and cerebral organoid models. However, upon **20e** treatment, only the *SCN1A* ^+/–^ group exhibited a significant upregulation of *TPH2* expression at mRNA levels (Figure 6b,c). This effect was consistent across in vivo and in vitro systems. These results suggest a mutation-specific sensitivity of the serotonergic pathway to pharmacological modulation.

**Figure 6:**
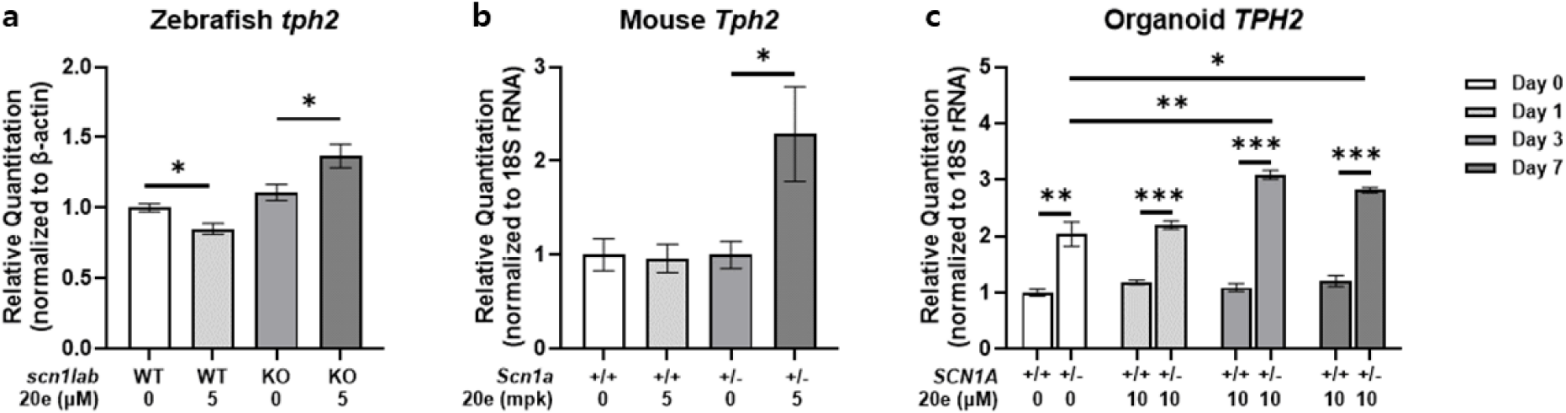
Compound **20e** selectively upregulates*TPH2* expression in*SCN1A* **-**deficient models. **a**, Quantitative PCR analysis of *tph2* expression in WT and *scn1lab* KO zebrafish larvae following **20e** treatment. **20e** significantly increased *tph2* expression in KO larvae. **b, c** The *tph2* mRNA levels in mouse brain and cerebral organoids showed no baseline difference between WT and *SCN1A* ^+/–^ groups. Upon **20e** treatment, *tph2* expression was significantly upregulated only in *SCN1A* ^+/–^ models. These findings indicate a mutant-dependent serotonergic response to **20e** across both *in vivo* and in vitro systems.

## Discussion

In this study, we aimed to identify a novel drug candidate for SMEI. We successfully established *scn1lab* KO zebrafish through a 4-bp insertion via the CRISPR/Cas9 method. Unlike the missense mutant *didy* ^*s552*^ zebrafish, *kri111* zebrafish produces the Scn1lab protein, which is truncated by a premature stop. However, the morphological and behavioral phenotypes of the two mutants are similar. By measuring only high-speed movements above 80 mm/s, we can effectively assess hyperactivity and loss-of-posture, and *scn1lab* KO zebrafish showed a marked increase in movement compared with WT at these speeds. Based on this *scn1lab* KO zebrafish model, we conducted phenotype-based screening and identified a novel hit compound with an oxadiazolone scaffold. In a subsequent SAR study, we identified compound **20e**, which showed outstanding antiseizure efficacy without significant differences in WT larvae. In an in vivo BBB penetration test, **20e** demonstrated B/P ratios of 4.36 ± 0.89 in the mice and 1.75 ± 0.57 in the adult zebrafish, suggesting good BBB penetration and its effective antiseizure potential. Furthermore, the bioavailability of **20e** was estimated to be 84.3% after oral administration. **20e** exhibited good liver microsomal stability and no significant inhibition of hERG, did not show mutagenic potential in the AMES test, and had an LD_50_ value greater than 250 mg/kg in the acute toxicity study. Electroencephalogram analysis is a widely considered tool to diagnose epileptic seizures.^17^ Recently, noninvasive multichannel electroencephalographic recording in larval zebrafish became available for evaluating seizure-like events.^18^ Treatment of **20e** decreased both the number and duration of ictal-like events in the KO zebrafish larvae. Furthermore, **20e** demonstrated antiseizure effects at lower concentrations than FFA and CBD, while showing no significant differences in WT larvae.

Oral administration of compound **20e** significantly reduced the seizure severity and prevented GTCS in *SCN1A* ^+/–^ mice. It also delayed seizure onset and increased seizure threshold temperature, indicating enhanced resistance to heat-induced seizures. These effects suggest that **20e** modulates neuronal excitability relevant to SMEI pathology. Additionally, **20e** reduced hyperactivity in *SCN1A* ^+/–^ mice without affecting the WT behavior, indicating mutation-specific behavioral rescue. Neurotransmitter profiling was conducted and the endogenous concentrations of neurotransmitters were determined using mass spectrometry. The application of mass spectrometry-based neurotransmitter profiling has proven beneficial in biomarker discovery and the optimization of drug candidates for CNS research.^19^ **20e** showed notable neurological changes by elevating the levels of 5-HT and GABA in both WT and *scn1lab* KO zebrafish larvae. This study underscores the importance of neurochemical research in advancing our understanding of CNS disorders and refining therapeutic strategies. Additionally, the qRT-PCR results showed that TPH2, which plays a key role in 5-HT synthesis in the brain, exhibited a dose-dependent increase in expression upon **20e** treatment, suggesting that **20e** increases 5-HT levels through TPH2. By enhancing endogenous 5-HT production rather than directly stimulating receptors, **20e** may provide more physiological regulation of serotonergic tone while reducing the risks of receptor desensitization or overstimulation associated with chronic agonist exposure. Critically, this *TPH2* upregulation occurred specifically in *SCN1A*-deficient models—no significant *TPH2* upregulation was observed in WT zebrafish, WT mice, or control organoids, whereas significant dose-dependent *TPH2* upregulation was found only in scn1lab KO zebrafish, *SCN1A* ^+/–^ mice, and mutant cerebral organoids. Furthermore, we confirmed that the antiseizure effect of **20e** was significantly attenuated when coadministered with telotristat, a TPH1/2 inhibitor, in the zebrafish Dravet syndrome model (Figure S6). In addition, **20e** treatment was associated with enhanced GABAergic function, as evidenced by increased GABA levels and normalization of electrophysiological network activity, including reduced mean firing rate and burst frequency in GABAergic neuronal networks. These GABAergic changes are consistent with the stabilization of abnormally disrupted network activity in SCN1A-deficient systems. Taken together, this mutant-specific serotonergic restoration accompanied by GABAergic network stabilization provides a mechanistic explanation for the selective suppression of seizure-related behaviors observed in our studies, wherein **20e** suppresses seizures in disease models without affecting normal behavior in WT animals. Additionally, *scn1lab* KO zebrafish larvae displayed elevated levels of stress markers, cortisol and cortisone,^20^ compared to WT zebrafish. These levels were restored to normal by compound **20e** (Figure S4). Expression of inflammatory markers, *interleukin 1β* (*il1β*) and *tumor necrosis factor α* (*tnfα*), was increased in KO zebrafish larvae at 9 dpf. Interestingly, while the level of expression of *il1β* was significantly reduced following **20e** treatment, the level of expression of *tnfα* was upregulated in KO zebrafish larvae (Figure S5). This suggests that the **20e** does not act as a broad-spectrum anti-inflammation but rather exerts selective immunomodulatory effects. The combined data suggest that compound **20e** could exhibit antiseizure efficacy by modulating these endogenous substances. This modulation could potentially reduce the seizure behavior observed in SMEI models. Therefore, our findings propose that the endogenous alterations induced by compound **20e**, including 5-HT, GABA, cortisol, and cortisone, could serve as therapeutic targets in biological systems.

We demonstrate that compound **20e** exerts a potent, dose-dependent inhibitory effect on neuronal hyperactivity in patient-derived iPSC-based neuronal networks, with significant reductions in spike and burst activity particularly evident at concentrations above 1 μM. These effects were more pronounced in SCN1A^+/–^ models, indicating that **20e** preferentially targets hyperexcitable circuits relevant to SMEI. In addition to reducing overall neuronal firing, **20e** also disrupted network-level synchronization, a hallmark of epileptiform activity. Although variability in burst duration and frequency was observed, likely reflecting the complexity of neural network regulation, the consistent suppression of hyperactivity highlights the therapeutic potential of compound **20e**.

## Conclusion

We established Nav1.1 (*scn1lab*) KO zebrafish to conduct chemical screening and identified novel 1,3,4-oxadiazol-2­(3*H*)-one derivatives for SMEI treatment. In the KO zebrafish model, compound **20e** showed significant antiseizure efficacies in behavioral analysis, which are better than those of marketed drugs. Compound **20e** exhibited favorable in vivo pharmacokinetic (PK) properties for oral administration with reasonable BBB penetration in mice. Safety evaluation indicated no significant hERG inhibition, cytotoxicity, mutagenic potential, and acute toxicity. Compound **20e** efficiently reduced the total distance moved and the mean velocity in the *SCN1A* ^+/–^ mice model of SMEI. It improved pathological spike and burst activity in severe myoclonic epilepsy of infancy (SMEI) patient-derived neurons. For the mode of action, compound **20e** increased the concentration of 5-HT through KO-specific upregulation of the *TPH2* gene, representing a mechanistically distinct approach from fenfluramine’s direct receptor activation. The multimechanistic profile encompassing serotonergic, GABAergic, anti-inflammatory, and network modulatory effects, combined with superior potency and KO-specific selectivity, distinguishes **20e** as a novel therapeutic candidate. For mode of action, compound **20e** increased the concentration of 5-HT through the increase of the *TPH2* gene. Taken together, these results underscore the therapeutic potential of compound **20e** as a promising candidate for the treatment of SMEI.

## Experimental Section

### General

All solvents and chemicals were used as purchased without further purification. All reported yields are isolated after column chromatography or crystallization. ^1^H and ^13^C NMR spectra were recorded on JEOL JNM-ECS400 spectrometers at 400 MHz for ^1^H NMR and 100 MHz for ^13^C NMR, respectively. Chemical shifts (δ) are expressed in parts per million relative to tetramethylsilane as an internal standard, and CDCl_3_, DMSO-*d*_6_, and CD_3_OD were used as solvents. The multiplicity of the peak is expressed by s (singlet), d (doublet), t (triplet), q (quartet), dd (doublet of doublets), td (triplet of doublets), qd (quartet of doublets), dt (doublet of triplets), and m (multiplet). HRMS data were obtained by Impact II (Bruker, USA). HPLC analyses were performed with a Waters Agilent HPLC system equipped with a PDA detector and an Agilent SB-C18 column (1.8 μm and 2.1 × 50 mm). The mobile phase consisting of buffer A (ultrapure H_2_O containing 0.1% trifluoroacetic acid) and buffer B (chromatographic-grade CH_3_CN) was applied at a flow rate of 0.3 mL min^–1^. All compounds are >95% pure by HPLC analysis.

### Synthesis of Compound **20e**

#### 3-(2-Chloro-4-fluorobenzyl)-5-(2-(trifluoromethyl)­pyridin-4-yl)-1,3,4-oxadiazol-2­(3*H*)-one (**20e**)

Step 1. Ethyl 2-trifluoromethylpyridine-4-carboxylate **13e** as an aryl ester (500 mg, 2.283 mmol) was dissolved in ethanol. Hydrazine hydrate (457.18 mg, 9.132 mmol) was added in the reaction mixture. The mixture was heated for reflux and stirred for 6 h. The solvent was removed under reduced pressure. The residue was diluted by water and extracted with ethyl acetate. The organic phase was dried over anhydrous sodium sulfate and concentrated in vacuo to acquire 2-(Trifluoromethyl)-4-pyridinecarboxylic acid hydrazide **14e** (413 mg, 2.015 mmol, 88%). ^1^H NMR (400 MHz, DMSO-*d*_6_) δ 10.35 (s, 1H), 8.92 (d, J = 4.9 Hz, 1H), 8.21 (s, 1H), 8.06 (dd, J = 4.9, 0.9 Hz, 1H), 4.64–4.82 (2H).

Step 2. 2-(Trifluoromethyl)-4-pyridinecarboxylic acid hydrazide **14e** (413 mg, 2.015 mmol) and 2 equiv of diethylpropylamine (0.702 mL, 4.029 mmol) were dissolved in THF (10 mL) at 0 °C. Triphosgene (239.74 mg, 0.806 mmol) dissolved in THF was added slowly dropwise. After the mixture was stirred for 1 h at 0 °C, the reaction mixture was allowed to reach room temperature and heated to reflux for 6 h. After cooling to room temperature, the solvent was removed under reduced pressure. The residue was purified by silica gel column chromatography to give 5-(2-(trifluoromethyl)­pyridin-4-yl)-1,3,4-oxadiazol-2­(3*H*)-one **19e** (315 mg, 1.364 mmol, 68%). ^1^H NMR (400 MHz, DMSO-*d*_6_) δ: 13.11 (s, 1H), 8.96 (d, *J* = 5.2 Hz, 1H), 8.09 (s, 1H), 8.06 (d, *J* = 4.9 Hz, 1H).

Step 3. 5-(Pyridin-4-yl)-1,3,4-oxadiazol-2­(3*H*)-one **19e** (378.4 mg, 2.318 mmol) and 2-chloro-4-fluorobenzyl bromide (570.17 mg, 2.551 mmol) were dissolved in DMF (2 mL) under N_2_ condition. Sodium hydride 60% dispersion in mineral oil (101.87 mg, 2.551 mmol) in DMF was added slowly, dropwise, to the solution. The solution was heated to 60 °C and stirred for 4 h. The solvent was removed under reduced pressure. The residue was diluted with water (20 mL) and extracted with ethyl acetate (3 × 50 mL). The combined organic layer was washed with brine (20 mL). The crude product was purified by silica gel column chromatography to give 3-(2-chloro-4-fluorobenzyl)-5-(pyridin-4-yl)-1,3,4-oxadiazol-2­(3*H*)-one **20e** (463.7 mg, 1.517 mmol, 65%). ^1^H NMR (400 MHz, DMSO-*d*_6_) δ 8.96 (d, J = 4.6 Hz, 1H), 8.05 (d, J = 5.5 Hz, 2H), 7.62 (dd, J = 8.4, 6.3 Hz, 1H), 7.55 (dd, J = 8.9, 2.4 Hz, 1H), 7.28 (td, J = 8.4, 2.2 Hz, 1H), 5.09 (s, 2H); ^13^C NMR (101 MHz, DMSO-*d*_6_) δ 164.0, 161.5, 153.2, 152.7, 151.2, 149.0, 148.6, 148.3, 147.9, 134.5, 134.4, 133.8, 133.4, 133.3, 129.6, 129.6, 126.2, 123.6, 123.5, 120.7, 118.1, 117.8, 116.8, 115.8, 115.6, 47.6; HRMS (ESI) *m*/*z* calculated for C_15_H_9_ClF_4_N_3_O_2_ [M + H]^+^ 374.03139, found 374.03036; HPLC purity 98.1616%.

### Biological Experiments

#### Experiments of SMEI Model in Zebrafish Larvae

Larvae clutches were bred from *scn1lab* ^*kri111/+*^. Homozygous *scn1lab* KO larvae showing hyperpigmentation and age-matched sibling larvae were used. 6-dpf larvae were placed individually into a 96-well plate. The microplate was placed inside a behavioral tracking device and acclimated to the dark condition for 30 min. DMSO or compounds were added into each well. The behavioral data of each larva were recorded using EthoVision 15 software (Noldus) connected to the DanioVision chamber (Noldus) for 30 min. Seizure-like movements were defined as previously described. Immunohistochemistry for 5-HT was conducted as previously described.^21^ Larvae were placed into a 6-well plate with a cell strainer (SPL, 93100) to ensure rapid fixation. After treatment for 4 h, each cell strainer was immediately transferred into 4% paraformaldehyde/4% sucrose in PBS at 4 °C. After overnight fixation, larvae were washed with 0.25% Triton X-100/PBS (PBTx). Samples were used to dissect the brain from larvae to highly penetrate antibody. Dissected brains were incubated in 1 mg/mL collagenase (Sigma-Aldrich, C9891) for 1 h and blocked overnight in 2% normal goat serum/2% DMSO in PBTx at 4 °C. Then, dissected brains were incubated overnight in blocking solution with antibodies at 4 °C. Rabbit 5-HT antibody (Sigma-Aldrich, S5545) and goat anti-Rabbit IgG (Invitrogen, A-11034) were used as the primary and secondary antibodies, respectively. After some washing with PBTx, dissected brains were mounted in 1% low-melting agarose and imaged using a K1-Fluo confocal fluorescence laser scanning microscope (Nanoscope Systems).

### Neurotransmitter and Neurosteroid Analysis

To investigate brain-specific metabolites, including neurotransmitters and neurosteroids, in zebrafish larvae, we performed targeted analysis using LC-MS/MS, according to our previously reported methods.^22^ Briefly, 30 pooled zebrafish larvae at the 6 dpf stage were exposed to 5 μM **20e** for 4 h in a 6-well plate, then washed and collected into a 1.7 mL tube. For neurotransmitter analysis, wet larvae were snap-frozen using liquid nitrogen, and 300 μL of distilled water (DW) was added, followed by homogenization using a sonicator in a 1.7 mL tube on ice. The homogenates were mixed with methanol containing 1% formic acid. Endogenous neurotransmitters were extracted by vortexing for 5 min, and the clear supernatant was transferred into LC vials after centrifugation at 15,000 rpm for 10 min. For neurosteroid analysis, 1 mL of methanol/acetic acid (99:1 v/v) was used to homogenize the samples. The homogenate was then centrifuged for 5 min at 12,000 rpm, and the remaining pellet was extracted twice with methanol/acetic acid (99:1 v/v). The pellet was subsequently resuspended in 1 mL of methanol/water (10:90 v/v), and the mixture was loaded onto a solid-phase extraction cartridge (Oasis PRIME HLB, Waters). After eluting twice with 1 mL of methanol, the sample was evaporated under nitrogen gas. Final eluates were transferred to an LC vial. Neurotransmitters and neurosteroids were quantitatively analyzed using LC-MS/MS.

### Experiments of SMEI Model in Mouse

*Scn1a* ^*tm1Kea*^ mice on a pure 129S6/SvEvTac (129) inbred strain background were generated by homologous recombination in TL1 ES cells (129S6/SvEvTac). The mouse line Scn1a^tm1Kea^ has been maintained as a coisogenic strain by continuous backcrossing of null heterozygotes to 129 (129.Scn1a+/–. 129S-Scn1a^tm1Kea/Mmjax^ (#024761) heterozygous mice and WT animals were obtained from Jackson Laboratory, USA, and male 129S-Scn1atm1Kea/Mmjax heterozygous mice (Jackson Laboratory, USA) were crossed with female WT C57BL/6 animals. Thus, Scn1a+/– and WT littermate mice were obtained. These lines were maintained, bred together, and used in this study (*n* = 5 to 7 per group). All experiments were approved by the Institutional Animal Care and Use Committee at the Korea Research Institute of Chemical Technology and were conducted in accordance with the guidelines of the Ministry of Food and Drug Safety for the care and use of laboratory animals, as well as the policy on humane care and use of laboratory animals (approval number: 2024-7A-09-01).

The mice were housed in a specific pathogen-free (SPF) barrier facility with a 12 h light/12 h dark cycle and had ad libitum access to food and water. The genotype of the mice was confirmed by PCR, ensuring the successful generation and maintenance of the Scn1a mutant mouse model. CBD was purchased from Sigma. CBD was dissolved in a 1:1:18 ratio in 100% ethanol, cremophor, and 0.9% saline, respectively.^23^ **20e** was prepared in DMSO:PEG400:water = 10:60:30 (v/v) for acute administration. This is because it is a good general-purpose solvent and maintains excipient consistency from test to test. All drugs were administered at a dose of 10 mL/kg. All drug compounds were dosed and tested based on their previously determined time-to-peak effect in the maximal seizure.^24^ Seizures were induced as previously described^25^ with some modifications. Briefly, mice were placed in a Plexiglas cylinder with an infrared heating lamp (250 W, HL-1, Physitemp Instruments Inc.) held in a fixed position. A rectal temperature probe (RET-4, Physitemp Instruments Inc.) was carefully inserted and taped to the mouse’s tail. The animal’s core body temperature was monitored by connecting a temperature probe to a temperature controller (TCAT-2DF, Physitemp Instruments Inc.). In this study, male and female mice aged 13 to 15 weeks were administered an intraperitoneal injection of 100 mg/kg of CBD 1 h prior to heat induction. Additionally, 30 min before the heat exposure, they received a gavage dose of 5 mg/kg of **20e**. The temperature of the rectal probe inserted into the mouse was adjusted at 1 °C intervals to 37.5 °C, 38.5 °C, 39.5 °C, 40.5 °C, 41.5 °C, 42.5 °C, and 43.5 °C. The temperature was observed and recorded for approximately 2 min. The effects of each drug were confirmed through a *t*-test by comparing the seizure time and temperature. The overall Modified Racine’s Scale (seizure severity)^26^ reached levels 3 (first seizure) and 5 (GTCS; tonic-clonic seizures). Scale scores were evaluated as significant. In particular, because the Racine Scale cannot reliably assess seizures with severity <3 in mice, we limited the assessment of seizure severity to those with a Racine score of 3–5. Each mouse was placed near the lower left wall of a 40 × 40 cm open arena. Mice were placed in an apparatus and allowed to freely explore for 15 min. Mouse movements were analyzed with a GigE camera with a lens and PC-based video tracking software (EthoVision XT 17, Noldus Technology). Based on the data profile, we analyzed the results for total travel distance and speed.

### Neuron Culture

Neuron culture began on Day 1, defined as the day doxycycline was added. The cells were cultured in NGM Media, replaced daily, for about 1 week. The day before reaching 1 week, astrocytes (1 × 10^5^ cells) were seeded onto Matrigel-coated coverslips in 24-well plates. Neurons were dissociated into single cells by using Accutase. A portion (5 × 10^4^ cells) was used for electrophysiology measurements, while another (5 × 10^4^ cells) was cultured in NGM Media supplemented with 10% Mouse Astrocyte Conditioned Medium (ScienCell Research Laboratories, Carlsbad, California, United States) for ICC and cDNA synthesis. For microelectrode array (MEA) recordings, astrocytes were seeded onto Matrigel-coated coverslips (5 × 10^4^ cells), followed by the addition of 1.5 × 10^4^ GABAergic and 3.5 × 10^4^ glutamatergic neurons, making a total of 1 × 10^5^ cells. Media changes were performed every 2 days, with 50% of the media refreshed during each change.

### MEA Measurement and Data Analysis

MEA recordings were conducted using a 24-well Maestro Edge system (Axion BioSystems, Atlanta, Georgia, United States). Each MEA well contained a total of 1 × 10^5^ cells, comprising 1.5 × 10^4^ GABAergic neurons, 3.5 × 10^4^ glutamatergic neurons, and 5 × 10^4^ astrocytes. Neural network activity from iPSC-derived neurons and their calibrated counterparts was recorded for 15 min at 37 °C in a chamber maintained with 95% O_2_ and 5% CO_2_. The recordings were sampled at 10 kHz, filtered with a 100 Hz high-pass and a 3500 Hz low-pass filter. Spikes were detected at ±4.5 standard deviations. Metrics such as mean firing rate were calculated as the average spike frequency across all channels in a well. Burst activity was defined as channels exhibiting at least five spikes per burst, with a minimum interburst interval of 100 ms and a threshold of 0.4 bursts/s. Network bursts were characterized as synchronized bursts across more than 35% of channels in a well. Data analysis was performed using Axion software (AxIS) following the manufacturer’s guidelines. Patient-derived neurons were analyzed from 3 weeks to 6 weeks. Drug treatment was administered at 6 weeks, followed by measurements. Each drug was applied in a stepwise manner, starting from a low concentration and gradually increasing to higher concentrations. After drug application, a reaction time of 10 min was allowed, and spontaneous neuronal activity was recorded for 15 min.

### Experiments of Cerebral Organoid

Induced pluripotent stem cells (iPSCs, IMR90-4, WiCell, Madison, USA) served as the initial seeding material for the culture of cerebral organoids. These cells were seeded into U-bottom ultralow-attachment 96-well plates (Corning). The neural induction medium was composed of Dulbecco’s Modified Eagle Medium/Nutrient Mixture F-12 (DMEM/F12) supplemented with 1% GlutaMAX, 1% MEM-NEAA (Minimum Essential Medium Non-Essential Amino Acids), 15% knockout serum (Thermo Scientific), 0.1 nM β-mercaptoethanol, 100 nM LDN-193189, 10 μM SB431542, and 2 μM XAV939 (Sigma-Aldrich, St. Louis, MO, USA). The cells underwent static culture conditions for 10 days, with media changes performed every other day. On day 10, the cells were transferred to ultralow-attachment 6-well plates (Corning) to initiate neural differentiation. The neural differentiation medium (NDMI) was formulated by combining DMEM/F12 and neurobasal medium in a 1:1 ratio. To this mixture, an N2 supplement, a B27 supplement without vitamin A (both from Invitrogen), 1% MEM-NEAA, 1% GlutaMAX (Thermo Scientific), and human insulin solution (Sigma-Aldrich) were added. Subsequently, the cells were cultured on an orbital shaker at 80 rpm for 8 days. From day 18 onward, the cells were transitioned to neural differentiation medium (NDMII) supplemented with B27 with vitamin A (Thermo Scientific), brain-derived neurotrophic factor, cyclic adenosine monophosphate, and ascorbic acid (Sigma-Aldrich) to facilitate neural maturation. The medium was refreshed every 4 days to sustain the maturation process of the cerebral organoids. The culture period spanned 120 days, during which the cells underwent successive stages of neural induction, neural differentiation, and neural maturation, culminating in the development of mature cerebral organoids.

### Quantitative Real-Time PCR

Total RNA was isolated from zebrafish larvae (20 pooled) after exposure of **20e** in WT or homozygous *scn1lab* KO using TRIzol reagent (Invitrogen, catalog no. 15596026) and purified according to manufacturer’s protocol. qRT-PCR was performed using Verso SYBR Green 1-Step qRT-PCR Low ROX Mix (Thermo Scientific, cat# AB-4106/A). PCR cycling conditions were as follows: cDNA synthesis at 50 °C for 15 min, initial denaturation at 95 °C for 15 min, followed by 40 cycles of 95 °C for 15 s, 60 °C for 30 s, and 72 °C for 30 s. Total RNA was extracted from frozen mouse brain tissues and human cerebral organoids using the RNeasy Plus Mini Kit (Qiagen, Valencia, CA, USA), following the manufacturer’s instructions. RNA concentration and purity were assessed using a NanoDrop One spectrophotometer (Thermo Fisher Scientific, Waltham, MA, USA). Subsequently, 2 μg of total RNA from each sample was reverse transcribed into complementary DNA (cDNA) using the AccuPower RT PreMix (Bioneer Inc., Seoul, Korea). Quantitative gene expression analysis was performed using gene-specific primers for human *TPH2* and mouse *Tph2*. qRT-PCR was conducted using the Rotor-Gene Q system (Corbett Research, Mortlake, Australia) with the QuantiTect SYBR Green PCR Kit (Qiagen). Each 20 μL PCR reaction contained 10 μL of SYBR Green master mix, 2 μL of 10 pmol/μL forward and reverse primers, and 1 μL of cDNA template. PCR cycling conditions were as follows: initial denaturation at 95 °C for 15 min, followed by 40 cycles of 95 °C for 30 s, 60 °C for 30 s, and 72 °C for 30 s. Relative mRNA expression levels were calculated using the ΔCt method, where ΔCt = Ct (target gene) – Ct (endogenous gene). All data were normalized to *β-actin*, *18S rRNA* expression to account for variability in input RNA and transcriptional activity. Oligos and primer sets are listed in Table S1.

## Supplementary Material





## Notes

Experimental procedures, supplementary figures, Synthetic procedure, ^1^H NMR, ^13^C NMR, HRMS spectra, and HPLC assessment of purity for all of final compounds (PDF)
Molecular formula strings (CSV)

## Abbreviations Used

ASD: antiseizure drugs
CBD: cannabidiol
LGS: Lennox–Gastaut syndrome
AUC_t_: areas under the plasma concentration–time curve
BBB,: blood–brain barrier
CL: total clearance from plasma
CNS: central nervous system
CYP: cytochrome P450
DCM: dichloromethane
Dipea: N,N-diisopropylethylamine
DMF: N,N-dimethylformamide
DMSO: dimethyl sulfoxide
EA: ethyl acetate
EtOH: ethanol
FFA: fenfluramine
hERG: human ether-a-go-go-related gene
HPLC: high-performance liquid chromatography
HRMS: high-resolution mass spectrometry
HSC: hepatic stellate cell
IC_50_: half-maximal inhibitory concentration
IP: intraperitoneal
IV: intravenous;
LC-MS/MS: liquid chromatography–mass spectrometry/mass spectrometry
NMR: nuclear magnetic resonance
SAR: structure–activity relationship
*scn1lab*: sodium channel, voltage-gated, type 1 like, alpha b
SEMI: severe myoclonic epilepsy of infancy
STP: stiripentol
*T*_1/2_: terminal half-life
THF: tetrahydrofuran
V_ss_: steady-state volume ofdistribution
