# Supplementary material for: Development of Novel Small-Molecule Targeting SCN1A-Associated Severe Myoclonic Epilepsy of Infancy
Source: J Med Chem. 2026 Jan 23;69(3):3362–77. doi: 10.1021/acs.jmedchem.5c03293 (PMC12910655; doi:10.1021/acs.jmedchem.5c03293)
Supplement: Supplementary file 1 [file jm5c03293_si_001.pdf]

Supporting information

Development of Novel Small-Molecule Targeting SCN1A-associated Severe Myoclonic Epilepsy of Infancy

Dong Gun Kim[a,‡], Kyu-Seok Hwang[b,‡], Se Hwan Ahn[a,‡], Seong Soon Kim[b,‡], Yuji Son[b], Sung Bum Park[b], Won Hoon Jung[b], Dae-Seop Shin[b], Sung Hee Cho[b], Byeong Wook Choi[a], Pyeongkeun Kim[a], Yerim Heo[a], Minhee Kim[b], Jung Yoon Yang[b], Kyeong-Ryoon Lee[c,d], Hyang-Ae Lee[e], Jihun Kim[f], Hoon-Chul Kang[f], Ki Young Kim[b,*], Myung Ae Bae[b,c*], Jin Hee Ahn[a,g*]

[a] *Department of Chemistry, Gwangju Institute of Science and Technology, Gwangju 61005, Republic of Korea*
[b] *Therapeutics & Biotechnology Division, Korea Research Institute of Chemical Technology, Daejeon 34114, Korea., Republic of Korea*
[c] *Department of Medical Chemistry and Pharmacology, University of Science & Technology, Daejeon 34113, Republic of Korea*
[d] *Laboratory Animal Resource Center, Korea Research Institute of Bioscience and Biotechnology, Ochang 28116, Republic of Korea*
[e] *Center for Biomimetic Research, Korea Institute of Toxicology, Daejeon 34114, Republic of Korea*
[f] *Division of Pediatric Neurology, Department of Pediatrics, Severance Children's Hospital, Yonsei University College of Medicine, Epilepsy Research Institute, Seoul, 03722, Republic of Korea*
[g] *JD Bioscience Inc, 208 Cheomdan-dwagiro, Buk-gu, Gwangju 61011, Republic of Korea*

‡ These authors equally contributed to this work.

*To whom correspondence should be addressed:

Jin Hee Ahn, PhD
Department of Chemistry, Gwangju Institute of Science and Technology
Tel: +82-62-715-4621, Fax: +82-62-715-2866
E-mail: jhahn@gist.ac.kr

Myung Ae Bae, PhD
Therapeutics & Biotechnology Division, Korea Research Institute of Chemical Technology, Daejeon 34114, Korea
Tel: +82-42-860-7084, Fax: +82-42-860-7459
E-mail: mbae@krict.re.kr

Ki Young Kim, PhD
Therapeutics & Biotechnology Division, Korea Research Institute of Chemical Technology, Daejeon 34114, Korea
Tel: +82-42-860-7084, Fax: +82-42-860-7459
E-mail: kykim@krict.re.kr

List of contents

Expriemental section S3

Figures S8

Chemistry S13

HPLC purity analysis S22

^{1}H NMR and ^{13}C NMR spectra S30

Experimental section

General

All solvents and chemicals were used as purchased without further purification. All the reported yields are isolated yields after column chromatography or crystallization. ^{1}H NMR spectra and ^{13}C spectra were recorded on a JEOL JNM-ECS400 spectrometers at 400 MHz for ^{1}H NMR and 100 MHz for ^{13}C NMR respectively. The chemical shift (δ) is expressed in ppm relative to tetramethylsilane (TMS) as an internal standard, and chloroform-*d*, DMSO-d_6, methanol-d_4 used as solvents. Multiplicity of peaks is expressed as s (singlet), d (doublet), t (triplet), q (quartet), dd (doublet of doublets), td (triplet of doublets), qd (quartet of doublets), dt (doublet of triplets), and m (multiplet). High resolution mass spectra (HRMS) were obtained by fast atom bombardment (FAB) ionization technique (magnetic sector – electric sector double focusing mass analyzer) from the KBSI (Korea Basic Science Institute Daegu Center) and electrospray ionization(ESI) technique (Quadrupole-orbitrap mass analyzer) from the GAIA(GIST Advanced Institute of Instrumental Analysis). Purity of all tested compounds was ≥ 95 %, as estimated by Highperformance liquid chromatography (HPLC) analysis. Samples were analyzed on a Waters Agilent HPLC system equipped with a PDA detector and a Waters SB-C18 column (1.8 μM, 2.1 × 50 mm). The mobile phase was used with buffer A (ultrapure H_2O containing 0.1 % TFA) and buffer B (chromatographic grade CH_3CN). Flow rate was 0.5 mL/ min. All compounds are >95% pure by HPLC analysis.

Liquid chromatography-mass spectrometry (LC-MS/MS) analysis. Quantitative analysis of drug performed using an ultra-performance liquid chromatography (ACQUITY UPLCTM system, Waters Corporation, Milford, MA, USA) coupled with a Xevo TQ-S triple quadrupole mass spectrometer (Waters Corporation). The chromatographic separation of drug was conducted using a BEH C18 column (50 mm × 2.1 mm, i.d. 1.7 μm; Waters), with a BEH C18 VanGuard pre-column (2.1 mm × 5 mm, i.d. 1.7 μm; Waters) at 40°C, with an injection volume of 3 μl. Multiple reaction monitoring (MRM) mode with electrospray positive ionization (ESI+) was used for the quantification of drug. The mass parameters were optimized as follows: desolvation temperature, 550°C; desolvation gas flow, 1000 L/h; cone gas flow, 150 L/h; nebulizing gas flow, 7 bars.

Stability in liver microsome and plasma. For the liver microsomal stability, each compound was incubated with the mouse or human liver microsome (Corning, USA), at a final concentration of 1 μM,

in the presence of NADPH regeneration system in 0.1 M potassium phosphate buffer (pH 7.4) for 0.5 h at 37℃ in shaking incubator. For the plasma stability, each compound was incubated with mouse or human plasma (Innovative research, MI, USA) at a final concentration of 5 µM for 4 h at 37℃ in shaking incubator. The reaction was terminated by adding four volumes of ice-cold acetonitrile and then filtered through 0.2 mm GHP filter plates (Acro Prep™, Pall Corporation, Ann Arbor, USA), and analyzed by LC-MS/MS. The percentage of the parent compound remaining was calculated by comparing peak areas.

Plasma protein binding assay. To evaluate the binding of 20e to plasma proteins, we used mouse and human plasma with a rapid equilibrium dialysis device (Thermo Fisher Scientific, Waltham, MA, USA). In brief, 200 µL of plasma containing 5 µM of 20e was placed into the RED plasma chamber (indicated by the red ring on the dialysis insert), while 350 µL of BupH™ phosphate-buffered saline (PBS, pH 7.4, Thermo Fisher Scientific) was added to the white chamber. The plate was sealed with self-adhesive tape and incubated at 37°C for 4 h in a shaking incubator. After equilibrium was reached, samples from both plasma and PBS were collected, mixed with four volumes of acetonitrile, vortexed for 10 min, and centrifuged at 15,000 RPM and 4°C for 10 min. The supernatants were then analyzed for the parent drug using LC-MS/MS. The percentages of unbound (Fub) and bound (Fu) fractions of the drug were calculated using the relevant equations:

F_ub (%)   C_buffer/C_plasma ×100

F_u (%)=100-F_ub%

C buffer = drug concentration in the buffer chamber

C plasma = drug concentration in the plasma chamber

CYP cocktail inhibition assay using LC-MS/MS. The cocktail CYP inhibition assay was performed according to previously reported method[1]. Briefly, various concentrations of GM-91466 and ketoconazole (as a positive control for inhibiting CYP 3A4) were dissolved in 0.1 M potassium phosphate buffer (pH 7.4) to achieve final concentrations of 0.01, 0.1, 1, 10, and 100 µM. This solution was mixed with a cocktail substrate mixture consisting of: 1A2, phenacetin; 2C9, tolbutamide; 2C19, S-mephenytoin; 2D6, dextromethorphan; and 3A4, sorafenib. Human liver microsomes were added to each condition to achieve final concentration of 0.2 mg/mL, and the samples were pre-incubated for 5 min at 37°C in a shaking incubator. After pre-incubation, the samples were mixed with an NADPH regeneration system in 0.1 M potassium phosphate buffer (pH 7.4) and incubated for an additional 20 min at 37°C in a shaking incubator. The inhibitory reaction was terminated by adding two volumes of cold acetonitrile containing an internal standard, followed by filtration through 0.2 µm GHP filter plates (Acro Prep™, Pall Corporation, Ann Arbor, USA). The filtrates were analyzed by LC-MS/MS. The quantitative concentration of each metabolite (1A2, acetaminophen; 2C9, 4-OH-tolbutamide; 2C19, 4-

OH-mephenytoin; 2D6, dextrorphan; 3A4, sorafenib N-oxide) was determined using LC-MS/MS, and the half-maximal inhibitory concentration (IC50) was calculated using GraphPad software.

Cytotoxicity assay. WST is commonly method for cytotoxicity in living cells of mitochondria, which is reduced by succinate-tetrazolium(dehydrogenase) to produce orange formazan. Cytotoxicity was determined using Cyto X assay kit (LPS solution, Daejeon, Korea) according to the manufacturer's instruction. VERO, HFL-1, NIH, L929 and CHO-K1 (ATCC) cells were seeded in 96-well plates at the density of 2 x 105 cells/mL. Test compounds was prepared in 0.001, 0.01, 0.1, 1, 10 mM stock solution with DMSO. After incubating for 24h, 1 μM stock solution was treated. After 24h, Cyto X solution was added to each well and was further incubated for 2h at 37℃. The plates were measured the absorbance at 450 nm in the microplate reader device.

Binding assay for hERG channel protein. The binding assay for hERG was measured by the Predictor™ hERG Fluorescence Polarization (FP) Assay kit (Thermo Fisher Scientific, Inc., Rockford, IL, USA). The procedure was carried out in accordance with the manufacturer's protocol. Test compound was diluted with assay buffer so that the concentration was 4 times higher than the final concentration. The binding assay was conducted in 384 well black flat-bottom microplates (Corning Life Sciences, Lowell, MA, USA). The diluted test compound was taken into each well by 5 μL. The tracer (PredictorTM hERG tracer red) and membrane fraction containing the hERG channel protein (PredictorTM hERG membrane) was added to each well. The final mixture of 20 μL was consisted with 5 μL of test compound, 5 μL of tracer, 10 μL membrane fraction. After incubation for 2 h at room temperature, 2 h, the FP was measured with excitation filter of 530 nm and emission filter of 585 nm using a microplate reader (Infinite M1000PRO; Tecan, Mannedorf, Switzerland).

Parallel Artificial Membrane Permeability Assay (PAMPA). The stock solution of test compound was prepared at the concentration of 10 mM in DMSO and diluted with a phosphate buffered saline (PBS) buffer (pH 7.4) to obtain the reference solutions at 50 μM. PAMPA was conducted with a STIRWELLTM PAMPA sandwich (pION, Inc., Billerica, MA, USA) consisted with 96-well filter plate (0.45 μm PVDF membrane) acceptor plate and a 96-well polystyrene donor plate. To form lipid membranes, 5 μL GIT-0 lipid (pION, Inc., Billerica, MA, USA) was dropped and semi-dried on the bottom side of acceptor plate. The donor plate were prepared by the addition of 200 μL of working solution. The acceptor plate coated with lipid membrane was then overlayed on the donor plate without air bubble. Immediately, 200 μL of acceptor sink buffer (pION, Inc., Billerica, MA, USA) was dispensed at inner side of acceptor plate. The STIRWELLTM PAMPA sandwich was incubated for 4 h at room temperature in Gut-BoxTM (pION, Inc., Billerica, MA, USA). To acquire the peak area of

range from 230 to 500 nm, absorbances of PBS, acceptor, donor, and reference solutions were measured with Epoch ELISA reader (BioTek Instruments Inc., Winooski, VT, USA). The PAMPA Explorer Command Software (pION, Inc., Billerica, MA, USA) was used for analysis of permeability of compounds.

Pharmacokinetic study in mouse and zebrafish models. To estimate the pharmacokinetic parameter in animals, the drug was administered via intravenous (I.V., 2 mg/kg) and oral (P.O., 10 mg/kg), and intraperitoneal (I.P., 30 mg/kg) routes in seven-week-old male ICR mouse (Orient Bio Inc., Seongnam, Korea). Blood samples were collected at 0.083 (for I.V.), 0.5, 1, 2, 4, 8, and 24 h after drug administrations, and then immediately centrifuged at 10,000g for 3 min. The plasma concentrations of drug were determined by LC-MS/MS. The plasma concentration-time profiles and pharmacokinetic parameters were estimated by non-compartmental method using the nonlinear least squares regression program WinNonlin 5.3 (Pharsight, Mountain View, CA, USA). To evaluate the brain to plasma (B/P) ratio in mouse and adult zebrafish models, the drug was orally administered via P.O. at a dose of 10 mg/kg for both models. Plasma and brain tissues were collected from each animal after 0.5 h of drug administration. Brain samples were added four volume of saline for the mouse model and nineteen volume of saline for adult zebrafish, and homogenized using a probe sonicator (Ultrasonic processor VCX-130, Sonics & Materials Inc, Newtown, CT, USA). The samples were added to nine volume of acetonitrile for protein precipitation, followed by vortex and centrifugation for 10 min at 15,000 RPM.

Formation of BBB chip and sampling. The chip (BEOnchip, #BE-DOUBLEFLOW) was composed of two parallel microchannels (each channel; 375 μm x 1.5 mm x 46 mm) separated by a porous (membrane pore size; 1 μm). The membrane was coated with a mixture of laminin (1 mg/mL), collagen IV (1 mg/mL) and fibronectin (1 mg/mL) in DPBS. Coated chips were incubated at 4 °C overnight, then at 37 °C for 1 h before seeding the cells. Human brain microvascular endothelial cells (hBMEC; #1000, ScienCell, Carlsbas, CA, USA) cells (9×10^5 cells/mL) were 40 μL seeded into the bottom channel of the chip. The chip was immediately inverted and incubated at 37 °C for 4 h. After 4 h, hBMECs were re-injected into the bottom channel to mimic the vascular structure. Human brain vascular pericytes (HBVP; #1200, ScienCell) and Human astrocyte (NHA; CC-2565, Lonza, Basel, Switzerland) were mixed at 0.04×10^5 and 0.4×10^5 cells/mL, respectively, and introduced 40 μL into the top channel; the chip was in a static culture at 37 °C for 1 day and then kept in flow in the chamber for 1 day. The permeability study was carried out 48 h after the seeding cells in the BBB-on-a-chip. For apical-basolateral (A to B) permeability analysis, the hBMEC media were diluted to the final concentration of 1, 10 μM and treated in the bottom channel (apical, blood channel), and the media were collected at 24 h from the outlet reservoirs of the bottom and top, respectively. Fifteen microliters of blood-and-brain medium was mixed with 135 μL internal standard (IS) solution (5 ng/mL

disopyramide in acetonitrile). The mixture was vortexed briefly and centrifuged at 15,000× g rpm for 5 min at 4 °C. The supernatant (100 μL) was transferred to a sample vial for LC-MS/MS analysis using a liquid chromatograph (Agilent 1260) and 4000 Qtrap quadruple mass spectrometer (LC-MS/MS, AB Sciex, Foster City, CA, USA) analysis.

Cells were fixed with 4% formaldehyde and blocked on the brain-on-a-chip in phosphate-buffered saline (PBS) containing 10% fetal bovine serum at 4 °C overnight for Immunofluorescence. The primary antibodies were GFAP (1:200, 560298, BD pharmingen, NJ, USA), NG2 (1:200, ab129051, Abcam, Cambridge, UK), and CD31 (1:200, 303110, BioLegend, San Diego, CA, USA). Chips treated with corresponding Alexa Fluor-conjugated secondary antibodies (1:500, Abcam) were incubated in the dark for 2 h at room temperature. Chips were then washed in PBS. The Cells were visualized using the Lionheart FX Automated Microscope (BioTek Instruments, Winooski, VT, USA).

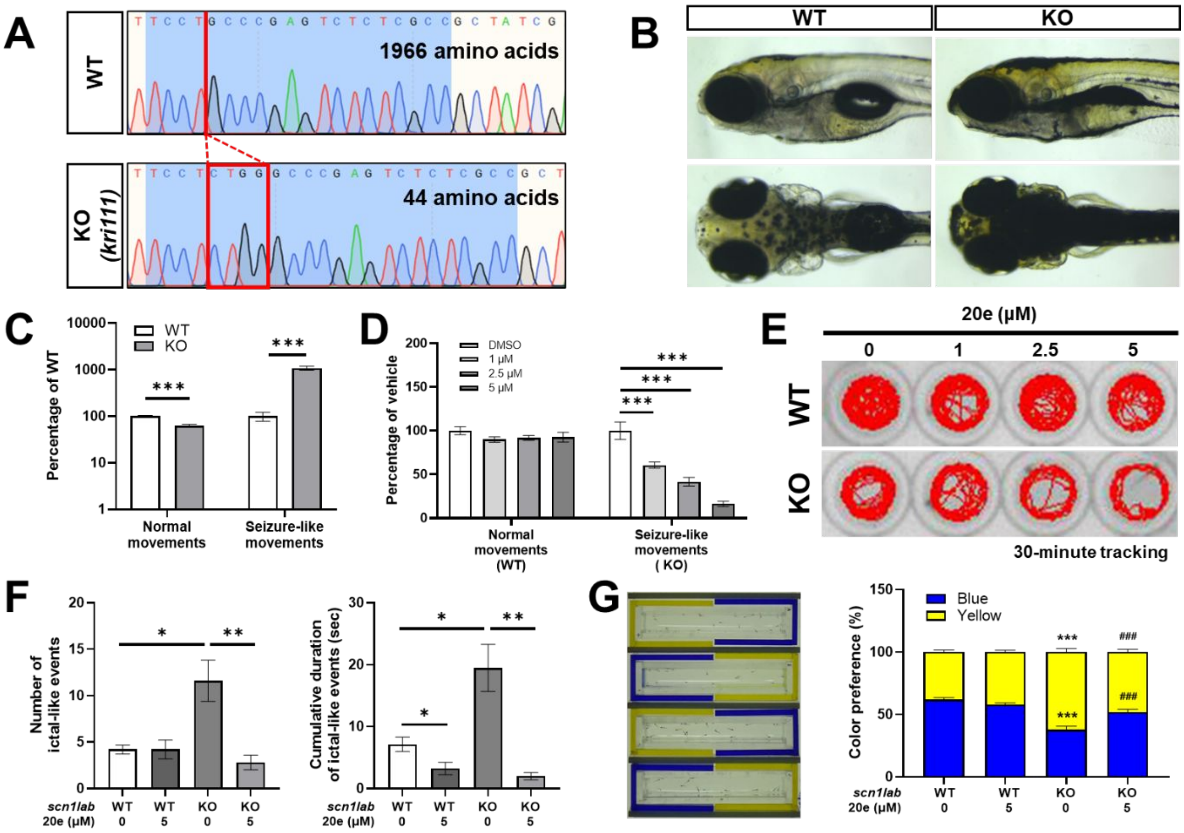

Supplementary Figure 1. Establishment of *scn1lab* KO zebrafish and evaluation of compound 20e for treatment in SEMI. a Identification of 4-bp insertion in *scn1lab* KO zebrafish. **b** Representative images of WT and *scn1lab* KO zebrafish larvae. **c** Comparison of normal movements and seizure-like movements between WT and *scn1lab* KO larvae. **d** Quantification of normal movements in WT larvae and seizure-like movements in *scn1lab* KO larvae following treatment with compound 20e. **e** Representative images of larvae tracking during 30 min. **f, g** Analysis of electroencephalogram (EEG) and color preference in WT and scn1lab KO larvae following treatment with compound 20e.

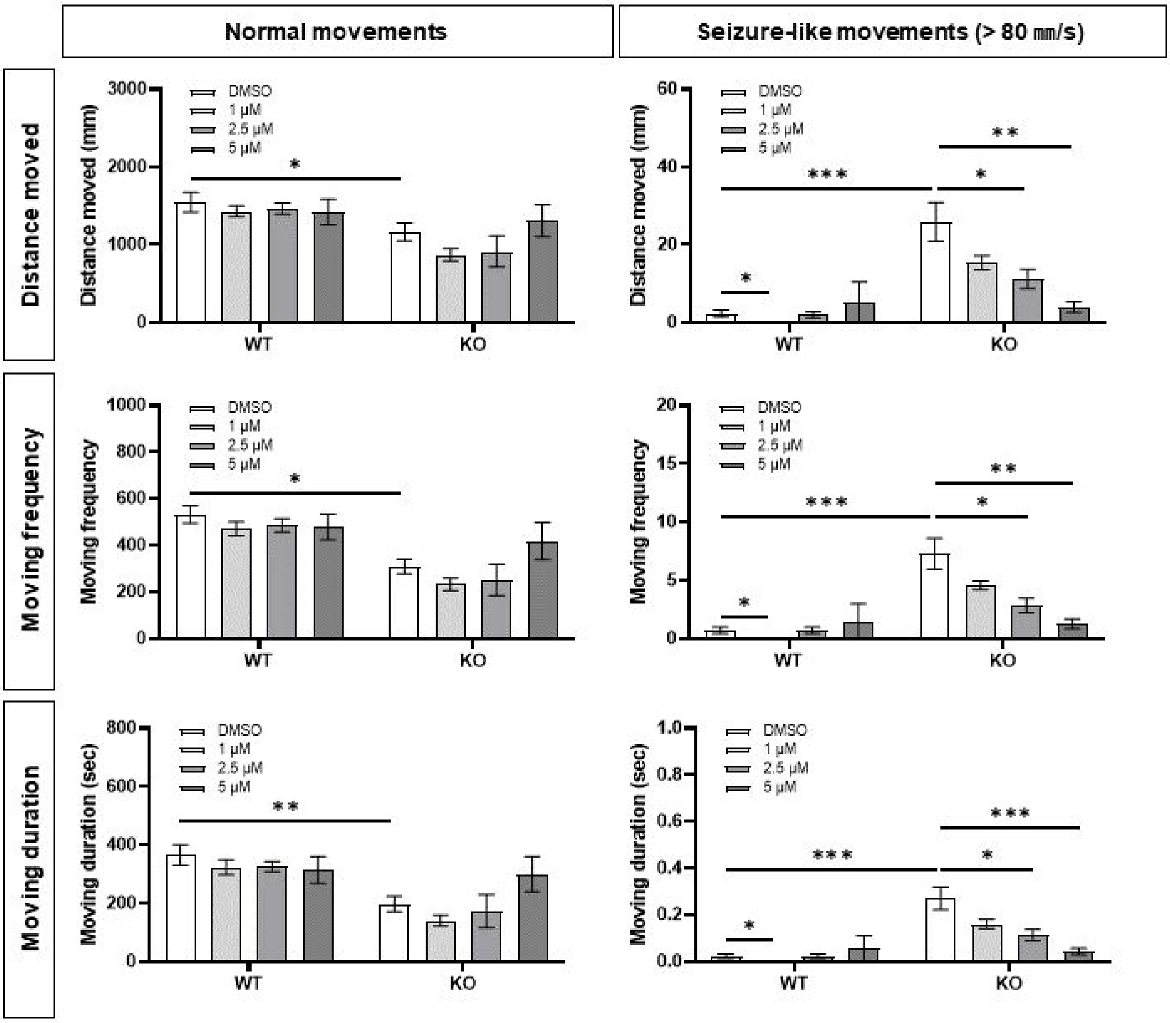

Supplementary Figure 2. Quantification of normal movements and seizure-like movements in WT and *scn1lab* KO larvae following treatment with compound 20e. Three locomotor parameters such as distance moved, moving frequency, and duration were quantified to calculate locomotor activity in zebrafish larvae. Seizure-like movements were defined as a speed above 80 mm/s, whereas normal movements were not set.

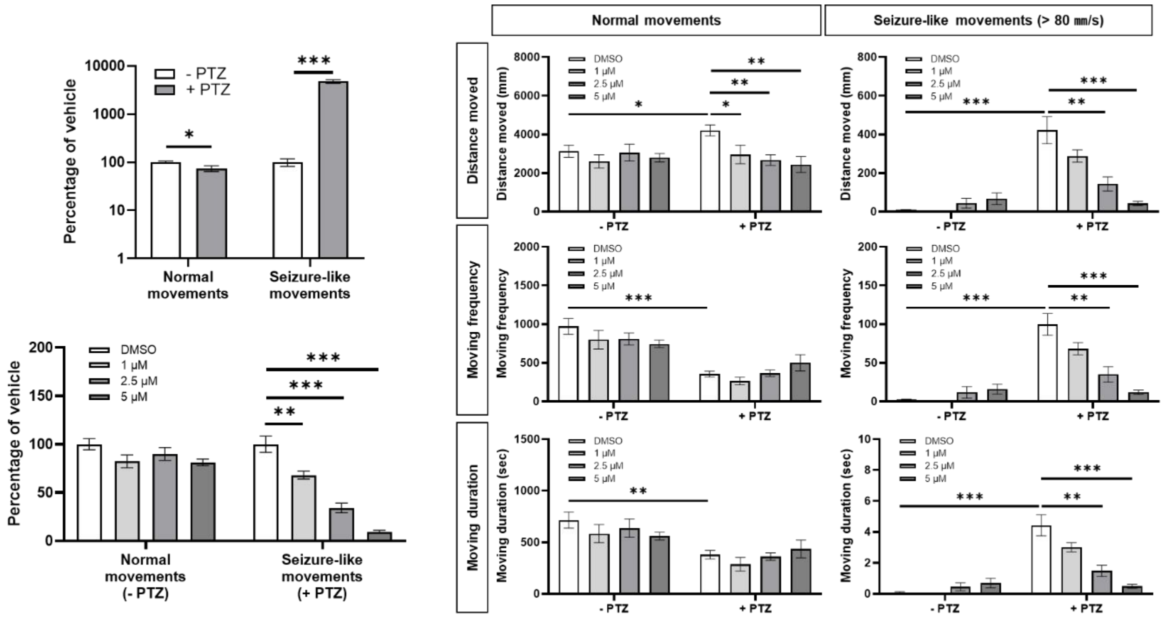

Supplementary Figure 3. Quantification of normal movements and seizure-like movements in PTZ-induced seizure larvae following treatment with compound 20e. Three locomotor parameters such as distance moved, moving frequency, and duration were quantified to calculate locomotor activity in zebrafish larvae. Seizure-like movements were defined as a speed above 80 mm/s, whereas normal movements were not set.

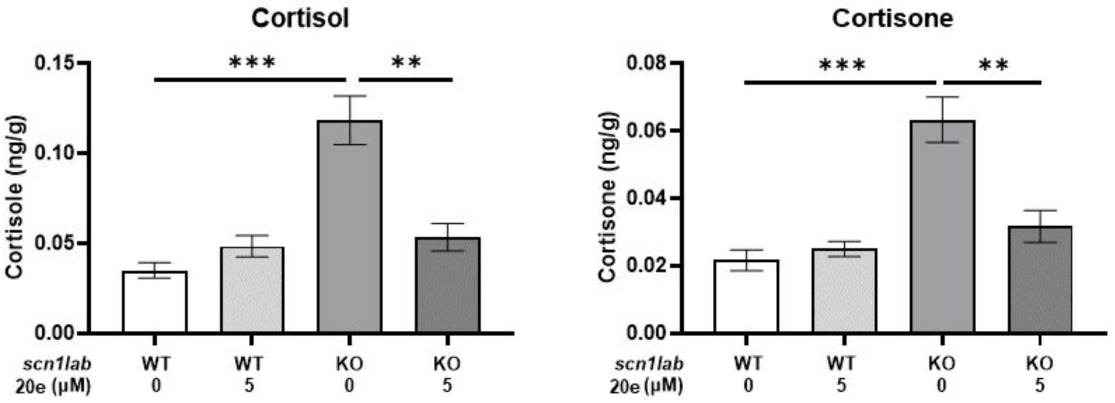

Supplementary Figure 4. Quantitative analysis of cortisol and cortisone in WT and *scn1lab* KO zebrafish larvae following compound 20e treatment.

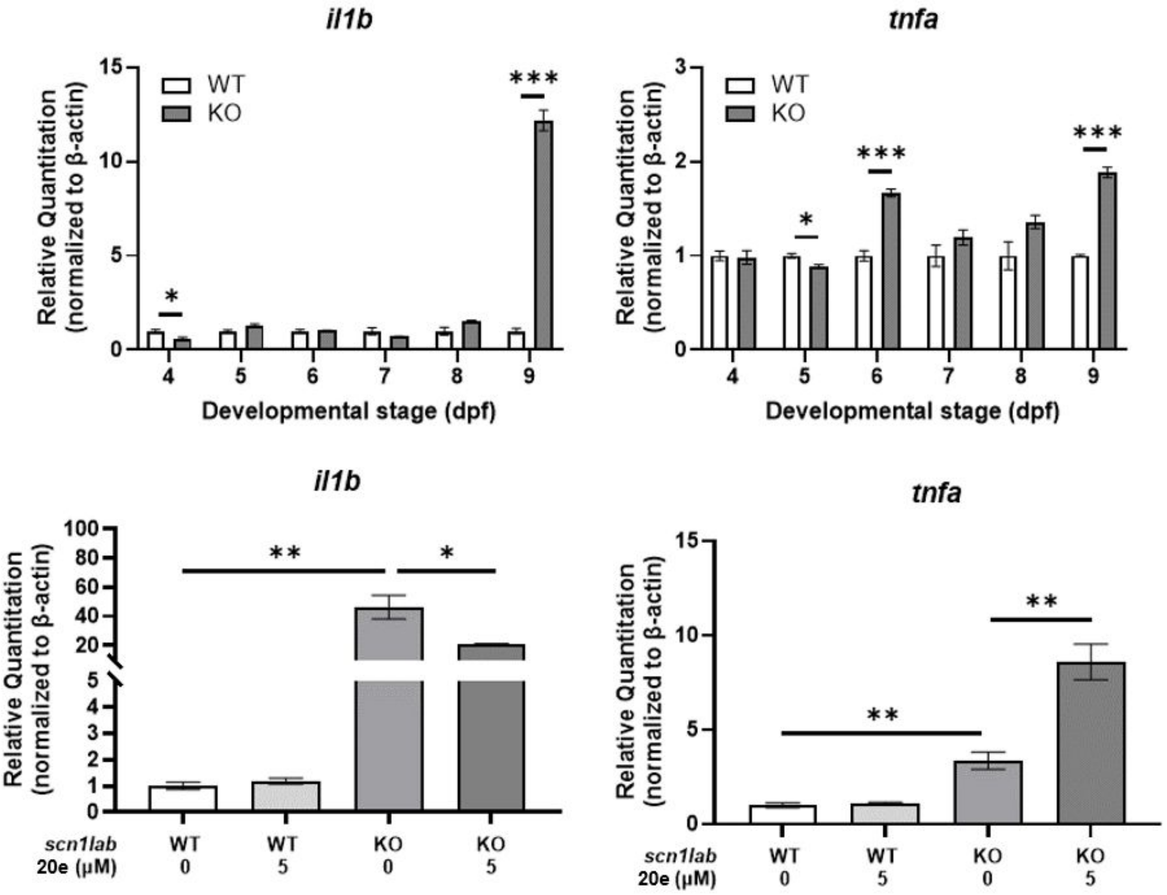

Supplementary Figure 5. Quantitative PCR analysis of *il1β* and *tnfa* expression in WT and *scn1lab* KO zebrafish larvae. KO larvae increased il1b expression at 9 dpf. Compound 20e significantly decreased *il1b* expression in KO larvae.

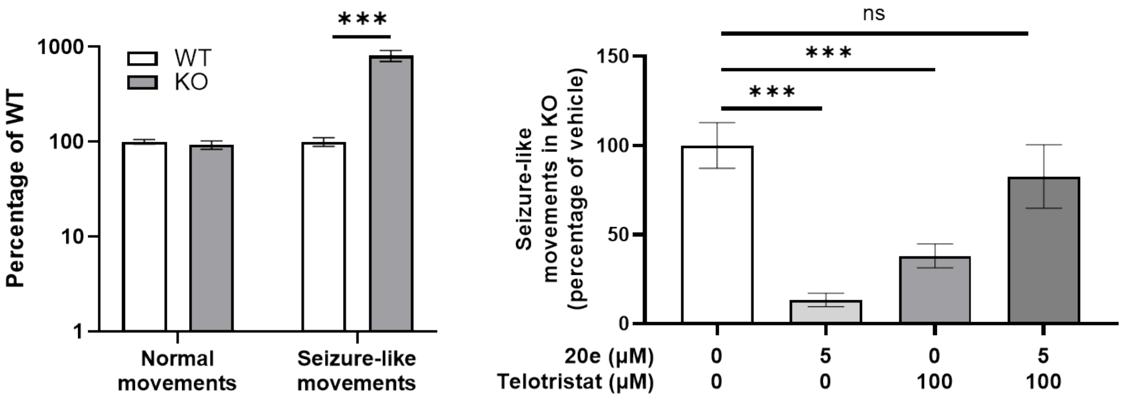

Supplementary Figure 6. Quantification of seizure-like movements in *scn1lab* KO zebrafish larvae following Telotristat and compound 20e treatment. Co-exposure of Telotristat, TPH1/2 inhibitor, and compound **20e** reduces the anti-seizure efficacy.

Chemistry

General Procedure of intermediate 4

Step 1. Commercially available thiophene-2-carbonitrile **2** (300.0 mg, 2.749 mmol) was dissolved in ethanol (10.0 mL). Hydroxylamine hydrochloride (286.50 mg, 4.123 mmol) and triethylamine were added. The reaction mixture was stirred under reflux for 8 h. After the reaction mixture was cooled to room temperature, the solvent was removed under reduced pressure. The residue was diluted with water and extracted with ethyl acetate (3×50 mL). Then the combined organic fraction was dried over anhydrous sodium sulfate, filtered. The solvent was removed under reduced pressure to give N-Hydroxy-2-thiophenecarboximidamide **3** (367.12 mg. 2.582 mmol, 94 %). ^{1}H NMR (400 MHz, chloroform-*d*) δ 8.67 (s, 1H), 7.32-7.28 (m, 2H), 7.04 (dd, J = 5.2, 3.7 Hz, 1H), 4.93 (s, 2H).

Step 2. N-Hydroxy-2-thiophenecarboximidamide **3** (367.12 mg, 2.582 mmol) was dissolved in THF (10 mL) and cooled to 0 ℃. Triphosgene (306.4 mg, 1.033 mmol) dissolved in THF was added slowly dropwise. After the mixture was stirred for 1h at 0 ℃, the reaction mixture was allowed to reach room temperature and heated to reflux for 6 h. After cooling to room temperature, the solvent was removed under reduced pressure. The residue was purified by silica gel column chromatography to give 3-(thiophen-2-yl)-1,2,4-oxadiazol-5(*4H*)-one **4** (311.26 mg, 1.851 mmol, 72 %). ^{1}H NMR (400 MHz, DMSO-d_6) δ 13.11 (s, 1H), 7.89 (dd, J = 5.2, 1.2 Hz, 1H), 7.67 (dd, J = 3.7, 1.2 Hz, 1H), 7.24 (dd, J = 5.0, 3.8 Hz, 1H).

General Procedure of intermediate 7

Charge an oven-dried 200-mL round-bottom flask, equipped with a stir bar, with m-CPBA (77 wt%, 546 mg, 2.43 mmol), 2-chlorophenylboronic acid **5** (2.11 mmol, 500 mg). Add DCM to the mixture under N2 atmosphere. Add BF3·OEt2 (4.219 mmol, 0.521 mL) to the mixture at room temperature. Allow the solution to stir at room temperature for 1 hour. Add (2-chlorophenyl)boronic acid **6** (2.321 mmol, 362.03 mg) to the mixture at 0°C. Stir the mixture for 15 minutes. Allow the mixture to stirred at room temperature for 1 hour. Add triflic acid (2.321 mmol, 0.205 mL) dropwise to the mixture at 0°C. Stir the mixture at room temperature for 30 minutes. Concentrate the mixture under reduced pressure. Add Et2O to the mixture. Stir the mixture the mixture at room temperature for 30 minutes to precipitate an off-white solid. Store the flask in a freezer for 1 hour. After 1 hour, filter off the solid. Wash the solid with cold Et2O. Dry the solid bis(2-chlorophenyl)iodonium trifluoromethanesulfonate **7** under vacuum (604 mg, 1.213 mmol, 58 %)[2].^{1}H-NMR (400 MHz, DMSO-~~D6~~d_6) δ 8.53 (d, J = 8.0 Hz, 1H), 7.85 (d, J = 7.6 Hz, 1H), 7.71 (t, J = 7.6 Hz, 1H), 7.50 (t, J = 7.6 Hz, 1H)

Procedure of derivative 8

4-(2-Chlorophenyl)-3-(thiophen-2-yl)-1,2,4-oxadiazol-5(*4H*)-one (8)

Add the solution of trimethylamine (0.446 mmol, 62 μL) in dried 1,2-dichloroethane to mixture of 3-(thiophen-2-yl)-1,2,4-oxadiazol-5(*4H*)-one (0.30 mmol, 50mg), bis(2-chlorophenyl)iodonium trifluoromethanesulfonate (0.3 mmol, 148 mg) and CuI (0.03 mmol, 5.56 mg) under Ar atmosphere. Heat resulted mixture at 60°C for 24 hours. Remove solvent under reduced pressure. The residue was purified by silica gel column chromatography to give 4-(2-chlorophenyl)-3-(thiophen-2-yl)-1,2,4-oxadiazol-5(*4H*)-one **8** as light brown. ^{1}H-NMR (400 MHz, DMSO-d_6) δ 7.95 (dd, J = 7.6, 1.5 Hz, 1H), 7.90 (dd, J = 5.0, 1.1 Hz, 1H), 7.80 (dd, J = 7.9, 1.2 Hz, 1H), 7.71 (td, J = 7.8, 1.5 Hz, 1H), 7.63 (td, J = 7.6, 1.4 Hz, 1H), 7.15-7.13 (m, 1H), 7.03 (dd, J = 3.7, 0.9 Hz, 1H); ^{13}C-NMR (101 MHz, DMSO-d_6) δ 157.8, 154.2, 134.1, 133.4, 133.3, 132.7, 131.6, 131.3, 130.1, 129.6, 129.3, 123.2, 41.1, 40.9, 40.7, 40.4, 40.2, 40.0, 39.8; HRMS (FAB) m/z calculated for $C_{12}H_7ClN_2O_2S$ $[M]^+$ 277.9917, found 277.9919; HPLC purity 97.8312%

General procedure of derivatives of 1, 10

4-(2-Chlorobenzyl)-3-(thiophen-2-yl)-1,2,4-oxadiazol-5(*4H*)-one (1)

3-(Thiophen-2-yl)-1,2,4-oxadiazol-5(*4H*)-one (50 mg, 0.297 mmol) and 2-chloro benzyl bromides **9a** (67.20 mg, 0.327 mmol) were dissolved in DMF (2 mL) under N_2 condition. Sodium hydride (7.85 mg, 0.327 mmol) in DMF was added slowly dropwise to the solution. The solution was heated to 60 °C and stirred for 4 h. The solvent was removed under reduced pressure. The residue was diluted with water (20 mL) and extracted with ethyl acetate (3×50 mL). The combined organic layer was washed with brine (20 mL). Crude product was purified by silica gel column chromatography to give 4-(2-chlorobenzyl)-3-(thiophen-2-yl)-1,2,4-oxadiazol-5(*4H*)-one **1** as white solid (63.5 mg, 0.217 mmol, 73 %). ^{1}H NMR (400 MHz, chloroform-*d*) δ 7.54 (d, J = 4.9 Hz, 1H), 7.41 (dd, J = 7.2, 1.7 Hz, 1H), 7.29-7.21 (m, 3H), 7.09-7.05 (m, 2H), 5.08 (s, 2H); ^{13}C-NMR (100 MHz, chloroform-*d*) δ 159.4, 154.3, 132.3, 131.7, 131.1, 130.5, 130.2, 129.8, 128.5, 127.7, 126.9, 122.6, 44.4; HRMS (FAB) m/z calculated for $C_{13}H_9ClN_2O_2S$ $[M]^+$ 292.0073, found 292.0074; HPLC purity 99.0191 %

4-(2-Chlorophenethyl)-3-(thiophen-2-yl)-1,2,4-oxadiazol-5(*4H*)-one (10)

3-(Thiophen-2-yl)-1,2,4-oxadiazol-5(*4H*)-one (50 mg, 0.297 mmol) and 1-(2-bromoethyl)-2-chlorobenzene **9b** (71.79 mg, 0.327 mmol) were dissolved in DMF (2 mL) under N_2 condition. Sodium hydride (60 % dispersion in mineral oil 14.29 mg, 0.357 mmol) in DMF was added slowly dropwise to the solution. The solution was heated to 60 °C and stirred for 4 h. The solvent was removed under reduced pressure. The residue was diluted with water (20 mL) and extracted with ethyl acetate (3×50 mL). The combined organic layer was washed with brine (20 mL). Crude product was purified by silica gel column chromatography to give 4-(2-chlorophenethyl)-3-(thiophen-2-yl)-1,2,4-oxadiazol-5(*4H*)-

one **10** as light brown solid (67.2 mg, 0.219 mmol, 74 %). ^{1}H-NMR (400 MHz, DMSO-~~D6~~*d_6*) δ 7.90 (d, J = 4.9 Hz, 1H), 7.53 (d, J = 3.4 Hz, 1H), 7.30-7.16 (m, 5H), 4.01 (t, J = 6.7 Hz, 2H), 2.99 (t, J = 6.7 Hz, 2H); ^{13}C-NMR (101 MHz, DMSO-~~D6~~*d_6*) δ 159.5, 155.0, 135.5, 134.1, 132.6, 132.4, 132.0, 130.3, 129.9, 129.4, 128.5, 122.9, 43.5, 41.1, 40.9, 40.7, 40.5, 40.2, 40.0, 39.8, 32.1; HRMS (FAB) m/z calculated for $C_{14}H_{11}ClN_2O_2S$ [M]$^+$ 306.0230, found 306.0233; HPLC purity 97.7090 %

4-Benzyl-3-(thiophen-2-yl)-1,2,4-oxadiazol-5(*4H*)-one (12a)

The procedure was shown in procedure of **4-(2-chlorobenzyl)-3-(thiophen-2-yl)-1,2,4-oxadiazol-5(*4H*)-one (1)**, but 2-chloro benzyl bromide were changed to benzyl bromide (55.94 mg. 0.327 mmol). 4-benzyl-3-(thiophen-2-yl)-1,2,4-oxadiazol-5(*4H*)-one **12a** was given as white solid (57.8 mg, 0.223 mmol, 75 %). ^{1}H NMR (400 MHz, chloroform-*d*) δ 7.58 (dd, J = 5.0, 1.1 Hz, 1H), 7.37-7.29 (m, *4H*), 7.23-7.18 (m, 2H), 7.12 (dd, J = 5.0, 3.8 Hz, 1H), 4.98 (s, 2H); ^{13}C-NMR (100 MHz, chloroform-*d*) δ 159.7, 154.3, 134.4, 131.0, 130.8, 129.3, 128.6, 128.4, 126.8, 122.8, 46.6; HRMS (FAB) m/z calculated for $C_{13}H_{10}N_2O_2S$ [M]$^+$ 258.0463, found 258.0468; HPLC purity 97.1025 %.

4-(2,4-Dichlorobenzyl)-3-(thiophen-2-yl)-1,2,4-oxadiazol-5(*4H*)-one (12b)

The procedure was shown in procedure of **4-(2-chlorobenzyl)-3-(thiophen-2-yl)-1,2,4-oxadiazol-5(*4H*)-one (1)**, but 2-chloro benzyl bromide were changed to 2,4-dichloro benzyl bromide (78.47 mg, 0.327 mmol). 4-(2,4-dichlorobenzyl)-3-(thiophen-2-yl)-1,2,4-oxadiazol-5(*4H*)-one **12b** was gotten as white solid (68.6 mg, 0.220 mmol, 74 %). ^{1}H NMR (400 MHz, chloroform-*d*) δ 7.59 (d, J = 4.9 Hz, 1H), 7.45 (d, J = 1.8 Hz, 1H), 7.27-7.25 (m, 2H), 7.13 (t, J = 4.4 Hz, 1H), 7.05 (d, J = 8.5 Hz, 1H), 5.06 (s, 2H); ^{13}C-NMR (100 MHz, chloroform-*d*) δ 159.3, 154.1, 135.1, 133.0, 131.2, 130.5, 130.4, 130.0, 128.5, 128.0, 128.0, 122.5, 44.0; HRMS (FAB) m/z calculated for $C_{13}H_8Cl_2N_2O_2S$ [M]$^+$ 325.9684, found 325.9682; HPLC purity 95.1924 %.

4-(2-Chloro-4-fluorobenzyl)-3-(thiophen-2-yl)-1,2,4-oxadiazol-5(*4H*)-one (12c)

The procedure was shown in step 3-1, but 2-chloro benzyl bromide were changed to 2-chloro-4-fluoro benzyl bromide (73.09 mg, 0.327 mmol). 4-(2-chloro-4-fluorobenzyl)-3-(thiophen-2-yl)-1,2,4-oxadiazol-5(*4H*)-one **12f** was provided as yellow solid (71.2 mg, 0.229 mmol, 77 %). ^{1}H NMR (400 MHz, chloroform-*d*) δ 7.59 (dd, J = 5.0, 1.1 Hz, 1H), 7.26 (dd, J = 3.7, 1.2 Hz, 1H), 7.20 (dd, J = 8.1, 2.6 Hz, 1H), 7.14-7.09 (m, 2H), 7.00 (td, J = 8.3, 2.5 Hz, 1H), 5.06 (s, 2H); ^{13}C-NMR (100 MHz, chloroform-*d*) δ 163.6, 161.1, 159.3, 154.1, 133.2, 133.1, 131.2, 130.5, 128.6, 128.5, 128.5, 127.8, 127.7,

122.5, 117.8, 117.6, 115.1, 114.9, 44.0; HRMS(FAB) m/z calculated for $C_{13}H_8ClFN_2O_2S$ $[M]^+$ 309.9979, found 309.9982; HPLC purity 99.0208 %.

4-(4-Fluorobenzyl)-3-(thiophen-2-yl)-1,2,4-oxadiazol-5(*4H*)-one (12d)

The procedure was shown in step 3-1, but 2-chloro benzyl bromide were changed to 4-fluoro benzyl bromide (61.82 mg, 0.327 mmol). 4-(4-fluorobenzyl)-3-(thiophen-2-yl)-1,2,4-oxadiazol-5(*4H*)-one **12d** was provided as white solid (61.00 mg, 0.224 mmol, 75 %). ^1H NMR (400 MHz, chloroform-*d*) δ 7.60 (dd, J = 5.0, 1.1 Hz, 1H), 7.34 (dd, J = 3.7, 1.2 Hz, 1H), 7.20-7.13 (m, 3H), 7.05-6.99 (m, 2H), 4.94 (s, 2H); ^{13}C-NMR (100 MHz, chloroform-*d*) δ 164.0, 161.5, 159.6, 154.0, 131.1, 130.8, 130.1, 129.0, 128.9, 128.4, 122.7, 116.4, 116.2, 46.0; HRMS (FAB) m/z calculated for $C_{13}H_9FN_2O_2S$ $[M]^+$ 276.0369, found 276.0370; HPLC purity 97.5188 %.

4-(2,4-Difluorobenzyl)-3-(thiophen-2-yl)-1,2,4-oxadiazol-5(*4H*)-one (12e)

The procedure was shown in procedure of **4-(2-chlorobenzyl)-3-(thiophen-2-yl)-1,2,4-oxadiazol-5(*4H*)-one (1)**, but 2-chloro benzyl bromide were changed to 2, 4-difluoro benzyl bromide (67.70 mg, 0.327 mmol). 4-(2,4-difluorobenzyl)-3-(thiophen-2-yl)-1,2,4-oxadiazol-5(*4H*)-one **12e** was provided as white solid (64.3 mg, 0.218 mmol, 74 %). ^1H NMR (400 MHz, chloroform-*d*) δ 7.61 (dd, J = 5.0, 1.1 Hz, 1H), 7.38 (dd, J = 3.8, 1.1 Hz, 1H), 7.24-7.16 (m, 2H), 6.90-6.81 (m, 2H), 5.01 (s, 2H); ^{13}C-NMR (100 MHz, chloroform-*d*) δ 164.4, 164.3, 161.9, 161.8, 161.5, 161.4, 159.4, 159.0, 154.0, 131.1, 130.6, 129.9, 129.9, 129.8, 129.8, 128.4, 122.4, 117.5, 117.3, 112.3, 112.3, 112.1, 112.1, 104.7, 104.5, 104.2, 40.4, 40.4; HRMS(FAB) m/z calculated for $C_{13}H_8F_2N_2O_2S$ $[M]^+$ 294.0275, found 264.0276; HPLC purity 98.0178 %.

4-(4-Chloro-2-fluorobenzyl)-3-(thiophen-2-yl)-1,2,4-oxadiazol-5(*4H*)-one (12f)

The procedure was shown in **4-(2-chlorobenzyl)-3-(thiophen-2-yl)-1,2,4-oxadiazol-5(*4H*)-one (1)**, but 2-chloro benzyl bromide were changed to 4-chloro-2-fluoro benzyl bromide (73.09 mg, 0.327 mmol). 4-(4-chloro-2-fluorobenzyl)-3-(thiophen-2-yl)-1,2,4-oxadiazol-5(*4H*)-one **12c** was provided as white solid (70.2 mg, 0.226 mmol, 76 %). ^1H NMR (400 MHz, chloroform-*d*) δ 7.61 (dd, J = 5.0, 1.1 Hz, 1H), 7.37 (dd, J = 3.8, 1.1 Hz, 1H), 7.18-7.11 (m, 4H), 5.01 (s, 2H); ^{13}C-NMR (100 MHz, chloroform-*d*) δ 163.6, 161.1, 159.4, 154.1, 133.2, 133.1, 131.2, 130.5, 128.5, 128.4, 127.7, 122.5, 117.9, 117.6, 115.1, 114.9, 43.9; HRMS(FAB) m/z calculated for $C_{13}H_8ClFN_2O_2S$ $[M]^+$ 309.9979, found 309.9982; HPLC purity 98.2010 %.

Procedure of compound 18

Step 1. The ethyl thiophene-2-carboxylate **13a** (500 mg, 3.201 mmol) was dissolved in ethanol. 4 equivalent of hydrazine hydrate was added in reaction mixture. The mixture was heated for reflux and stirred for 6 h. The solvent was removed under reduced pressure. The residue diluted by water and extracted with ethyl acetate. The organic phase was dried over anhydrous sodium sulfate and concentrated in vacuo to provide thiophene-2-carbohydrazide **14a** (326.4 mg, 2.296 mmol, 72 %). ^{1}H NMR (400 MHz, DMSO-d_6) δ 9.74 (s, 1H), 7.74-7.70 (m, 2H), 7.12 (t, J = 4.4 Hz, 1H), 4.45 (s, 2H).

Step 2. The solution of thiophene-2-carbohydrazide **14a** (200 mg, 1.407 mmol) in 10 mL 1,4-dioxane was treated with a solution of cyanogen bromide (149.0 mg, 1.407 mmol) in ACN and sodium hydrogen carbonate (88.69 mg, 2.110 mmol) in 5 mL water. The reaction mixture was stirred at room temperature for 16 h. The mixture was diluted by water and filtered to afford 5-(thiophen-2-yl)-1,3,4-oxadiazol-2-amine **15** (120.2 mg, 0.719 mmol, 51 %). ^{1}H NMR (400 MHz, chloroform-*d*) δ 7.58 (dd, J = 3.8, 1.1 Hz, 1H), 7.46 (dd, J = 4.9, 1.2 Hz, 1H), 7.12 (dd, J = 5.0, 3.8 Hz, 1H), 4.88 (s, 2H).

Step 3. The solution of 5-(thiophen-2-yl)-1,3,4-oxadiazol-2-amine **15** (120.2 mg, 0.719 mmol) in EtOH (20 mL) was treated potassium hydroxide (403.4 mg, 7.189 mmol) in ethanol. The mixture was heated to 85 °C for 16 h. After cooling to ambient temperature, the mixture was neutralized to pH 7 with 1 N HCl at 0 °C and then concentrated and then extracted with ethyl acetate (50 mL×3). The combined organic layers were dried with anhydrous sodium sulfate and concentrated. Crude product was purified by silica gel column chromatography to give 5-ethoxy-3-(thiophen-2-yl)-1H-1,2,4-triazole **16** (107.6 mg, 0.551 mmol, 77 %). ^{1}H NMR (400 MHz, chloroform-*d*) δ 7.60 (dd, J = 3.7, 1.2 Hz, 1H), 7.30 (dd, J = 4.9, 1.2 Hz, 1H), 7.14 (dd, J = 8.4, 2.6 Hz, 1H), 7.07-7.03 (m, 2H), 6.94 (td, J = 8.3, 2.7 Hz, 1H), 5.20 (s, 2H), 4.54 (q, J = 7.1 Hz, 2H), 1.42 (t, J = 7.2 Hz, 3H).

Step 4. 5-Ethoxy-3-(thiophen-2-yl)-1H-1,2,4-triazole **16** (107.6 mg, 0.551 mmol) and 2-chloro-4-fluoro benzyl bromides **10c** (135.45 mg, 0.606 mmol) in DMF (2 mL) under N_2. Sodium hydride (60 % dispersion in mineral oil 26.49 mg, 0.662 mmol) in DMF to the solution. The solution heated to 60 °C and stirred for 4 hours. The water was added to reaction mixture and extract with ethyl acetate (3×50 mL). The combined organic layer was washed with brine (20 mL). Crude product was purified by silica gel column chromatography to give 1-(2-chloro-4-fluorobenzyl)-5-ethoxy-3-(thiophen-2-yl)-1H-1,2,4-triazole **17** (127.7 mg, 0.378 mmol, 69 %). ^{1}H NMR (400 MHz, chloroform-*d*) δ 7.58 (dd, J = 3.7, 1.2 Hz, 1H), 7.30-7.27 (m, 1H), 7.13 (dd, J = 8.4, 2.6 Hz, 1H), 7.06-7.02 (m, 2H), 6.93 (td, J = 8.3, 2.6 Hz, 1H), 5.19 (s, 2H), 4.53 (q, J = 7.1 Hz, 2H), 1.41 (t, J = 7.2 Hz, 3H).

Step 5. 1-(2-Chloro-4-fluorobenzyl)-5-ethoxy-3-(thiophen-2-yl)-1H-1,2,4-triazole **17** (127.7 mg, 0.378 mmol) was dissolved in absolute ethanol (10 mL) and 5 mL concentrated HCl solution. The mixture was heated to reflux for 2 h. The mixture was cool to room temperature. The precipitate was isolated

by filtration, washed with sodium hydrogen carbonate solution and brine. The precipitate was dried over anhydrous sodium sulfate and concentrated in vacuo to provide 2-(2-chloro-4-fluorobenzyl)-5-(thiophen-2-yl)-2,4-dihydro-*3H*-1,2,4-triazol-3-one **18** (72.4 mg, 0.234 mmol, 62 %). ^{1}H NMR (400 MHz, DMSO-d_6) δ 7.63 (d, J = 4.0 Hz, 1H), 7.50 (d, J = 2.7 Hz, 1H), 7.45 (dd, J = 8.7, 2.6 Hz, 1H), 7.27-7.17 (m, 2H), 7.12 (dd, J = 4.9, 3.7 Hz, 1H), 4.91 (s, 2H); ^{13}C-NMR (100 MHz, DMSO-d_6) δ 163.1, 160.6, 154.6, 141.6, 133.4, 133.3, 131.7, 131.6, 131.2, 131.1, 129.4, 129.0, 128.5, 127.8, 117.4, 117.1, 115.3, 115.1, 45.3; HRMS (FAB) m/z calculated for $C_{13}H_9ClFN_3OS$ $[M]^+$ 309.0139, found 309.0136; HPLC purity 97.6346 %.

General procedure of 20a-d

Step 1. Aryl Hydrazide

The aryl ester was dissolved in ethanol. 4 equivalent of hydrazine hydrate was added in reaction mixture. The mixture was heated for reflux and stirred for 6 h. The solvent was removed under reduced pressure. The residue diluted by water and extracted with ethyl acetate. The organic phase was dried over anhydrous sodium sulfate and concentrated in vacuo to provide aryl hydrazide.

Step 1-2. Furan-2-carbohydrazide (14b)

Using the procedure step 1 with ethyl furan-2-carboxylateas as aryl ester **14a** (500 mg, 2.629 mmol) achieved furan-2-carbohydrazide **15a** (379.3 mg, 2.153 mmol, 82 %). ^{1}H NMR (400 MHz, DMSO-d_6) δ 9.61 (s, 1H), 7.80 (dd, J = 1.7, 0.8 Hz, 1H), 7.07 (dd, J = 3.5, 0.8 Hz, 1H), 6.59 (dd, J = 3.4, 1.8 Hz, 1H), 4.40 (s, 2H).

Step 1-3. Pyridine-2-carbohydrazide (14c)

Using the procedure step 1 with ethyl 2-pyridinecarboxylate as aryl ester **14c** (500 mg, 3.308 mmol) gained pyridine-2-carbohydrazide **15c** (432.9 mg, 3.157 mmol, 95 %). ^{1}H NMR (400 MHz, DMSO-d_6) δ 9.85 (s, 1H), 8.61 (dq, J = 4.7, 0.9 Hz, 1H), 7.99-7.95 (m, 2H), 7.59-7.54 (m, 1H), 4.55 (d, J = 4.3 Hz, 2H).

Step 1-4. Pyridine-4-carbohydrazide (14d)

Using the procedure step 1 with methyl pyridine-4-carbohylate as aryl ester **13d** (500 mg, 3.646 mmol) acquired pyridine-4-carbohydrazide **14d** (483.2 mg, 3.809 mmol, 96 %). ^{1}H NMR (400 MHz, DMSO-d_6) δ 10.08 (s, 1H), 8.70 (dd, J = 4.3, 1.5 Hz, 2H), 7.72 (dd, J = 4.4, 1.7 Hz, 2H), 4.61 (s, 2H).

Step 2. Aryl-1,3,4-oxadiazol-2(*3H*)-one

The aryl hydrazide and 2 equivalent N,N-Diisopropylethylamine dissolved in THF (10 mL) at 0 °C. Triphosgene dissolved in THF was added slowly dropwise. After the mixture was stirred for 1 h at 0 ℃, the reaction mixture was allowed to reach room temperature and heated to reflux for 6 h. After cooling to room temperature, the solvent was removed under reduced pressure. The residue was purified by silica gel column chromatography to give product.

Step 2-1. 5-(Thiophen-2-yl)-1,3,4-oxadiazol-2(*3H*)-one (19a)

Using the procedure step 2 with thiophene-2-carbohydrazide **14a** aryl hydarzide **7** (326.4 mg, 2.296 mmol) achieved 5-(thiophen-2-yl)-1,3,4-oxadiazol-2(*3H*)-one **8** (279.4 mg, 1.661 mmol, 72 %). ^{1}H NMR (400 MHz, chloroform-*d*) δ 9.55 (s, 1H), 7.62 (dd, J = 3.8, 1.1 Hz, 1H), 7.52 (dd, J = 4.9, 1.2 Hz, 1H), 7.14 (dd, J = 4.9, 3.7 Hz, 1H).

Step 2-2. 5-(Furan-2-yl)-1,3,4-oxadiazol-2(*3H*)-one (19b)

Using the procedure step 2 with furan-2-carbohydrazide as aryl hydrazide **14b** (379.3 mg, 2.153 mmol) achieved 5-(furan-2-yl)-1,3,4-oxadiazol-2(*3H*)-one **19b** (216.4 mg, 1.422 mmol, 66 %). ^{1}H NMR (400 MHz, chloroform-*d*) δ 9.46 (s, 1H), 7.60 (dd, J = 2.3, 1.1 Hz, 1H), 7.00 (dd, J = 3.7, 0.6 Hz, 1H), 6.56 (dd, J = 3.5, 1.7 Hz, 1H).

Step 2-3. 5-(Pyridin-2-yl)-1,3,4-oxadiazol-2(*3H*)-one (19c)

Using the procedure step 2 with pyridine-2-carbohydrazide as aryl hydrazide **14c** (432.9 mg, 3.157 mmol) gained 5-(pyridin-2-yl)-1,3,4-oxadiazol-2(*3H*)-one **19c** (384.4 mg, 2.356 mmol, 75 %). ^{1}H NMR (400 MHz, DMSO-d_6) δ 8.71 (dq, J = 4.7, 0.9 Hz, 2H), 7.99 (td, J = 7.8, 1.8 Hz, 2H), 7.91 (dt, J = 7.9, 1.1 Hz, 2H), 7.57 (ddd, J = 7.5, 4.7, 1.2 Hz, 2H).

Step 2-4. Pyridine-4-carbohydrazide (19d)

Using the procedure step 2 with pyridine-4-carbohydrazide **13d** (483.2 mg, 3.809 mmol) give 5-(pyridin-4-yl)-1,3,4-oxadiazol-2(*3H*)-one **18d** (378.4 mg, 2.318 mmol, 66 %). ^{1}H NMR (400 MHz, DMSO-d_6) δ 12.89 (s, 1H), 8.76 (dd, J = 4.4, 1.7 Hz, 2H), 7.73 (dd, J = 4.4, 1.7 Hz, 2H).

Step 3. 3-(2-Chloro-4-fluorobenzyl)-5-(aryl)-1,3,4-oxadiazol-2(*3H*)-one

The aryl-1,3,4-oxadiazol-2(*3H*)-one and 1.1equivalent 2-chloro-4-fluoro benzyl bromide were dissolved in DMF (2 mL) under N_2 condition. 1.1 equivalent of sodium hydride in DMF was added slowly dropwise to the solution. The solution was heated to 60 °C and stirred for 4 h. The solvent was removed under reduced pressure. The residue was diluted with water (20 mL) and extracted with ethyl acetate (3×50 mL). The combined organic layer was washed with brine (20 mL). Crude product was purified by silica gel column chromatography to give 3-(2-chloro-4-fluorobenzyl)-5-(aryl)-1,3,4-oxadiazol-2(*3H*)-one.

Step 3-1. 3-(2-Chloro-4-fluorobenzyl)-5-(thiophen-2-yl)-1,3,4-oxadiazol-2(*3H*)-one (20a)

Using the procedure step 3 with 5-(thiophen-2-yl)-1,3,4-oxadiazol-2(*3H*)-one **19a** as aryl-1,3,4-oxadiazol-2(*3H*)-one (279.4 mg, 1.661 mmol) got 3-(2-chloro-4-fluorobenzyl)-5-(thiophen-2-yl)-1,3,4-oxadiazol-2(*3H*)-one **20a** (339.5 mg, 1.093 mmol, 66 %). ^{1}H NMR (400 MHz, chloroform-*d*) δ 7.58 (dd, J = 3.7, 1.2 Hz, 1H), 7.49 (dd, J = 5.2, 1.2 Hz, 1H), 7.36 (dd, J = 8.7, 6.0 Hz, 1H), 7.16 (dd, J = 8.4, 2.6 Hz, 1H), 7.11 (dd, J = 5.0, 3.8 Hz, 1H), 7.00 (td, J = 8.2, 2.6 Hz, 1H), 5.03 (s, 2H); ^{13}C-NMR (100 MHz, chloroform-*d*) δ 163.7, 161.2, 152.9, 150.5, 134.5, 134.4, 131.4, 131.4, 129.9, 129.5, 128.5, 128.4, 128.1, 125.3, 117.5, 117.2, 114.6, 114.4, 46.8; HRMS (FAB) m/z calculated for $C_{13}H_8ClFN_2O_2S$ $[M]^+$ 309.9979, found 309.9976; HPLC purity 98.1227 %.

Step 3-2. 3-(2-Chloro-4-fluorobenzyl)-5-(furan-2-yl)-1,3,4-oxadiazol-2(*3H*)-one (20b)

Using the procedure step 3 with 5-(furan-2-yl)-1,3,4-oxadiazol-2(*3H*)-one **19a** as aryl-1,3,4-boxadiazol-2(*3H*)-one (216.4 mg, 1.422 mmol) achieved 3-(2-chloro-4-fluorobenzyl)-5-(furan-2-yl)-1,3,4-oxadiazol-2(*3H*)-one **20b** (256.5 mg, 0.87 mmol, 61 %). ^{1}H NMR (400 MHz, chloroform-*d*) δ 7.56 (q, J = 0.9 Hz, 1H), 7.37 (dd, J = 8.5, 5.8 Hz, 1H), 7.16 (dd, J = 8.4, 2.6 Hz, 1H), 7.02-6.96 (m, 2H), 6.53 (dd, J = 3.7, 1.8 Hz, 1H), 5.03 (s, 2H); ^{13}C-NMR (100 MHz, chloroform-*d*) δ 163.7, 161.2, 152.5, 147.1, 145.8, 138.9, 134.6, 134.5, 131.7, 131.6, 128.3, 128.3, 117.5, 117.2, 114.7, 114.4, 114.0, 112.1, 46.9; HRMS (FAB) m/z calculated for $C_{13}H_8ClFN_2O_3$ $[M]^+$ 294.0208, found 294.0209; HPLC purity 97.0987 %.

Step 3-3. 3-(2-Chloro-4-fluorobenzyl)-5-(pyridin-2-yl)-1,3,4-oxadiazol-2(*3H*)-one (20c)

Using the procedure step 3 with 5-(pyridin-2-yl)-1,3,4-oxadiazol-2(*3H*)-one **19c** as aryl-1,3,4-oxadiazol-2(*3H*)-one (384.4 mg, 2.356 mmol) gained 3-(2-chloro-4-fluorobenzyl)-5-(pyridin-2-yl)-1,3,4-oxadiazol-2(*3H*)-one **20c** (413.8 mg, 1.354 mmol, 57 %). ^{1}H NMR (400 MHz, chloroform-*d*) δ 8.74 (td, J = 3.1, 1.7 Hz, 1H), 7.87-7.82 (m, 2H), 7.52-7.39 (m, 2H), 7.17 (dd, J = 8.4, 2.6 Hz, 1H), 7.00 (td, J = 8.3, 2.5 Hz, 1H), 5.11 (s, 2H); ^{13}C-NMR (100 MHz, chloroform-*d*) δ 163.7, 161.2, 153.1, 152.6, 150.6, 143.0, 137.3, 134.6, 134.5, 131.7, 131.6, 128.3, 128.3, 125.9, 121.9, 117.5, 117.2, 114.6, 114.4,

47.0; HRMS(FAB) m/z calculated for $C_{14}H_9ClFN_3O_2$ $[M]^+$ 305.0367, found 305.0365; HPLC purity 97.8957 %.

Step 3-4. 3-(2-Chloro-4-fluorobenzyl)-5-(pyridin-4-yl)-1,3,4-oxadiazol-2(*3H*)-one (20d)

Using the procedure step 3 with 5-(pyridin-4-yl)-1,3,4-oxadiazol-2(*3H*)-one **19d** (378.4 mg, 2.318 mmol) give 3-(2-chloro-4-fluorobenzyl)-5-(pyridin-4-yl)-1,3,4-oxadiazol-2(*3H*)-one **20d** (463.7 mg, 1.517 mmol, 65 %). ^{1}H NMR (400 MHz, chloroform-*d*) δ 8.76 (dd, J = 4.4, 1.7 Hz, 2H), 7.67 (dd, J = 4.4, 1.7 Hz, 2H), 7.40 (dd, J = 8.5, 5.8 Hz, 1H), 7.19 (dd, J = 8.2, 2.7 Hz, 1H), 7.03 (td, J = 8.2, 2.7 Hz, 1H), 5.09 (s, 2H); ^{13}C-NMR (100 MHz, chloroform-*d*) δ 163.8, 161.3, 152.8, 151.7, 150.8, 134.8, 134.7, 131.9, 131.8, 131.0, 128.1, 128.1, 119.2, 117.6, 117.4, 114.7, 114.5, 47.0; HRMS(FAB) m/z calculated for $C_{14}H_9ClFN_3O_2$ $[M]^+$ 305.0367, found 305.0369; HPLC purity 97.8644 %.

HPLC purity analysis

4-(2-Chlorobenzyl)-3-(thiophen-2-yl)-1,2,4-oxadiazol-5(*4H*)-one (1)

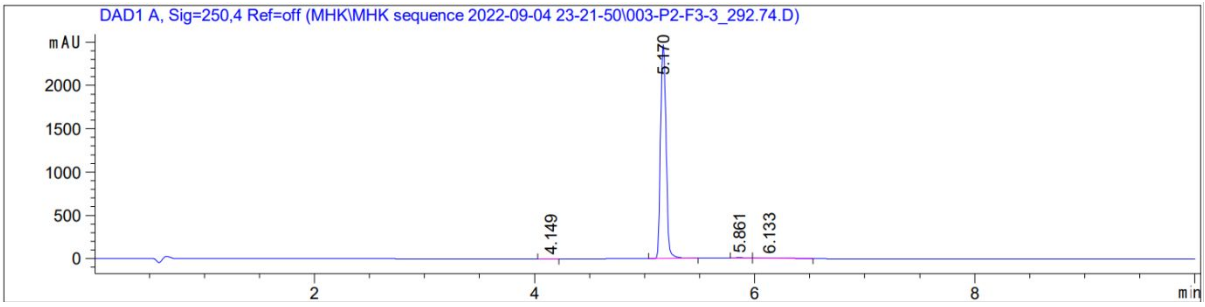

Signal 1: DAD1 A, Sig=250,4 Ref=off

Peak #	RetTime [min]	Type	Width [min]	Area [mAU*s]	Height [mAU]	Area %
1	4.149	BB	0.0686	5.65356	1.25429	0.0652
2	5.170	BB	0.0565	8586.09082	2473.85864	99.0191
3	5.861	BB	0.0484	26.94418	8.62668	0.3107
4	6.133	BB	0.2179	52.46173	3.00107	0.6050
Totals :				8671.15030	2486.74067	

4-(2-Chlorophenyl)-3-(thiophen-2-yl)-1,2,4-oxadiazol-5(*4H*)-one (8)

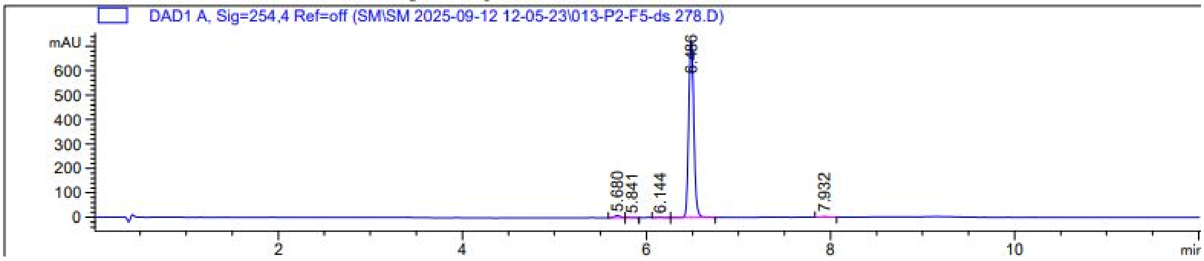

Signal 1: DAD1 A, Sig=254,4 Ref=off

Peak #	RetTime [min]	Type	Width [min]	Area [mAU*s]	Height [mAU]	Area %
1	5.680	BB	0.0568	34.87425	9.49924	1.2995
2	5.841	BB	0.0680	6.24223	1.29947	0.2326
3	6.144	BB	0.0702	7.18674	1.49150	0.2678
4	6.486	BB	0.0566	2625.40234	718.39886	97.8312
5	7.932	BB	0.0626	9.89876	2.37541	0.3689
Totals :				2683.60432	733.06448	

4-(2-Chlorophenethyl)-3-(thiophen-2-yl)-1,2,4-oxadiazol-5(*4H*)-one (10)

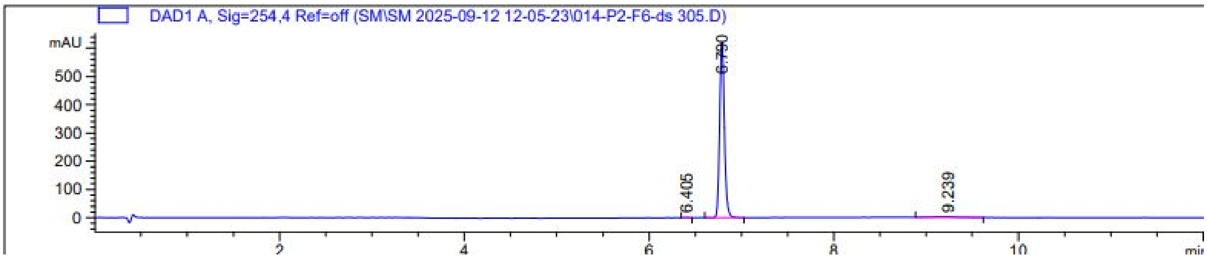

Signal 1: DAD1 A, Sig=254,4 Ref=off

Peak #	RetTime [min]	Type	Width [min]	Area [mAU*s]	Height [mAU]	Area %
1	6.405	BB	0.0532	5.69248	1.78403	0.2446
2	6.790	BB	0.0566	2273.70532	622.09125	97.7090
3	9.239	BB	0.2479	47.61887	2.39558	2.0463
Totals :				2327.01667	626.27086	

4-Benzyl-3-(thiophen-2-yl)-1,2,4-oxadiazol-5(*4H*)-one (12a)

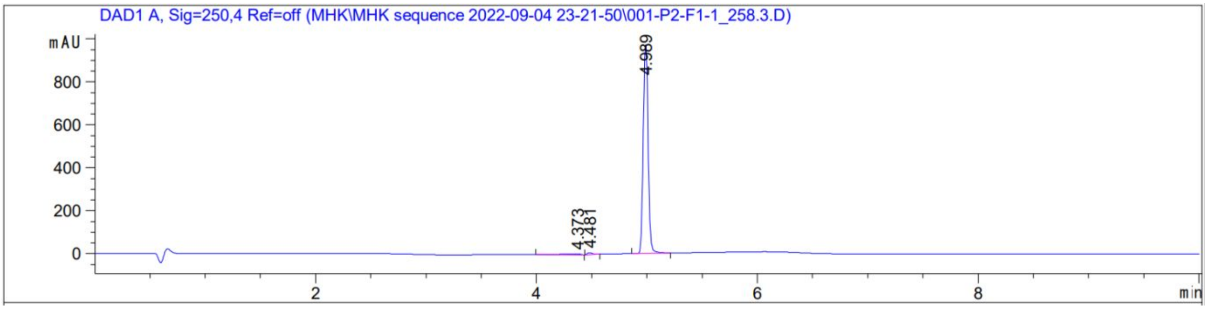

Signal 1: DAD1 A, Sig=250,4 Ref=off

Signal 1: DAD1 A, Sig=254,4 Ref=off

Peak #	RetTime [min]	Type	Width [min]	Area [mAU*s]	Height [mAU]	Area %
1	5.680	BB	0.0568	34.87425	9.49924	1.2995
2	5.841	BB	0.0680	6.24223	1.29947	0.2326
3	6.144	BB	0.0702	7.18674	1.49150	0.2678
4	6.486	BB	0.0566	2625.40234	718.39886	97.8312
5	7.932	BB	0.0626	9.89876	2.37541	0.3689
Totals :				2683.60432	733.06448	

4-(2,4-Dichlorobenzyl)-3-(thiophen-2-yl)-1,2,4-oxadiazol-5(*4H*)-one (12b)

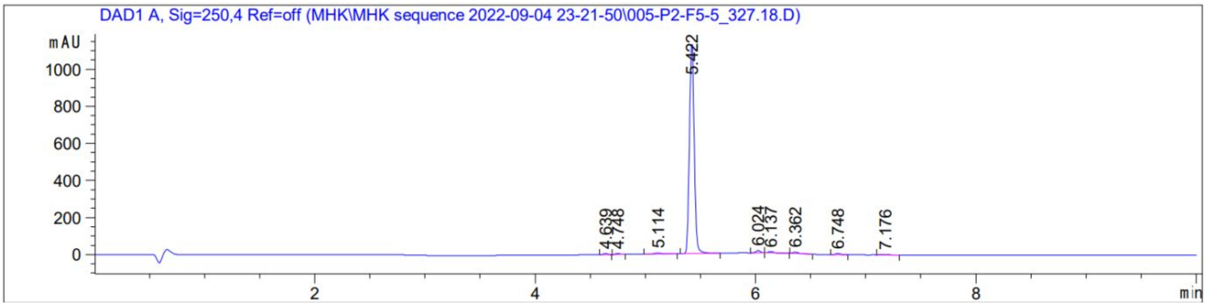

Signal 1: DAD1 A, Sig=250,4 Ref=off

Peak #	RetTime [min]	Type	Width [min]	Area [mAU*s]	Height [mAU]	Area %
1	4.639	BB	0.0481	16.80563	5.74850	0.4621
2	4.748	BB	0.0469	15.45229	5.16030	0.4249
3	5.114	BB	0.0716	26.57530	5.38211	0.7307
4	5.422	BB	0.0474	3462.18359	1140.50159	95.1924
5	6.024	BB	0.0460	32.65572	11.22108	0.8979
6	6.137	BB	0.0531	25.52765	7.62640	0.7019
7	6.362	BB	0.0537	31.80807	8.90557	0.8746
8	6.748	BB	0.0478	20.64533	6.73681	0.5676
9	7.176	BB	0.0600	5.38387	1.36547	0.1480
Totals :				3637.03746	1192.64783	

4-(2-Chloro-4-fluorobenzyl)-3-(thiophen-2-yl)-1,2,4-oxadiazol-5(*4H*)-one (12c)

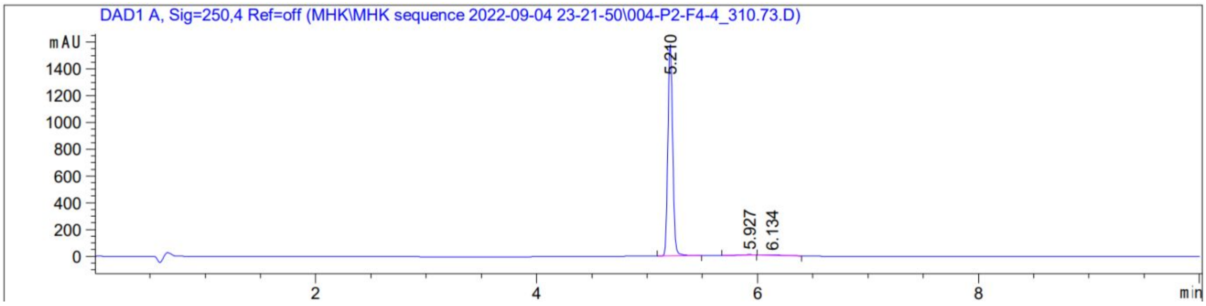

Signal 1: DAD1 A, Sig=250,4 Ref=off

Peak #	RetTime [min]	Type	Width [min]	Area [mAU*s]	Height [mAU]	Area %
1	5.210	BB	0.0498	4846.75195	1578.72949	99.0208
2	5.927	BB	0.0490	17.89711	5.63570	0.3656
3	6.134	BB	0.1281	30.03273	3.07203	0.6136
Totals :				4894.68180	1587.43723	

4-(4-Fluorobenzyl)-3-(thiophen-2-yl)-1,2,4-oxadiazol-5(*4H*)-one (12d)

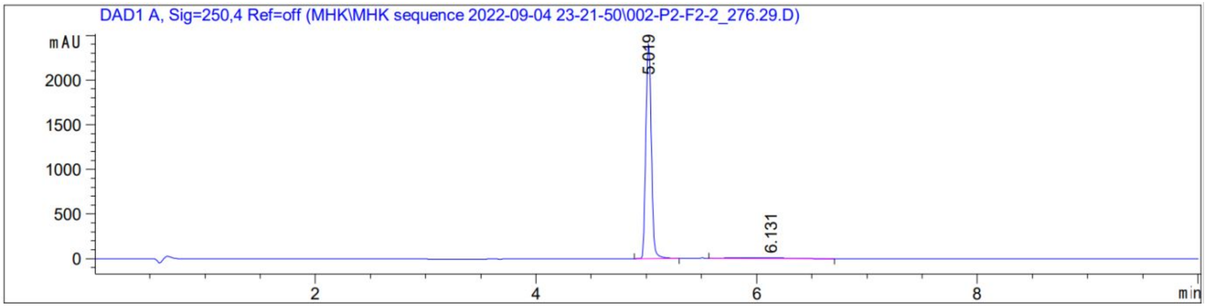

Signal 1: DAD1 A, Sig=250,4 Ref=off

Peak #	RetTime [min]	Type	Width [min]	Area [mAU*s]	Height [mAU]	Area %
1	5.019	BB	0.0564	8338.28223	2408.53589	97.5188
2	6.131	BB	0.3594	212.15205	7.20768	2.4812

Totals : 8550.43428 2415.74356

4-(2,4-Difluorobenzyl)-3-(thiophen-2-yl)-1,2,4-oxadiazol-5(*4H*)-one (12e)

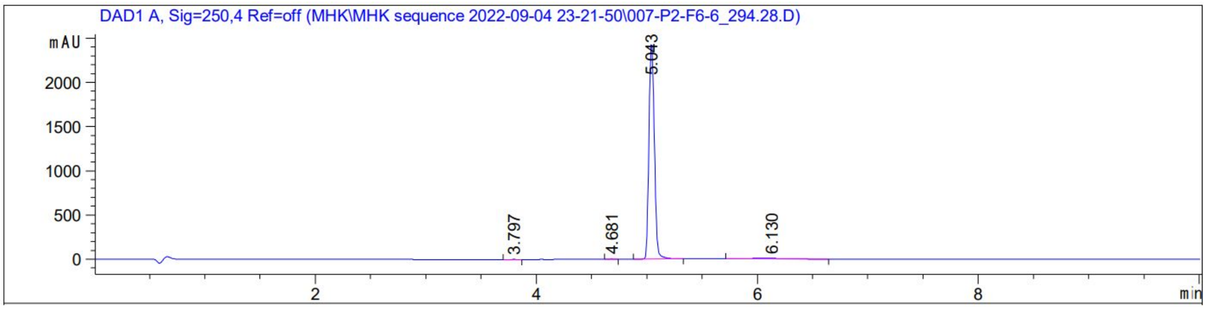

Signal 1: DAD1 A, Sig=250,4 Ref=off

Peak #	RetTime [min]	Type	Width [min]	Area [mAU*s]	Height [mAU]	Area %
1	3.797	BB	0.0615	5.36021	1.37444	0.0632
2	4.681	BB	0.0483	10.40802	3.53869	0.1227
3	5.043	BB	0.0557	8313.48340	2445.93994	98.0178
4	6.130	BB	0.3103	152.35695	5.95800	1.7963

Totals : 8481.60858 2456.81108

4-(4-Chloro-2-fluorobenzyl)-3-(thiophen-2-yl)-1,2,4-oxadiazol-5(*4H*)-one (12f)

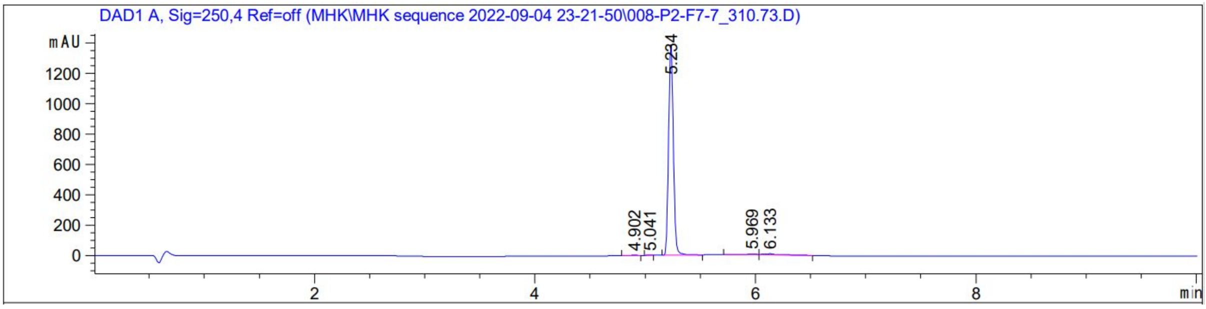

Signal 1: DAD1 A, Sig=250,4 Ref=off

Peak #	RetTime [min]	Type	Width [min]	Area [mAU*s]	Height [mAU]	Area %
1	4.902	BB	0.0496	11.31601	3.71049	0.2630
2	5.041	BB	0.0398	5.51520	2.31304	0.1282
3	5.234	BB	0.0495	4224.52393	1386.91260	98.2010
4	5.969	BB	0.0692	12.47423	2.84241	0.2900
5	6.133	BB	0.1106	48.08522	5.80894	1.1178
Totals :				4301.91458	1401.58747	

2-(2-Chloro-4-fluorobenzyl)-5-(thiophen-2-yl)-2,4-dihydro-*3H*-1,2,4-triazol-3-one (18)

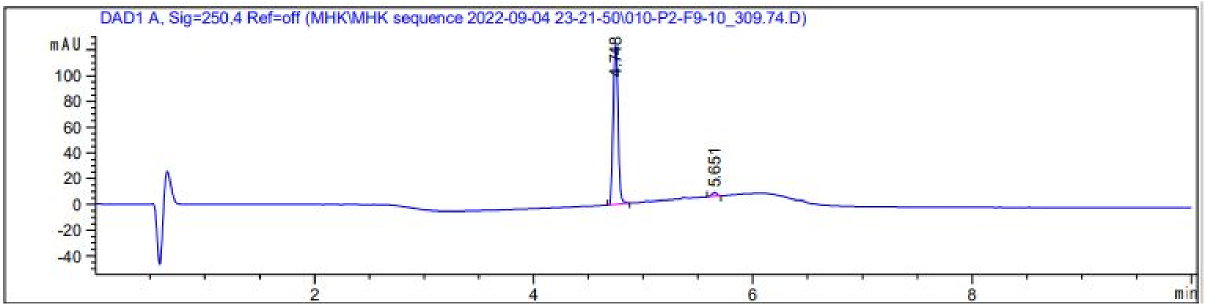

Signal 1: DAD1 A, Sig=250,4 Ref=off

Peak #	RetTime [min]	Type	Width [min]	Area [mAU*s]	Height [mAU]	Area %
1	4.748	BB	0.0496	373.82730	122.41949	97.6346
2	5.651	BB	0.0461	9.05682	3.09471	2.3654
Totals :				382.88412	125.51420	

3-(2-Chloro-4-fluorobenzyl)-5-(thiophen-2-yl)-1,3,4-oxadiazol-2(*3H*)-one (20a)

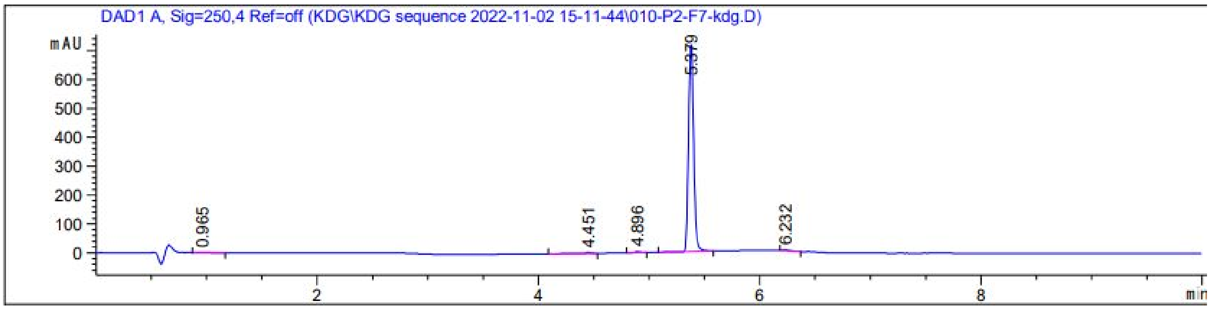

Signal 1: DAD1 A, Sig=250,4 Ref=off

Peak #	RetTime [min]	Type	Width [min]	Area [mAU*s]	Height [mAU]	Area %
1	0.965	BB	0.0960	7.16864	1.04559	0.3117
2	4.451	BB	0.0575	11.13254	2.98628	0.4841
3	4.896	BB	0.0539	14.17910	3.95260	0.6166
4	5.379	BB	0.0484	2256.37012	723.02478	98.1227
5	6.232	BB	0.0516	10.69012	3.15033	0.4649

Totals : 2299.54052 734.15959

3-(2-Chloro-4-fluorobenzyl)-5-(furan-2-yl)-1,3,4-oxadiazol-2(*3H*)-one (20b)

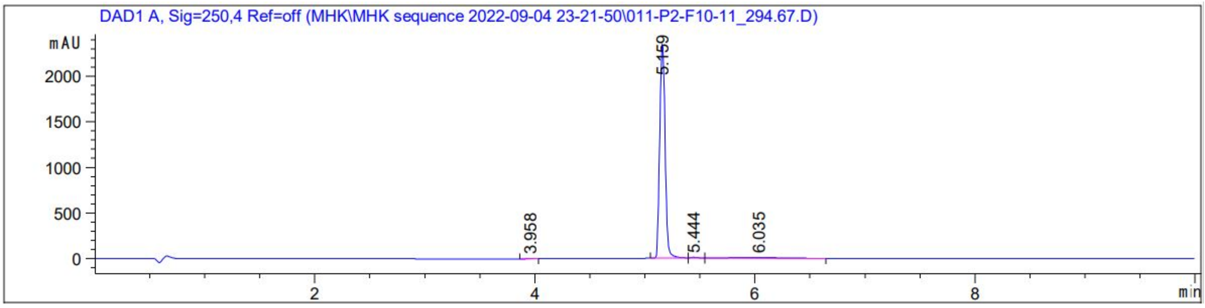

Signal 1: DAD1 A, Sig=250,4 Ref=off

Peak #	RetTime [min]	Type	Width [min]	Area [mAU*s]	Height [mAU]	Area %
1	3.958	BB	0.0546	14.87376	4.27868	0.1804
2	5.159	BB	0.0557	8004.14844	2353.76587	97.0987
3	5.444	BB	0.0505	20.09964	6.43299	0.2438
4	6.035	BB	0.4130	204.19269	5.94093	2.4771

Totals : 8243.31452 2370.41846

3-(2-Chloro-4-fluorobenzyl)-5-(pyridin-2-yl)-1,3,4-oxadiazol-2(*3H*)-one (20c)

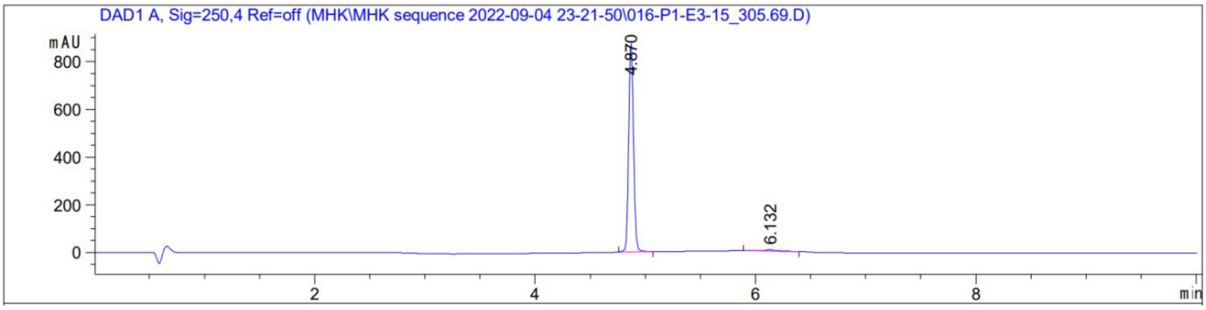

Signal 1: DAD1 A, Sig=250,4 Ref=off

Peak #	RetTime [min]	Type	Width [min]	Area [mAU*s]	Height [mAU]	Area %
1	4.870	BB	0.0481	2696.32031	871.92688	97.8957
2	6.132	BB	0.1244	57.95730	6.12575	2.1043

Totals : 2754.27761 878.05263

3-(2-Chloro-4-fluorobenzyl)-5-(pyridin-4-yl)-1,3,4-oxadiazol-2(*3H*)-one (20d)

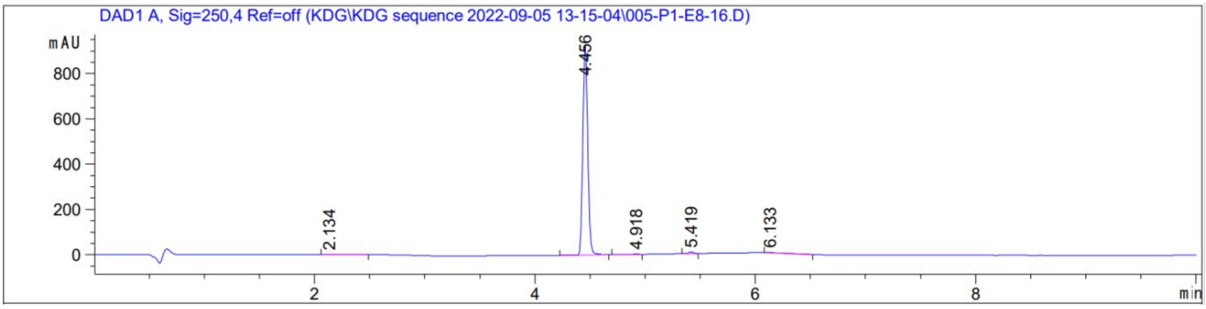

Signal 1: DAD1 A, Sig=250,4 Ref=off

Peak #	RetTime [min]	Type	Width [min]	Area [mAU*s]	Height [mAU]	Area %
1	2.134	BB	0.1605	19.51988	1.62122	0.6549
2	4.456	BB	0.0507	2917.06812	928.09601	97.8644
3	4.918	BB	0.0790	5.73563	1.06173	0.1924
4	5.419	BB	0.0482	20.68680	7.05893	0.6940
5	6.133	BB	0.1399	17.71411	1.64184	0.5943

Totals : 2980.72454 939.47973

3-(2-Chloro-4-fluorobenzyl)-5-(2-(trifluoromethyl)pyridin-4-yl)-1,3,4-oxadiazol-2(*3H*)-one (20e)

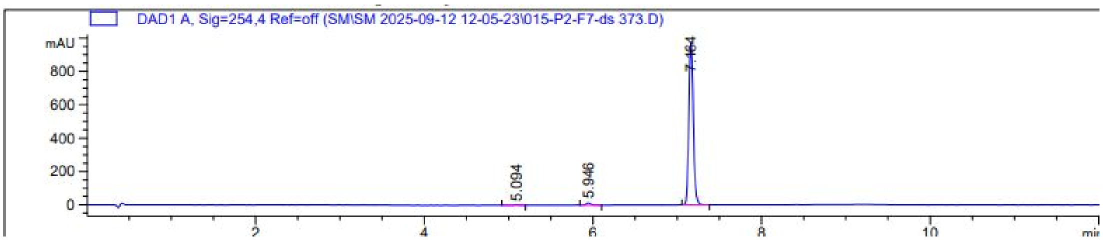

Signal 1: DAD1 A, Sig=254,4 Ref=off

Peak #	RetTime [min]	Type	Width [min]	Area [mAU*s]	Height [mAU]	Area %
1	5.094	BB	0.0702	12.81951	2.75762	0.3530
2	5.946	BB	0.0583	53.93753	14.19799	1.4854
3	7.164	BB	0.0569	3564.50195	970.44421	98.1616

Totals : 3631.25899 987.39983

^{1}H NMR and ^{13}C NMR spectra

4-(2-Chlorobenzyl)-3-(thiophen-2-yl)-1,2,4-oxadiazol-5(*4H*)-one (1)

^{1}H NMR, ^{13}C NMR

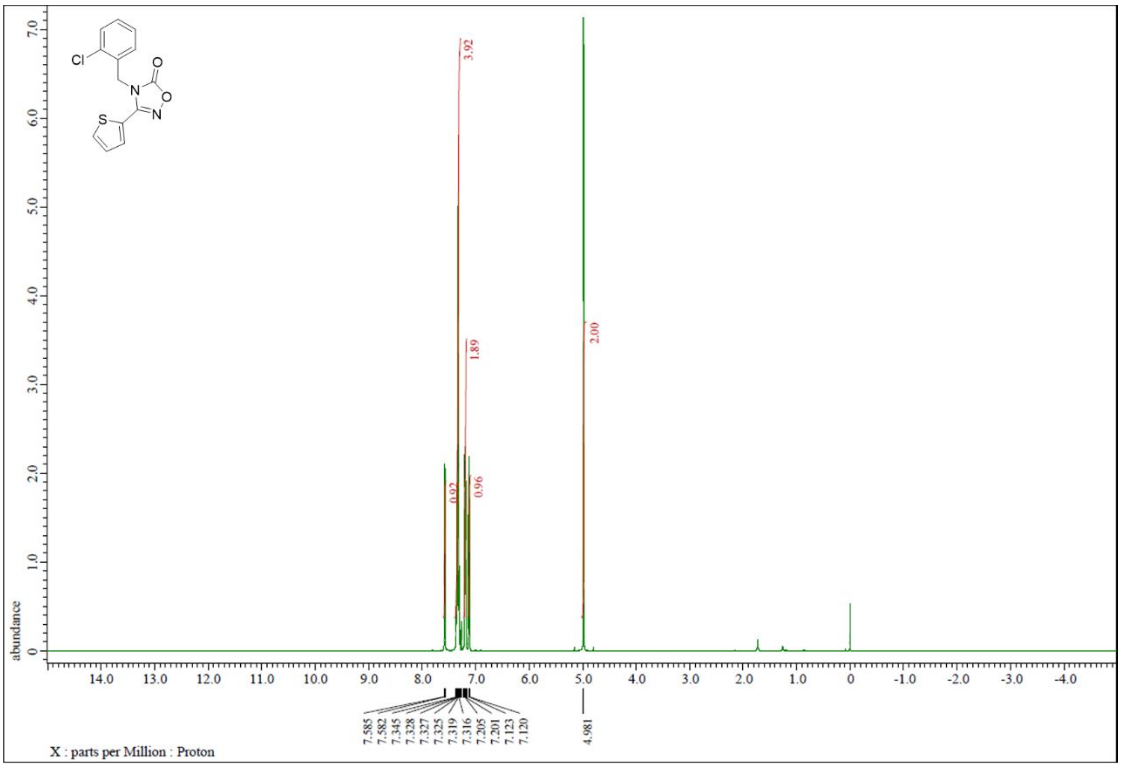

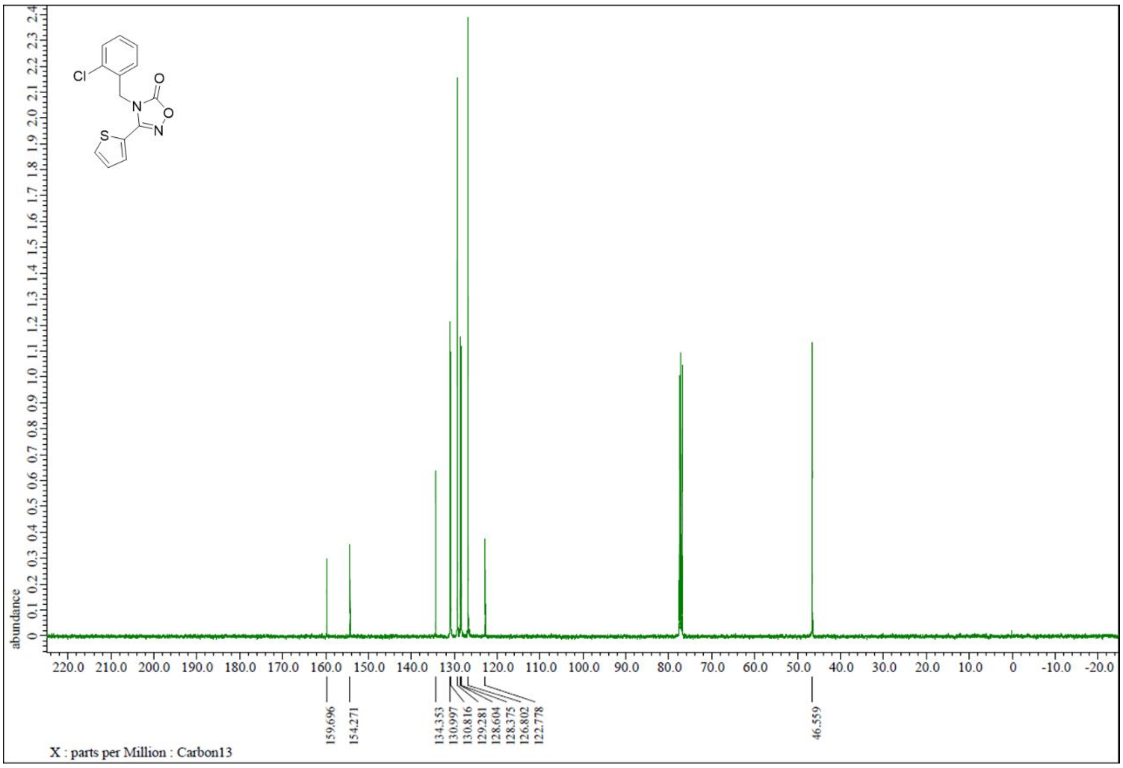

4-(2-Chlorophenyl)-3-(thiophen-2-yl)-1,2,4-oxadiazol-5(*4H*)-one (8)

^{1}H NMR, ^{13}C NMR

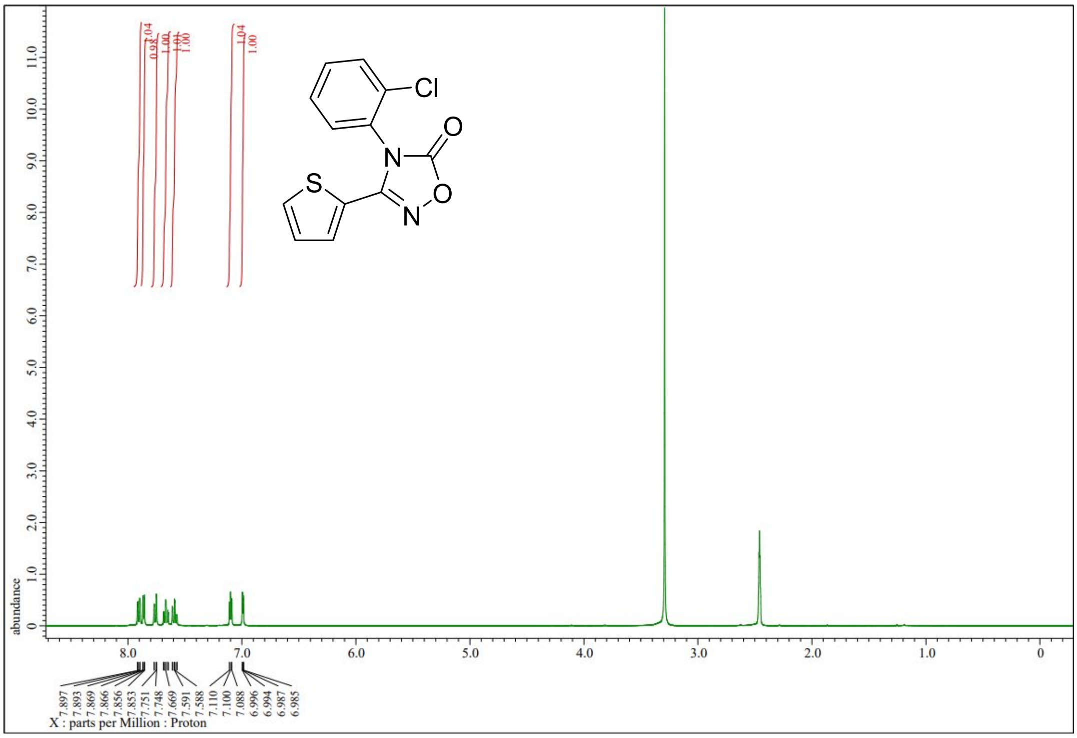

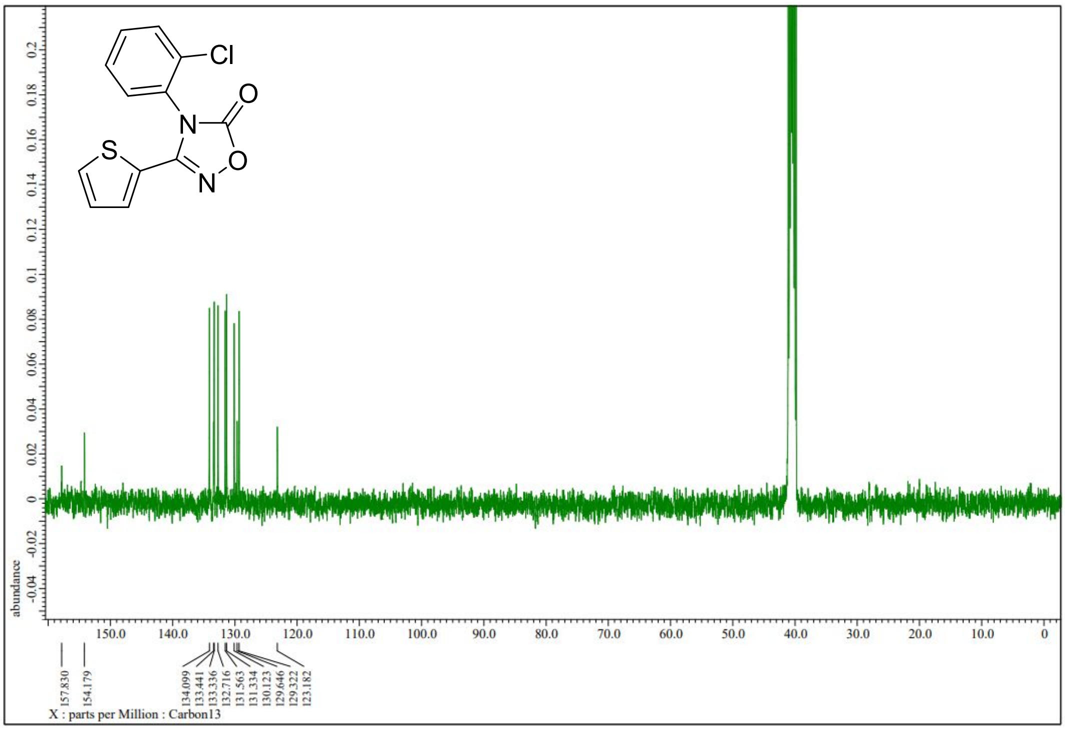

4-(2-Chlorophenethyl)-3-(thiophen-2-yl)-1,2,4-oxadiazol-5(*4H*)-one (10)

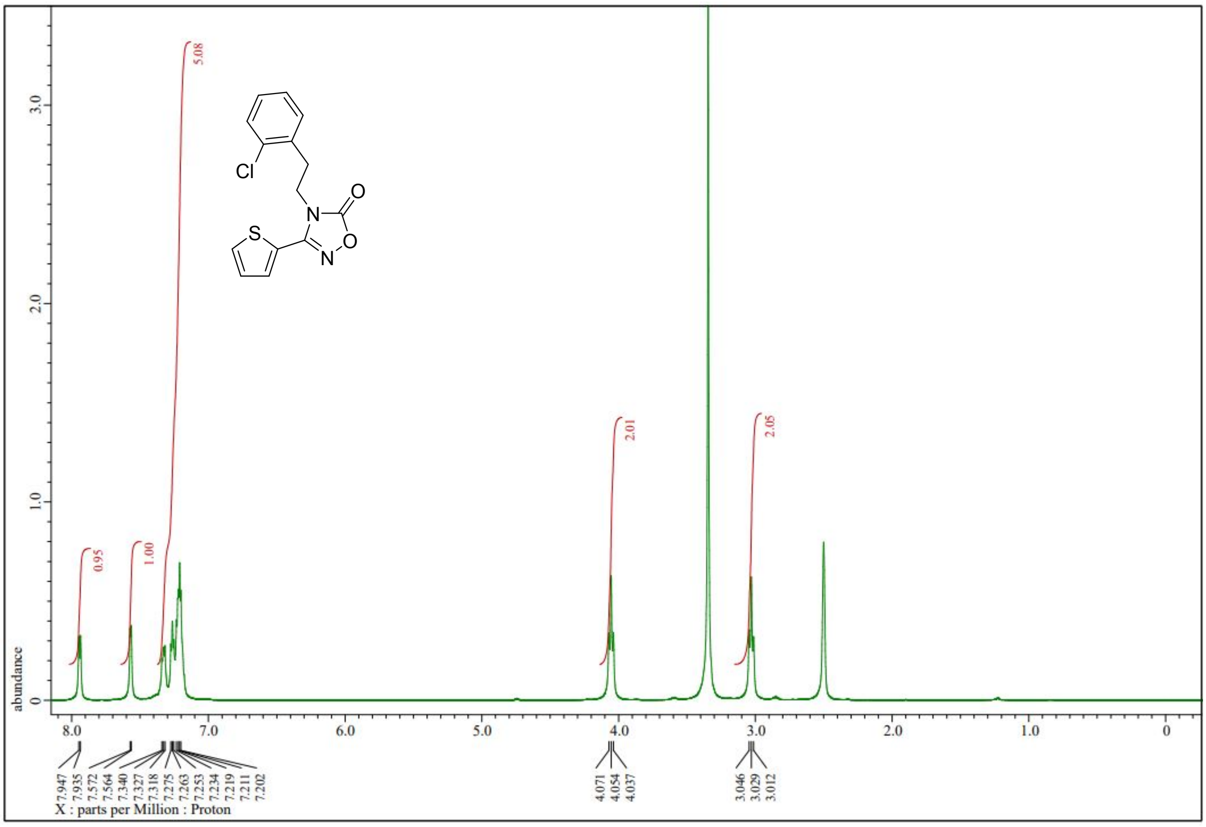

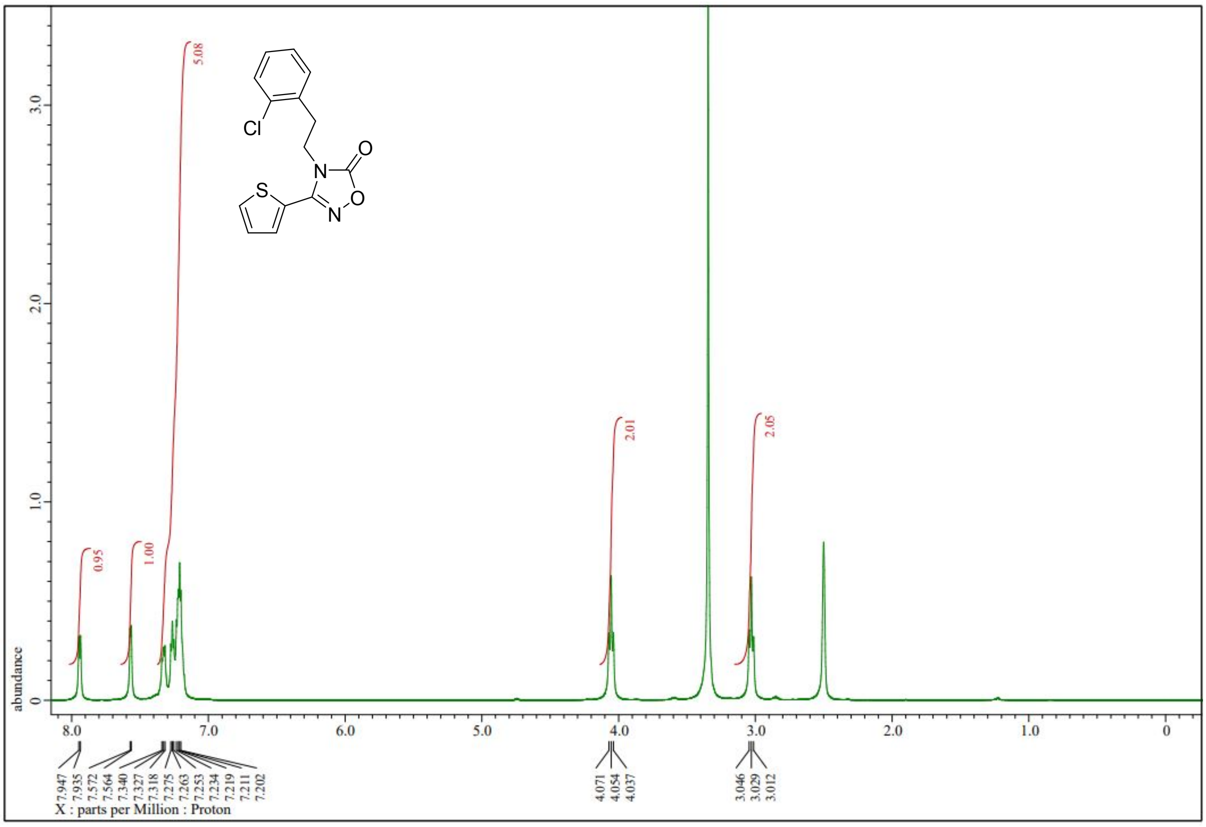

4-Benzyl-3-(thiophen-2-yl)-1,2,4-oxadiazol-5(*4H*)-one (12a)

^{1}H NMR, ^{13}C NMR

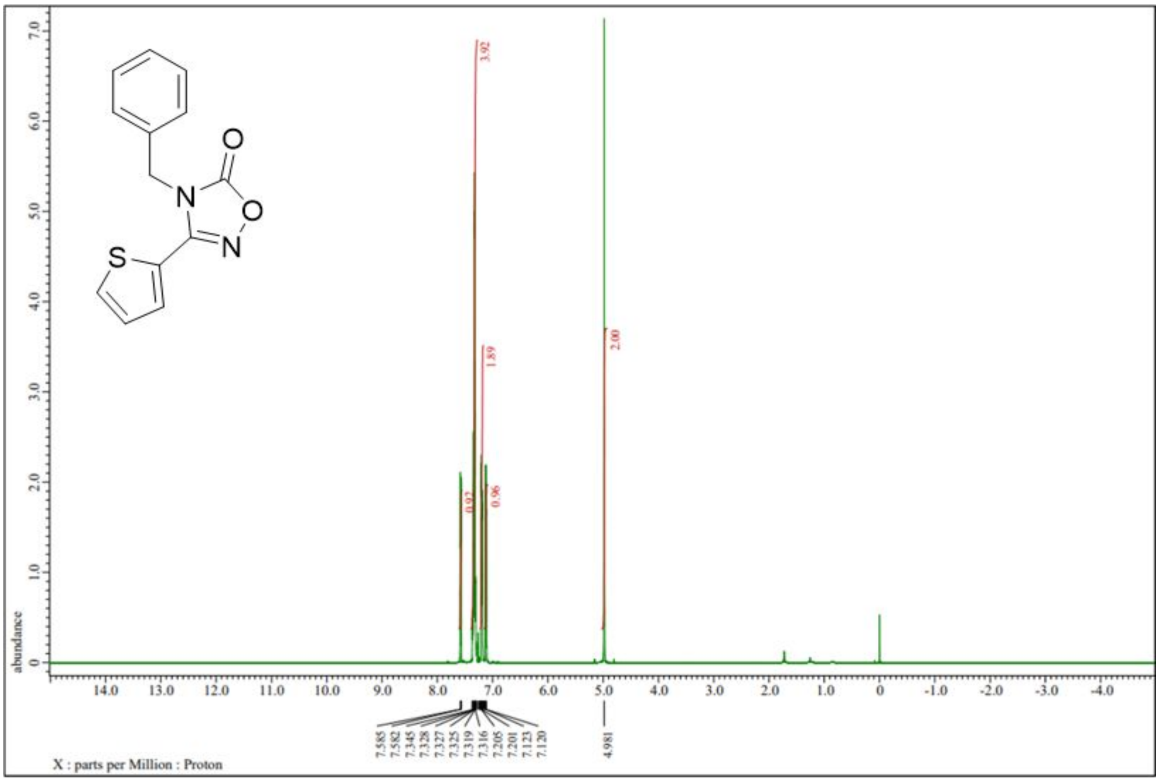

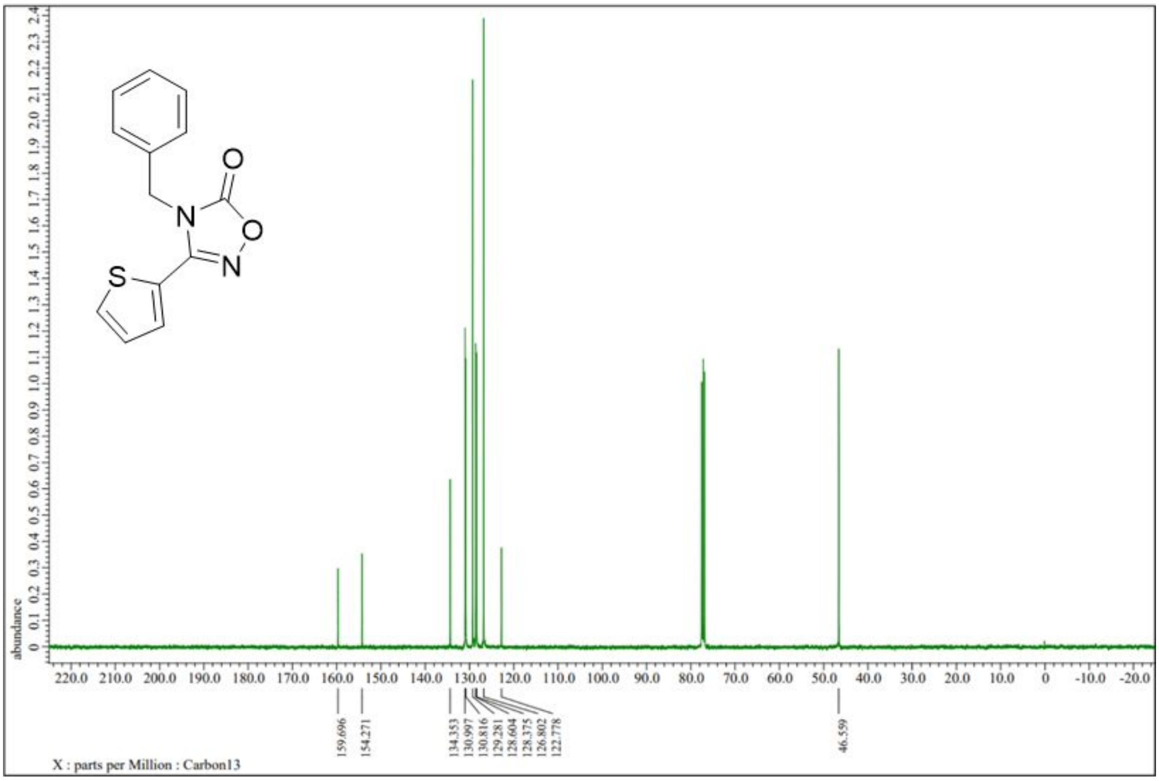

4-(2,4-Dichlorobenzyl)-3-(thiophen-2-yl)-1,2,4-oxadiazol-5(*4H*)-one (12b)

^{1}H NMR, ^{13}C NMR

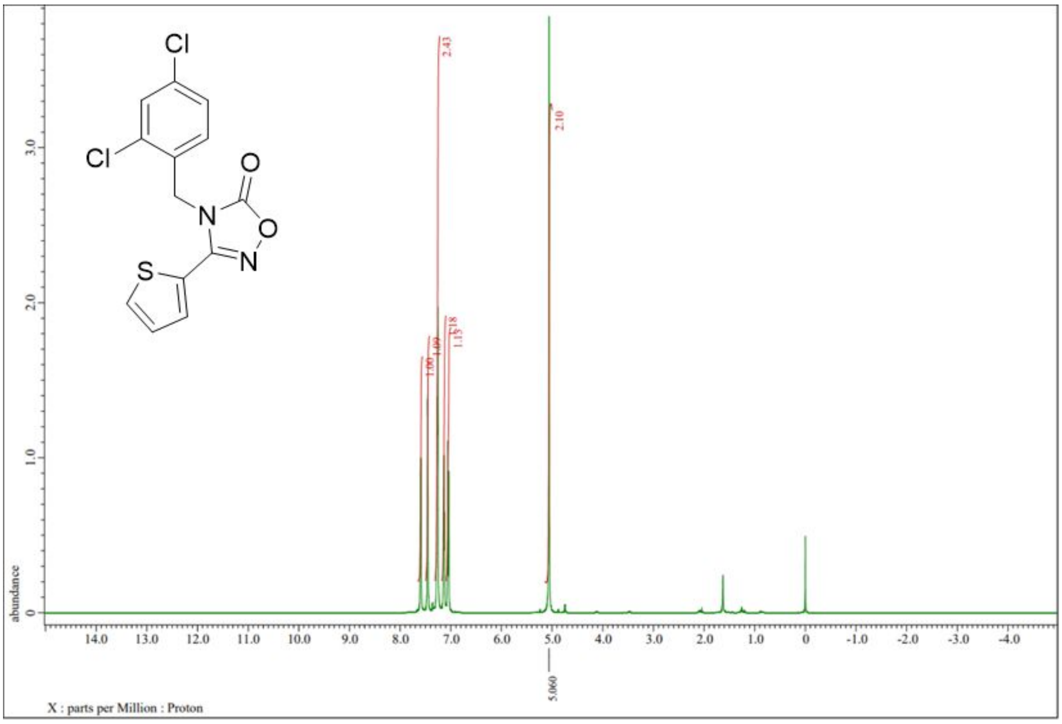

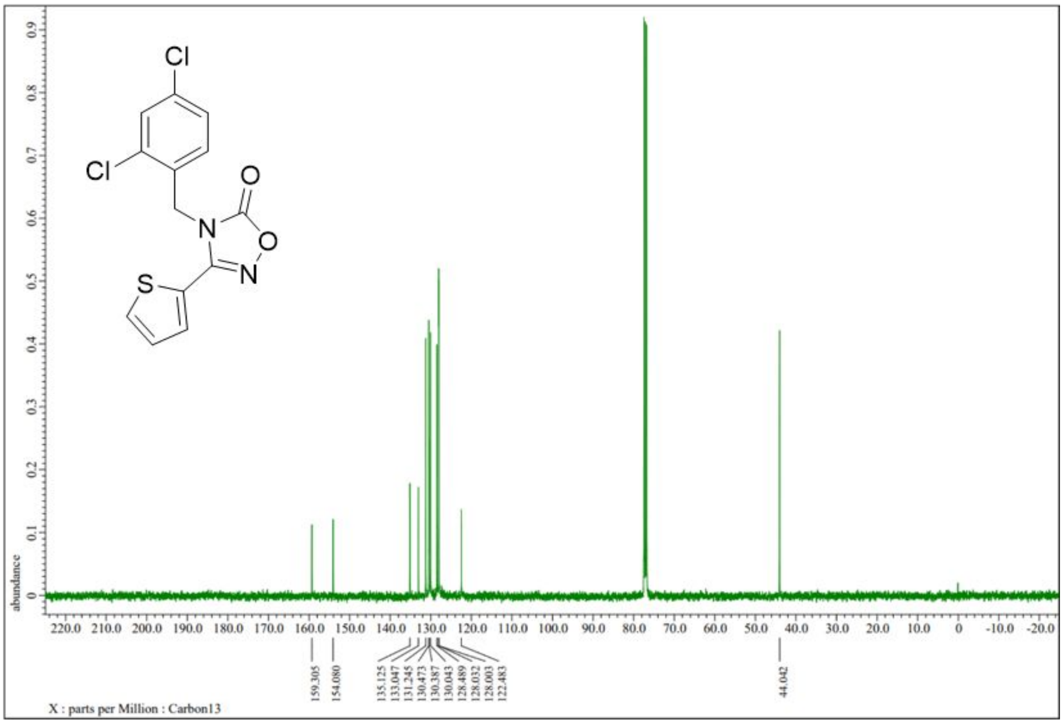

4-(4-Chloro-2-fluorobenzyl)-3-(thiophen-2-yl)-1,2,4-oxadiazol-5(*4H*)-one (12c)

^{1}H NMR, ^{13}C NMR

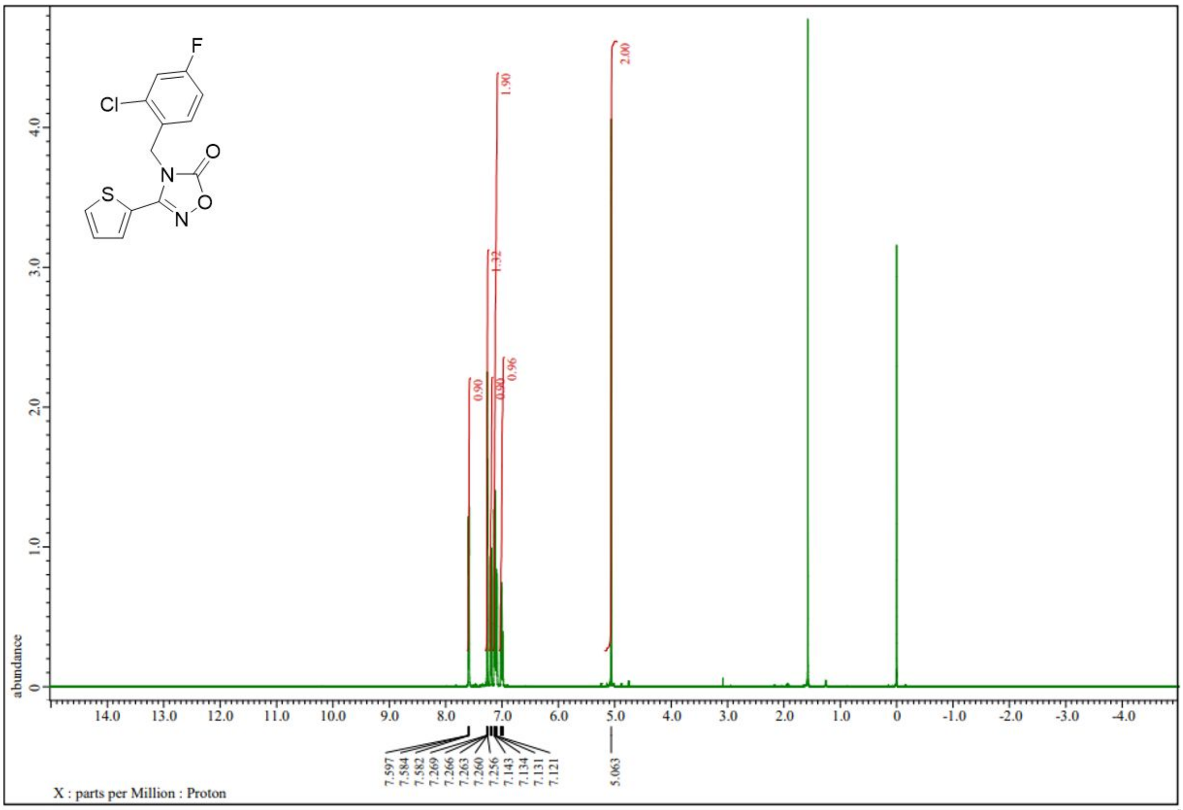

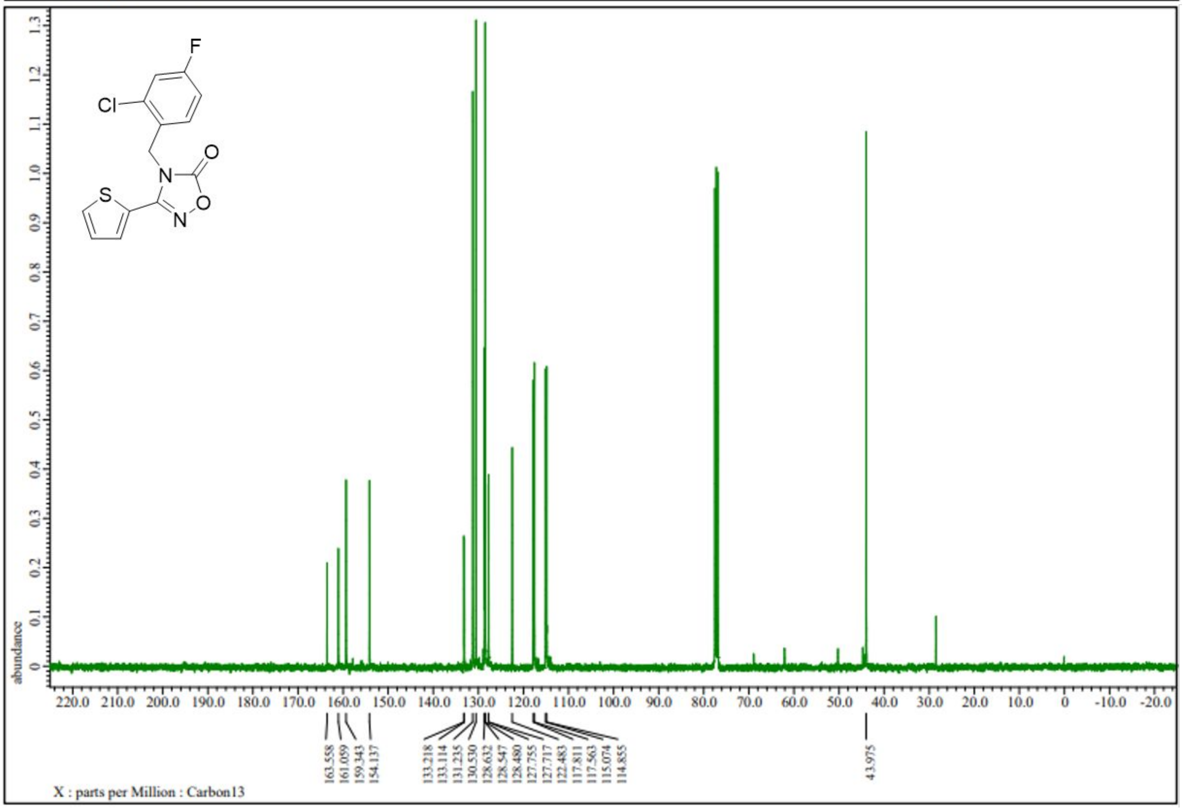

4-(4-Fluorobenzyl)-3-(thiophen-2-yl)-1,2,4-oxadiazol-5(*4H*)-one (12d)

^{1}H NMR, ^{13}C NMR

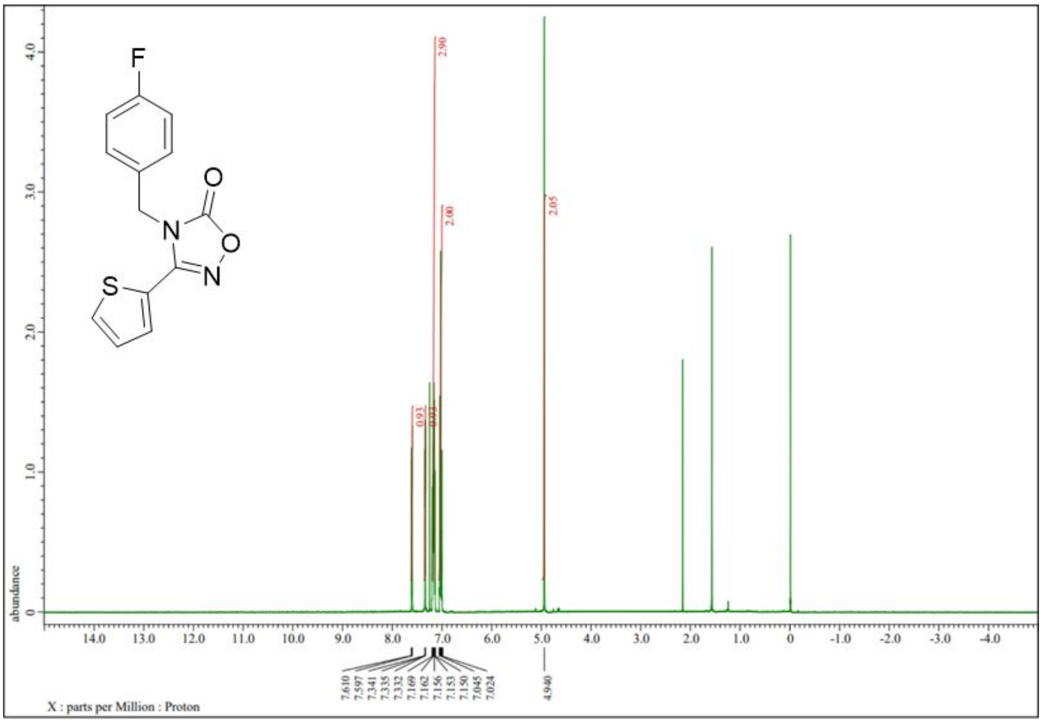

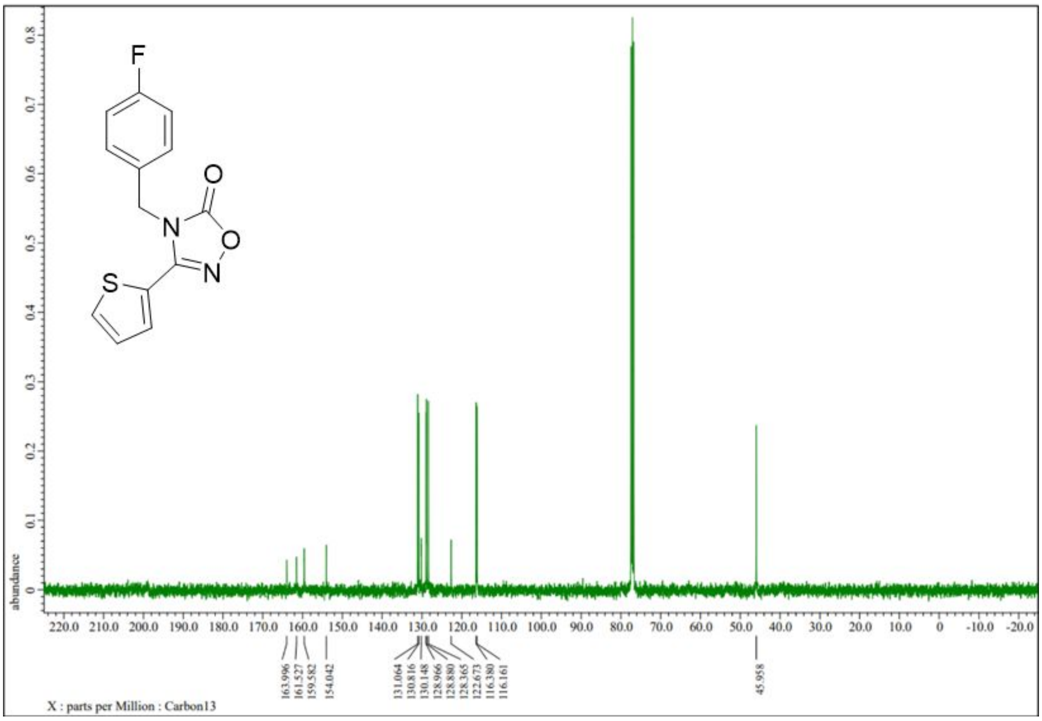

4-(2,4-Difluorobenzyl)-3-(thiophen-2-yl)-1,2,4-oxadiazol-5(*4H*)-one (12e)

^{1}H NMR, ^{13}C NMR

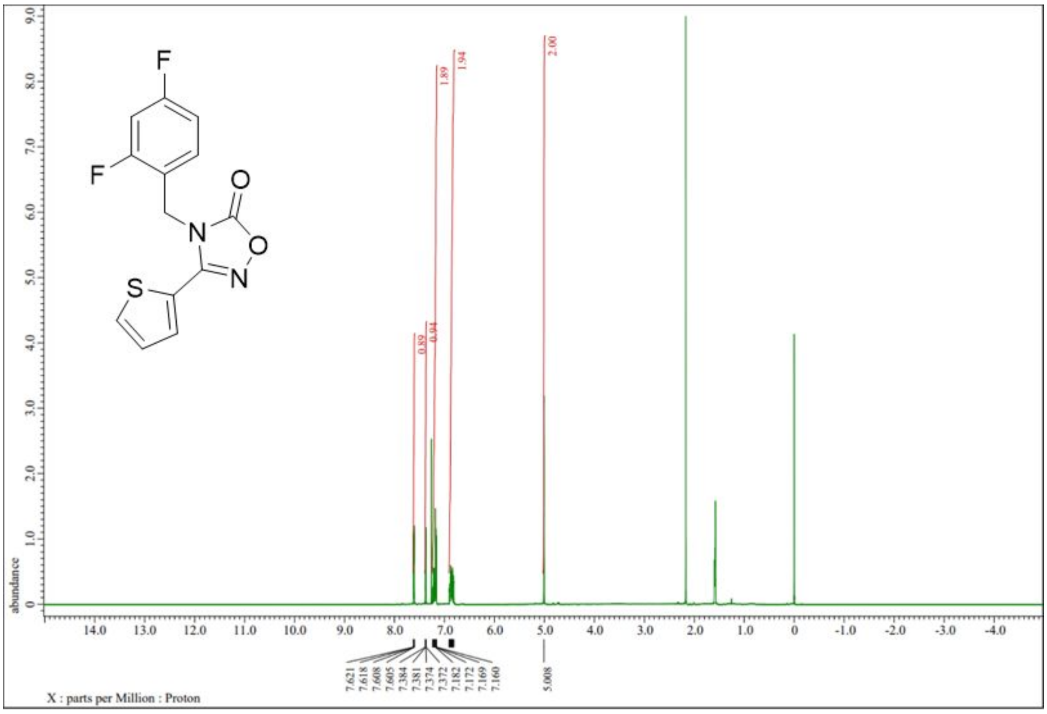

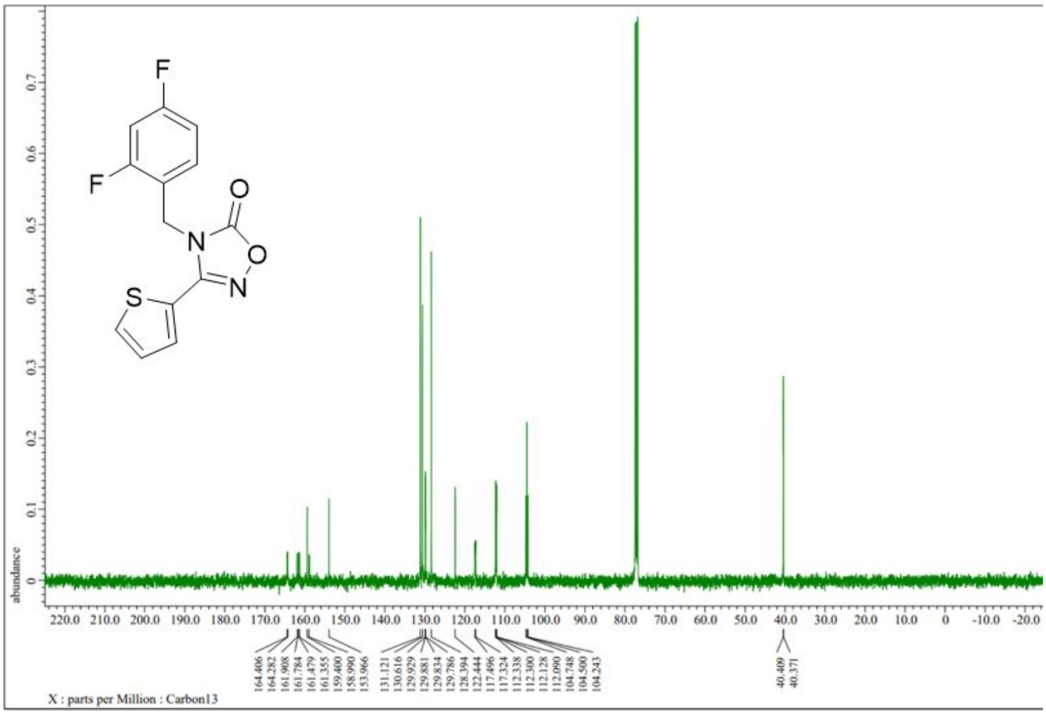

4-(4-Chloro-2-fluorobenzyl)-3-(thiophen-2-yl)-1,2,4-oxadiazol-5(*4H*)-one (12f)

^{1}H NMR, ^{13}C NMR

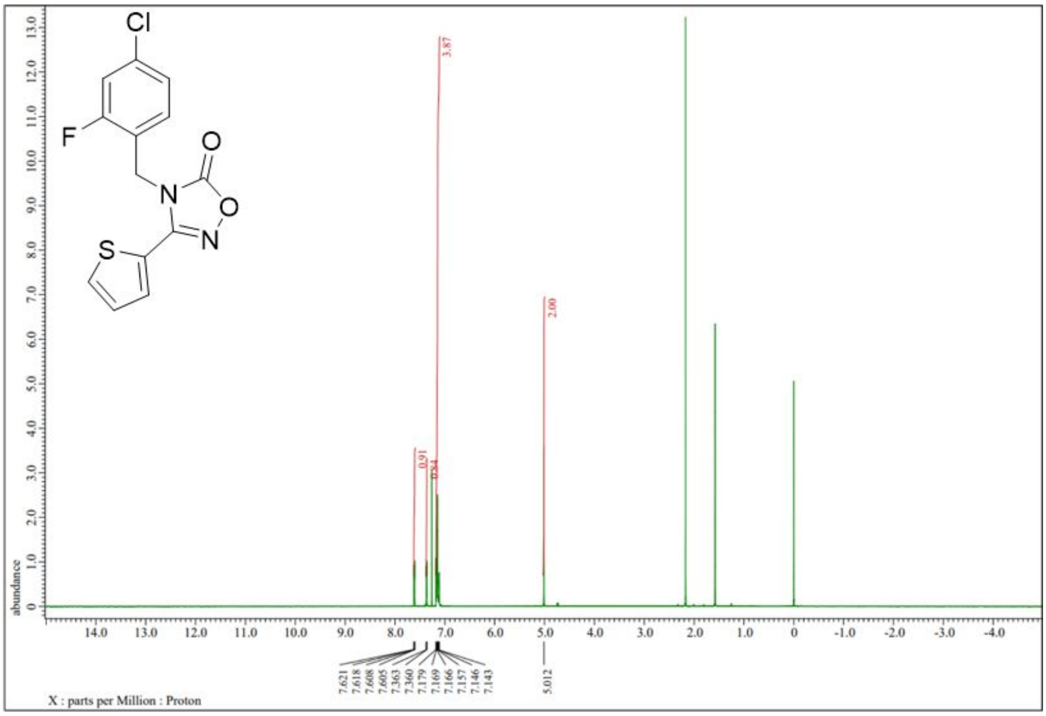

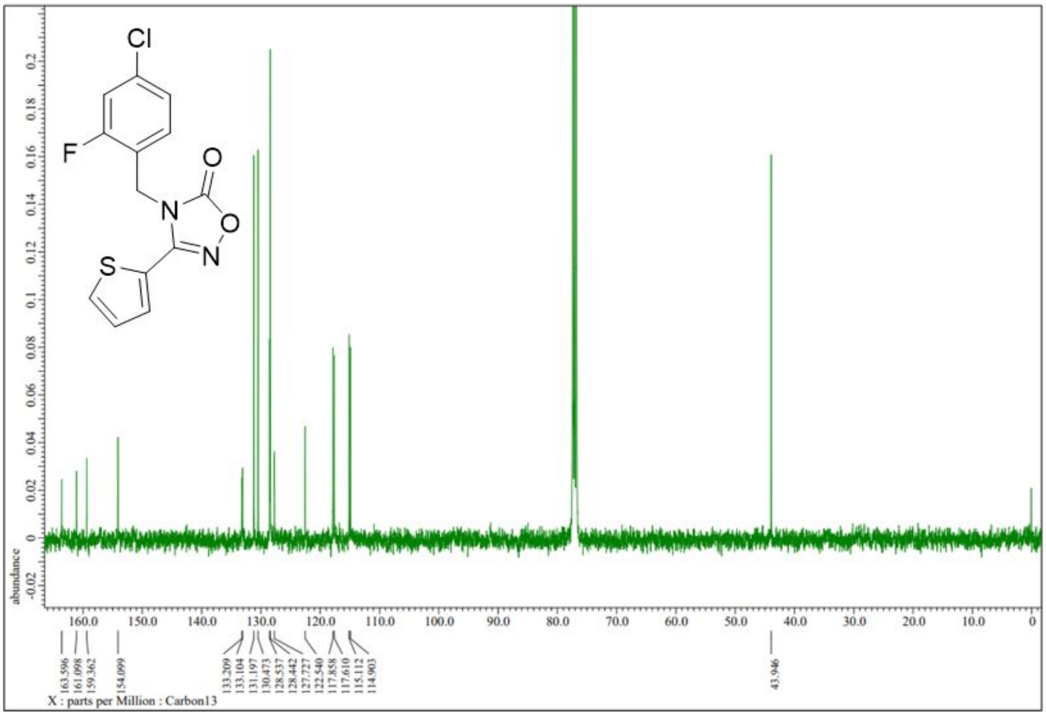

2-(2-Chloro-4-fluorobenzyl)-5-(thiophen-2-yl)-2,4-dihydro-*3H*-1,2,4-triazol-3-one (18)

^{1}H NMR, ^{13}C NMR

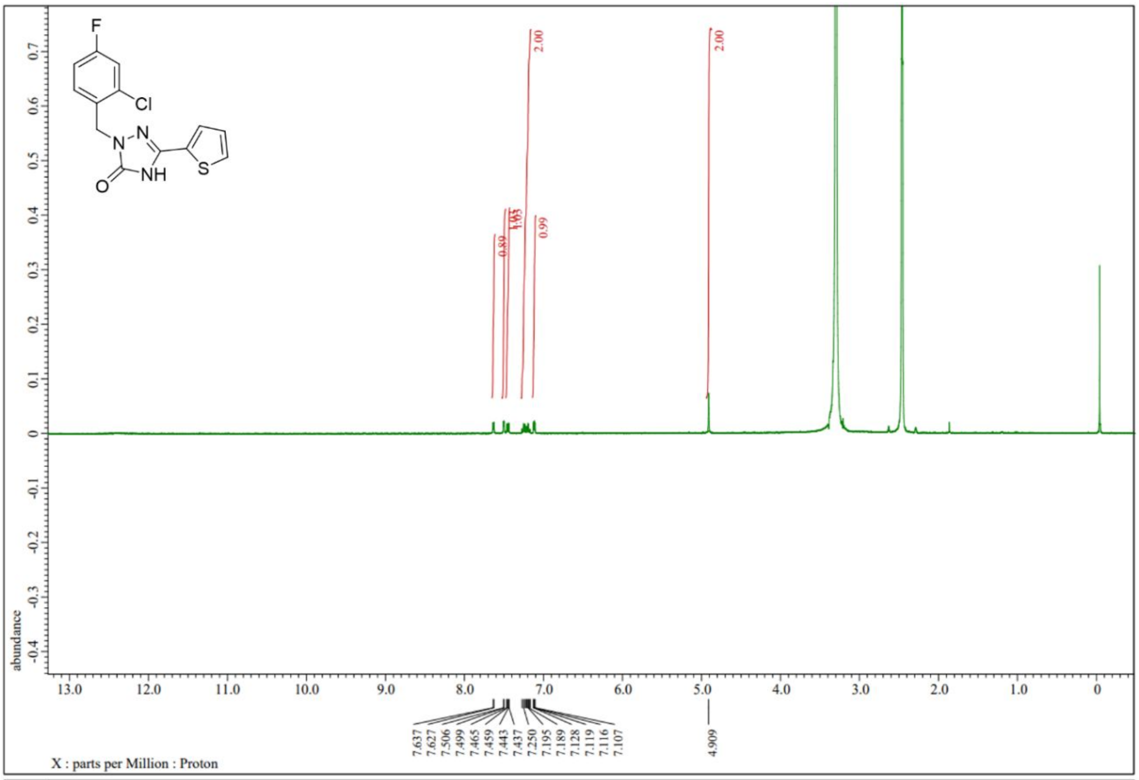

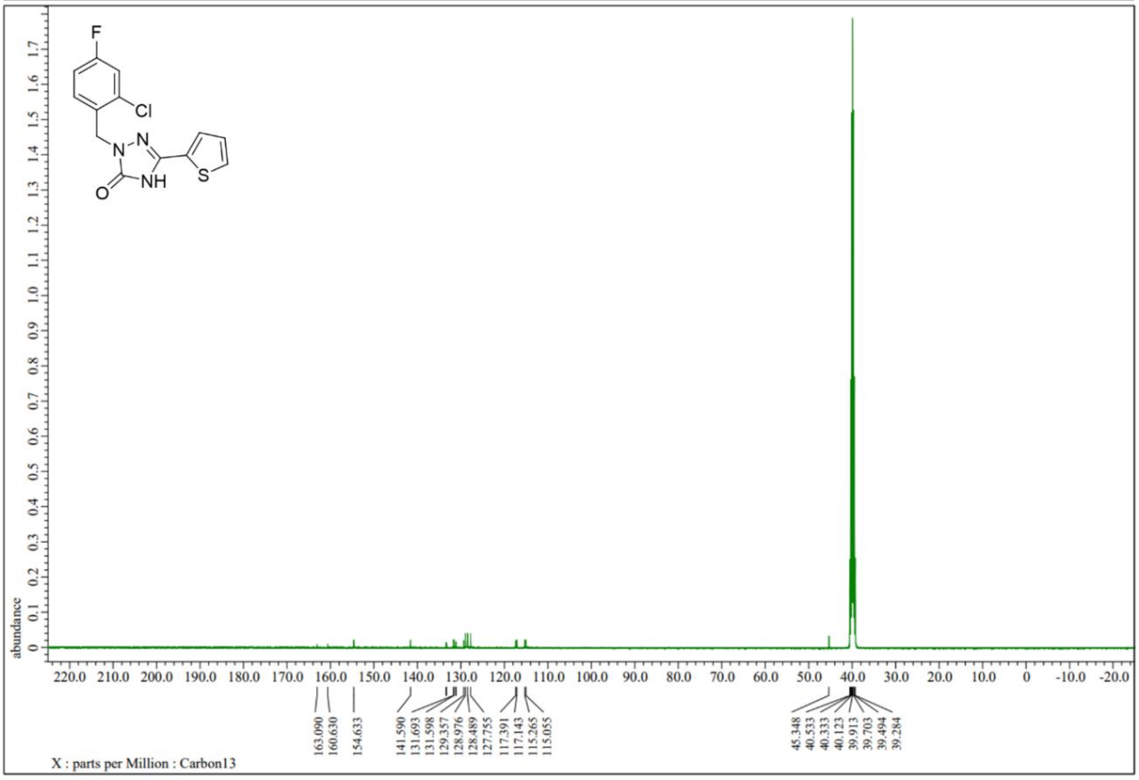

3-(2-Chloro-4-fluorobenzyl)-5-(thiophen-2-yl)-1,3,4-oxadiazol-2(*3H*)-one (20a)

^{1}H NMR, ^{13}C NMR

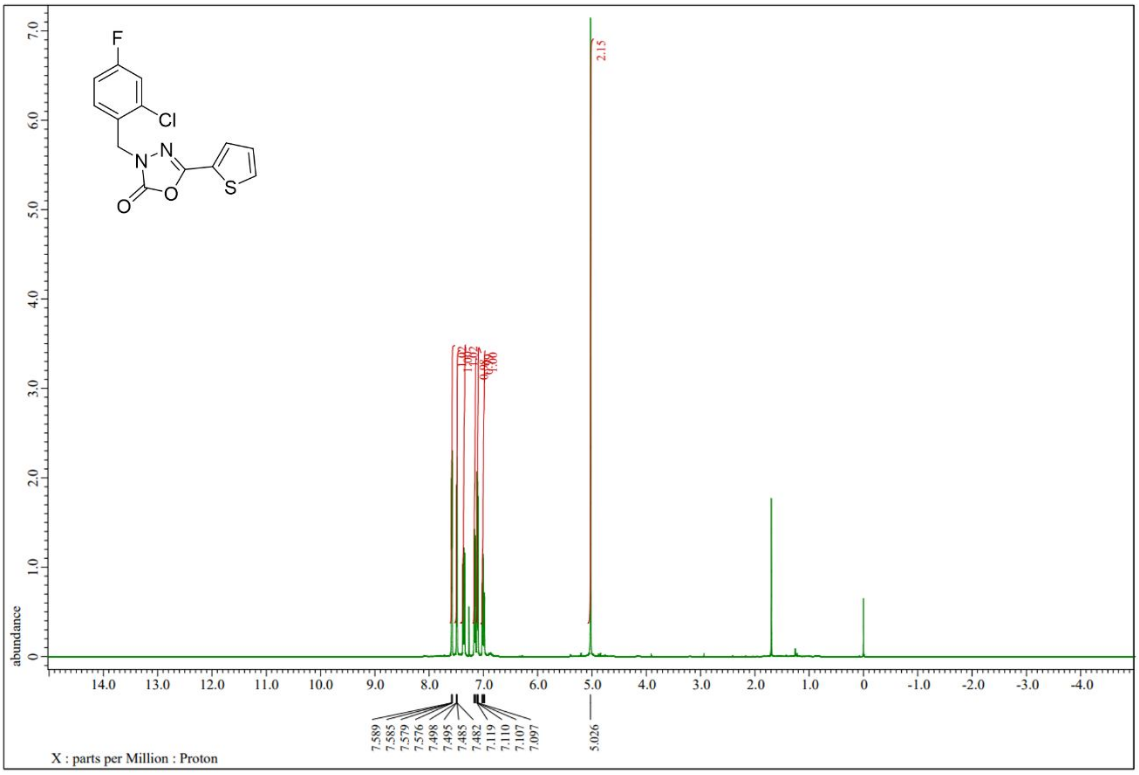

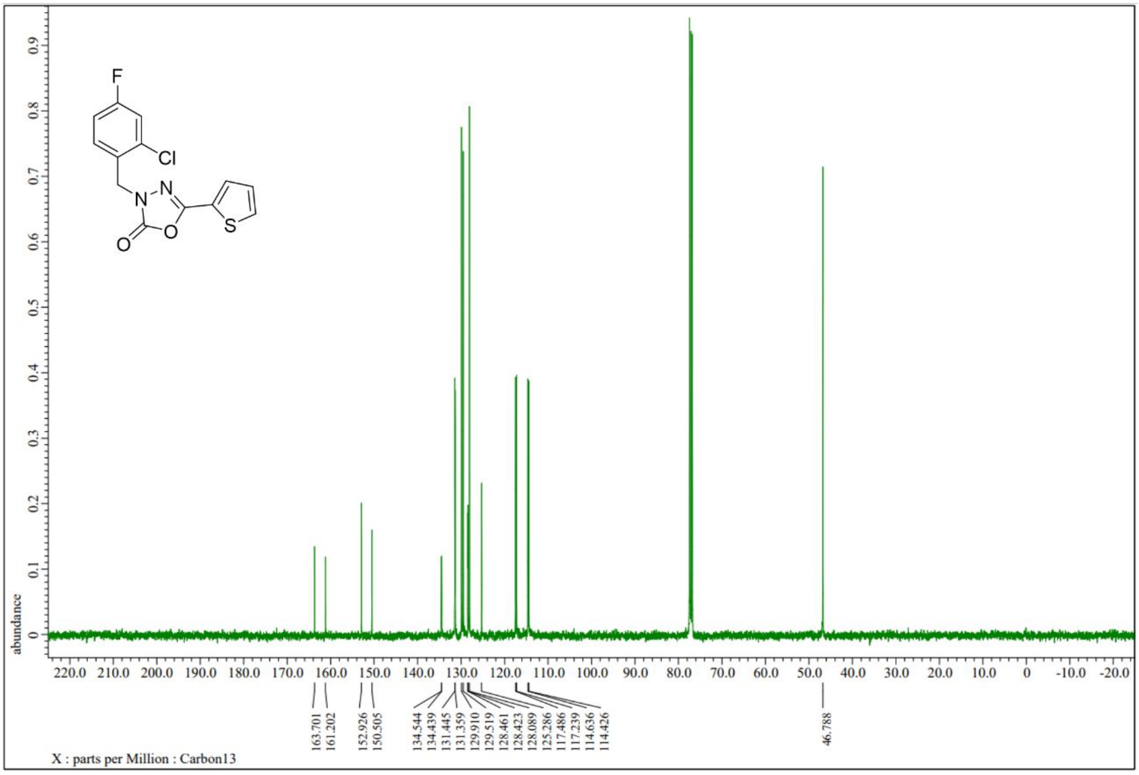

3-(2-Chloro-4-fluorobenzyl)-5-(furan-2-yl)-1,3,4-oxadiazol-2(*3H*)-one (20b)

^{1}H NMR, ^{13}C NMR

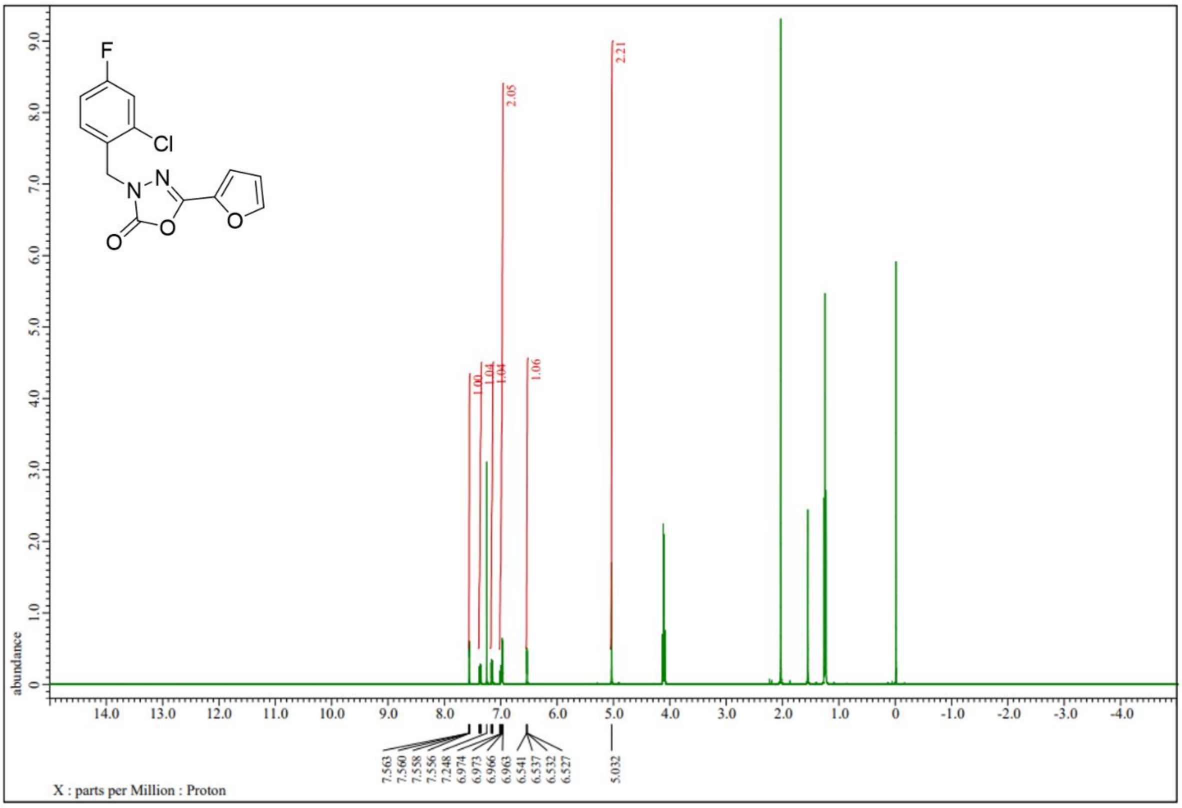

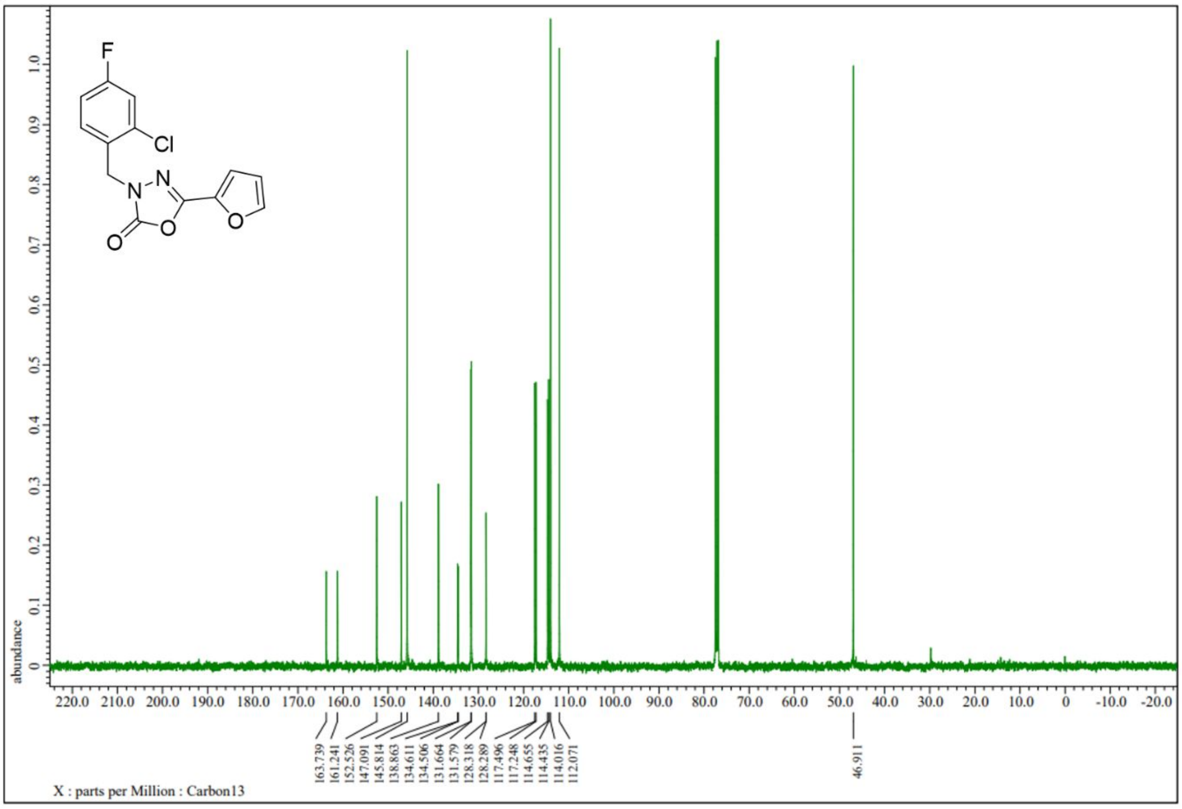

3-(2-Chloro-4-fluorobenzyl)-5-(pyridin-2-yl)-1,3,4-oxadiazol-2(*3H*)-one (20c)

^{1}H NMR, ^{13}C NMR

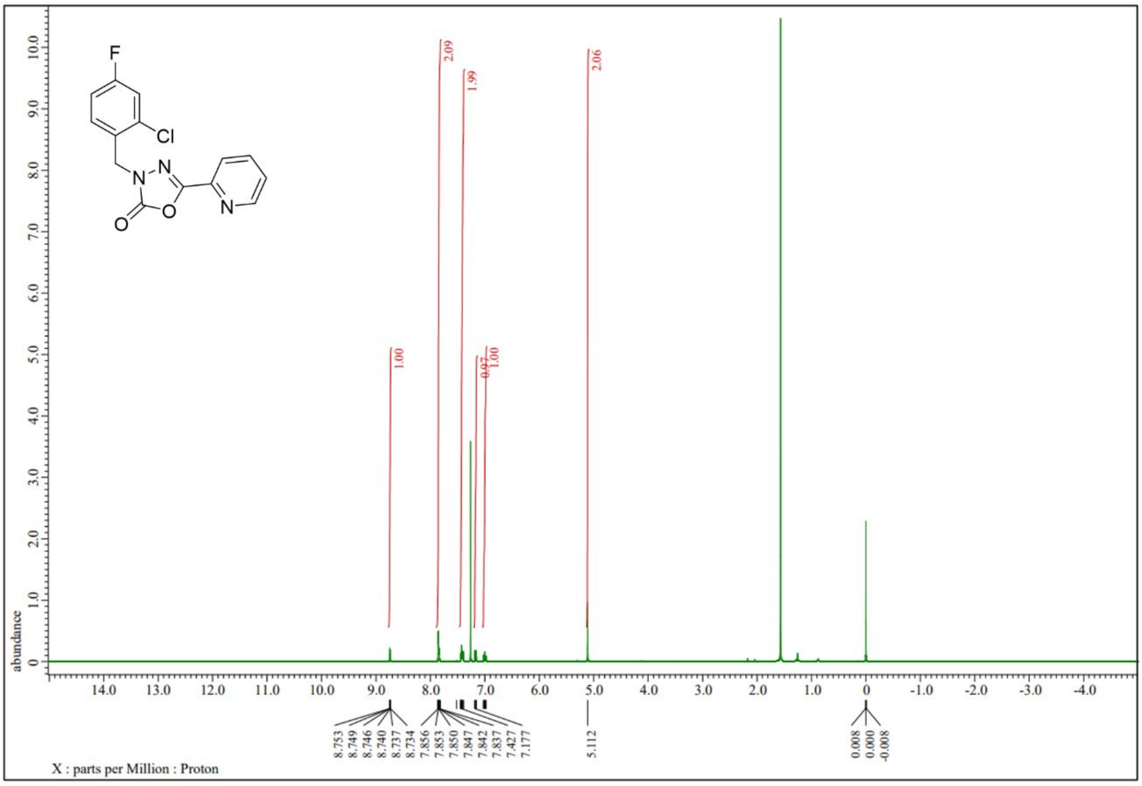

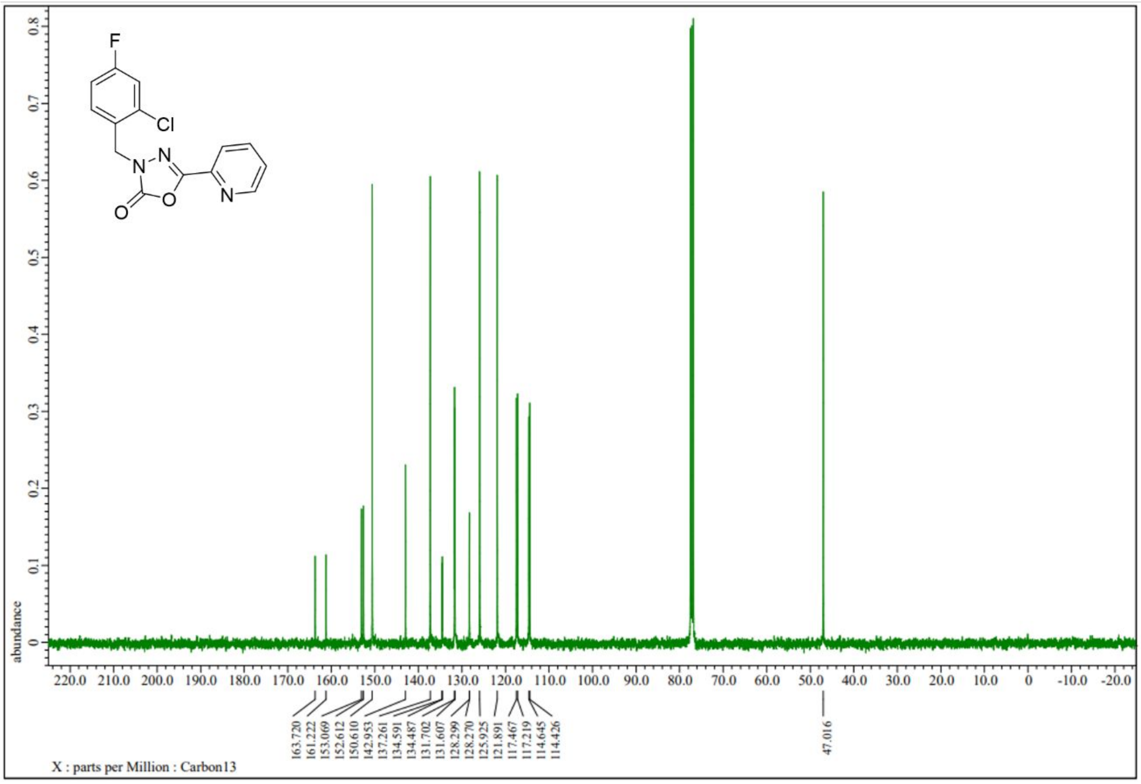

3-(2-Chloro-4-fluorobenzyl)-5-(pyridin-4-yl)-1,3,4-oxadiazol-2(*3H*)-one (20d)

^{1}H NMR, ^{13}C NMR

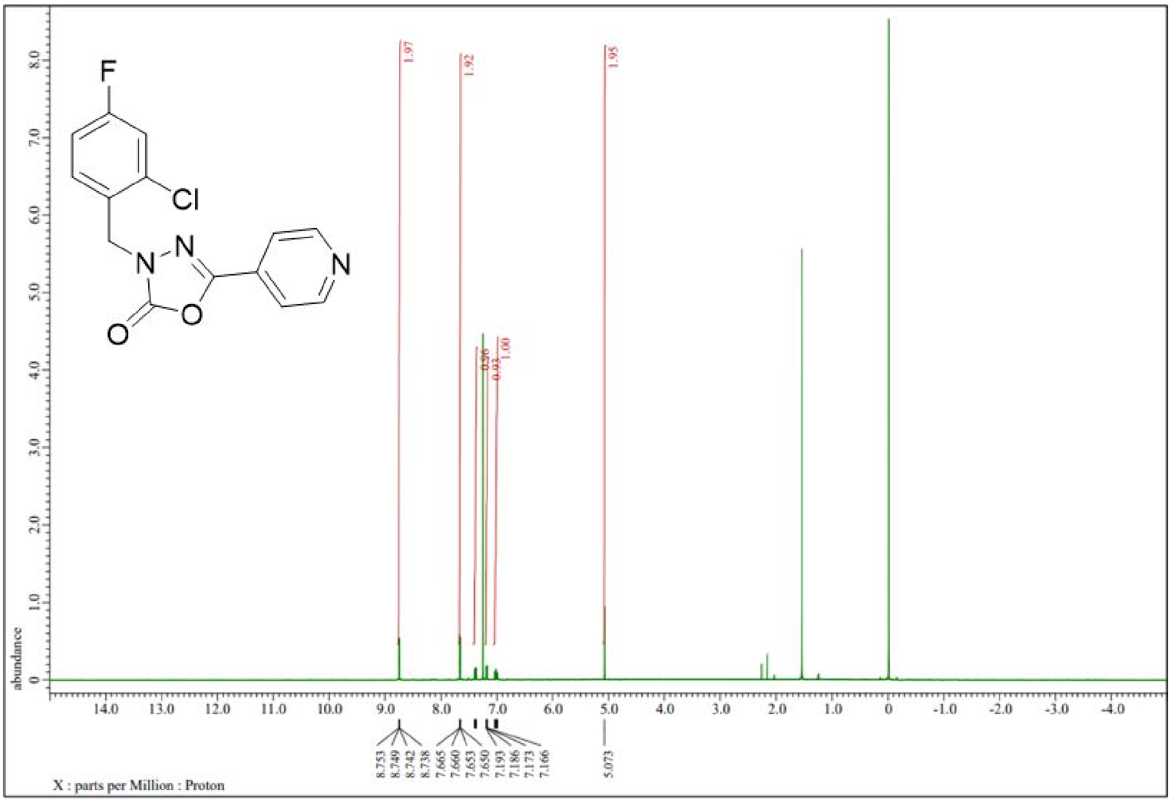

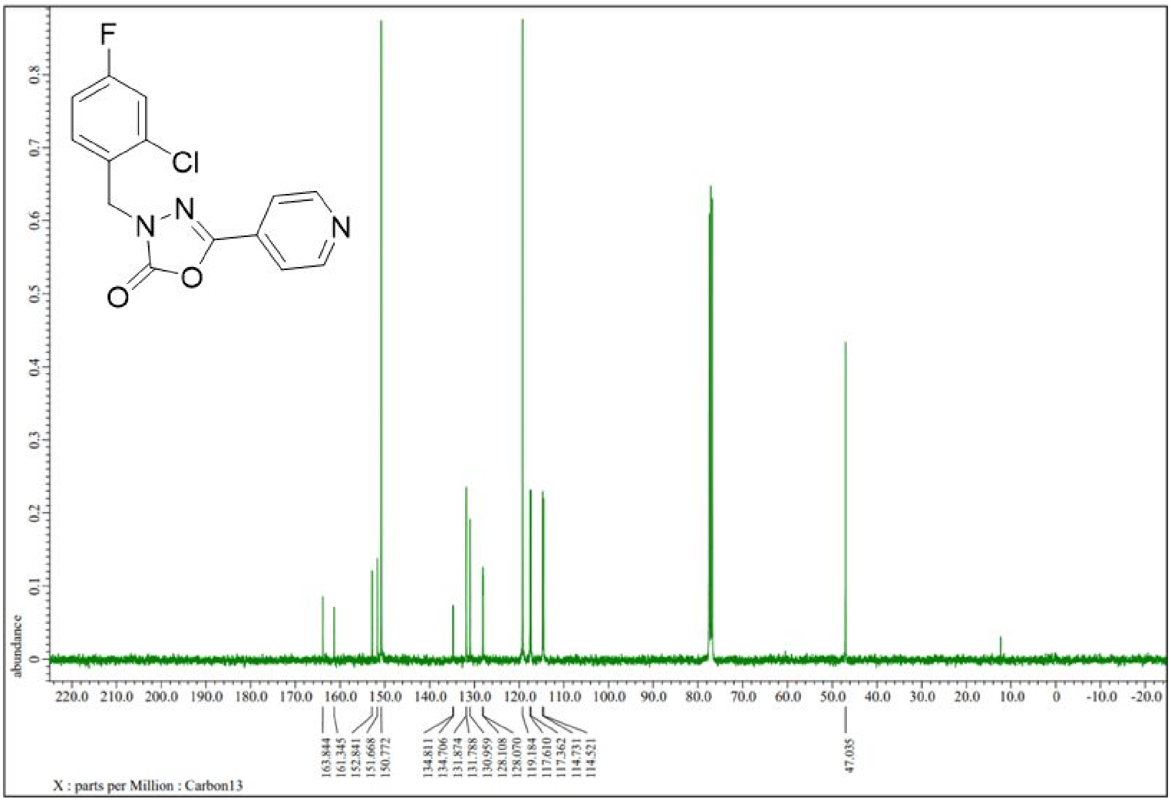

3-(2-Chloro-4-fluorobenzyl)-5-(2-(trifluoromethyl)pyridin-4-yl)-1,3,4-oxadiazol-2(*3H*)-one (20e)

^{1}H NMR, ^{13}C NMR

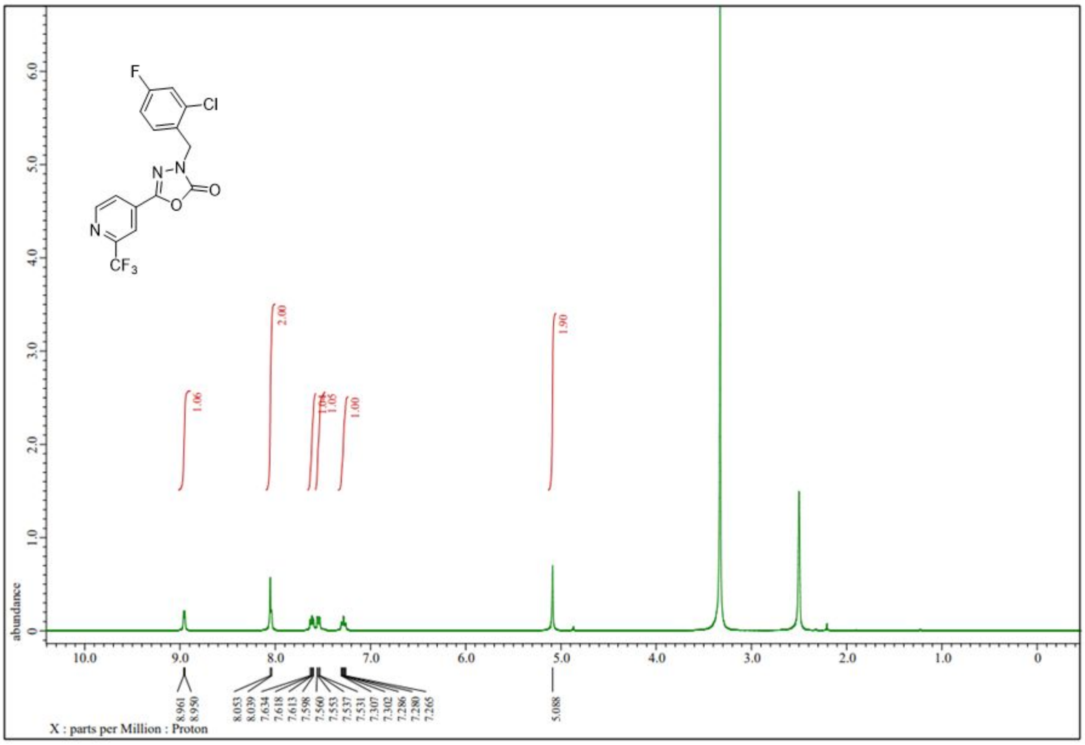

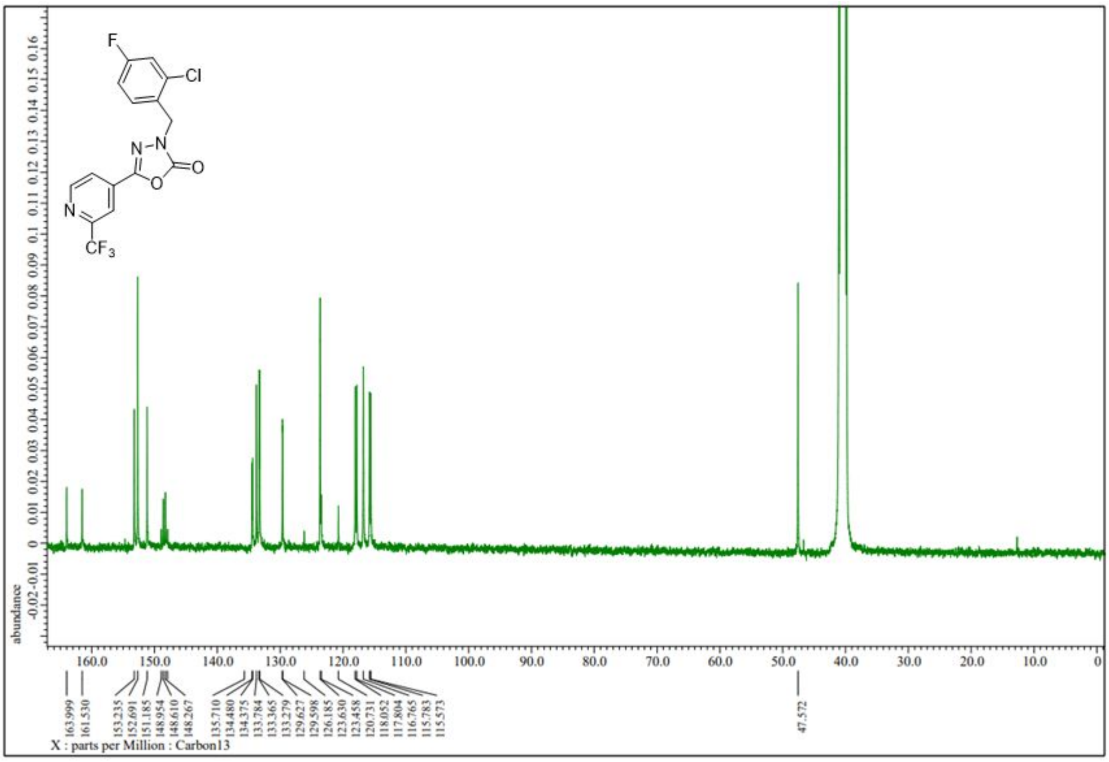

References

(1) Wang, J.-J.; Guo, J.-J.; Zhan, J.; Bu, H.-Z.; Lin, J. H. An in-vitro cocktail assay for assessing compound-mediated inhibition of six major cytochrome P450 enzymes. *Journal of pharmaceutical analysis* **2014**, *4* (4), 270-278.

(2) Liu, W.; Zhang, Y.; Xing, S.; Lan, H.; Chen, X.; Bai, Y.; Shao, X. β-Trifluorosulfinylesters: tuneable reagents for switchable trifluoromethylsulfinylation and C–H trifluoromethylthiolation. *Organic Chemistry Frontiers* **2023**, *10* (9), 2186-2192.
